# Interaction and Flavor Metabolic Function of Microbiota During Fermentation of Pigskin Through Bioaugmentation with *Latilactobacillus sakei*

**DOI:** 10.3390/molecules31111889

**Published:** 2026-06-01

**Authors:** Qi Wang, Lili Ji, Xiaoshan Dong, Shufan Zhang, Kunyi Liu, Jia Zheng

**Affiliations:** 1 School of Wuliangye Technology and Food Engineering, Yibin Vocational and Technical College, Yibin 644100, China; 2 Meat Processing Key Laboratory of Sichuan Province, Chengdu University, Chengdu 610106, China; 3 Wuliangye Yibin Co., Ltd., Yibin 644000, China

**Keywords:** *Latilactobacillus sakei*, bioaugmented fermentation, pigskin, microbial community, volatile flavor compounds, odor activity value

## Abstract

Pigskin, a major byproduct of pork processing, has high protein content and low fat, endowing it with considerable market value for food applications. In this study, bioaugmented fermentation with *Latilactobacillus sakei* YBZY-W5, a strain previously isolated from traditional fermented pigskin, was applied to pigskin to systematically evaluate its effects on physicochemical parameters, microbial community succession, and volatile flavor compound (VFC) profiles over 20 days. The results showed that moisture and pH significantly decreased, while total volatile basic nitrogen (TVB-N) and thiobarbituric acid reactive substances (TBARSs) increased with fermentation time. High-throughput sequencing revealed that *Lactobacilli*, *Fusarium* and *Aspergillus* dominated early fermentation and were gradually replaced by *Bacillus*, *Hanseniaspora* and *Debaryomyces*. A total of 493 VFCs were identified, among which terpenoids, heterocyclic compounds, and alcohols were the most abundant classes. Orthogonal partial least squares discriminant analysis (OPLS-DA) identified numerous differentially changed VFCs (DCVFCs) during fermentation. Odor activity value (OAV) analysis indicated that green, meaty, and woody notes dominated initially, while sour, floral, sweet, and fruity characteristics became increasingly prominent after fermentation. Pearson correlation analysis demonstrated significant associations between key microorganisms (*Lactobacilli*, *Bacillus*, *Hanseniaspora*, *Debaryomyces*) and DCVFCs (e.g., β-myrcene, ethyl hexanoate, hexanoic acid, ethyl ester, pyrazines). Collectively, bioaugmented fermentation with *Ltb. sakei* YBZY-W5 effectively modulated the physicochemical and microbial profiles of pigskin, enriched desirable flavor compounds, and reduced unpleasant odor, confirming its feasibility for producing high-quality fermented pigskin products. This study provides an experimental basis for the value-added utilization of pigskin and promotes sustainable development of the pork industry.

## 1. Introduction

Currently, the global pork market is transitioning from quantitative expansion to qualitative improvement, with increasing emphasis on pork quality, traceability, and the sustainable development of the industry [1]. A substantial amount of pigskin is generated during pig slaughtering, yet as a major byproduct of pork processing, pigskin remains underutilized [2]. The composition of pigskin comprises approximately 65% water, 33% protein, 2% fat, and 0.5% minerals; the protein fraction is dominated by collagen, with smaller amounts of albumin, globulin, and elastin [3]. As a food raw material rich in protein and low in fat with certain health-promoting functions, pigskin holds considerable market potential and application value. It can be processed into standalone pigskin-based food products, added to sausages, ham, and other meat products to enhance texture and quality, or further processed into gelatin and collagen peptides for food applications [4,5,6]. However, limitations in processing technology, equipment, and other conditions have resulted in a low degree of high-value utilization of pigskin. Therefore, in-depth research on pigskin and pigskin products is urgently needed to achieve high-value utilization.

Fermented pigskin is a traditional fermented food produced by adding cooked rice flour, spices, salt, and other seasonings to cooked pigskin, followed by sealing and natural fermentation. Owing to its moderate saltiness and acidity, palatable taste, and distinctive sensory characteristics, it is highly appreciated by consumers in Southwest China [7]. During spontaneous fermentation of pigskin, a complex and dynamic consortium of bacteria and fungi develops, primarily derived from raw materials, spices, and the processing environment. This microbiota plays a pivotal role in determining the final product’s safety, texture, and flavor profile [8]. However, during the production of fermented pigskin, the influence of various raw materials, production utensils, and processing environments leads to the formation of complex and dynamic microbial communities, which in turn generate a diverse and variable profile of flavor compounds. This often results in inconsistent quality of fermented pigskin. Hence, there is an urgent need to improve the traditional production process.

With the increasing demand for food health, bioaugmented fermentation technology has been successfully applied in the traditional fermented food industry in the 1990s, and bioaugmented fermentation refers to adding high-performance strains to traditional fermented foods to quickly make the inoculated strains the dominant microorganisms [9,10]. This approach not only quickly accelerates the fermentation process, but also effectively inhibits the growth of harmful microorganisms, thereby improving product quality stability while ensuring the safety of product [11,12]. The strains used for bioaugmented fermentation must be safe and exhibit metabolic activities relevant to fermented products; ideally, they are native microorganisms isolated and purified from artisanal or local products, as they are well-adapted to the ecological, environmental, and processing conditions and can more effectively dominate the microbial flora present in the product [13,14].

Currently, studies have shown that lactic acid bacteria (LAB), as key participants in fermented meat products, are widely used, and they not only positively influence the formation of flavor, color, texture, and other quality attributes but also rapidly acidify the matrix and inhibit the growth of harmful microorganisms in meat by producing organic acids, hydrogen peroxide, and bacteriocins, thereby improving the safety of fermented meat products and extending their shelf life [15,16]. Xiao et al. used *Lactiplantibacillus plantarum* R2 for bioaugmented fermentation, significantly increasing the total free amino acid and free fatty acid contents of fermented meat products and markedly elevating the levels of alcohols, acids, and esters, while newly generating volatile flavor compounds such as benzaldehyde, acetic acid, 2-pentylfuran, and ethyl esters [17]. Wu et al. inoculated *Leuconostoc mesenteroides* subsp. IMAU: 80,679 for bioaugmented fermentation of meat broth and found that the bioaugmented fermentation inhibited myoglobin oxidation to some extent and increased the contents of nitrosomyoglobin and protoporphyrin [18]. George et al. selected *Lpb. plantarum* NJAU-01 isolated from Jinhua ham as an enhanced starter culture and evaluated the effects of different concentrations of this strain on the color, texture, and sensory properties of dry-cured fermented sausages, and they observed that after 14 and 21 days of bioaugmented fermentation, the redness and hardness of the ham were significantly higher than those of the control group [19]. Slima et al. used *Lpb. plantarum* TN8 for bioaugmented fermentation and found that an inoculum of 10^7^ CFU/g inhibited the growth of *Salmonella* and *Listeria* and maintained lipid oxidation, thus serving as a biopreservative for beef sausages [20]. Furthermore, bacteriocins produced by *Latilactobacillus sakei* exhibit high antibacterial activity, rapidly controlling the growth of *Listeria* and *Enterobacteriaceae*, and minimizing contamination of fermented meat [21]. LAB, particularly the genera *Lactobacilli* and *Pediococcus*, produce organic acids, mainly lactic acid and acetic acid, which acidify the meat batter, inhibit the growth of most harmful foodborne microorganisms, and reduce the formation of biogenic amines as well as the residual levels of nitrite and nitrosamines in fermented meat products [22,23]. In addition to LAB, *Bacillus* and yeast such as *Debaryomyces* and *Hanseniaspora* are also key functional microorganisms in fermented meat products. Specifically, *Bacillus* have strong extracellular protease and lipase activities, which can deeply hydrolyze collagen and lipids, producing a large amount of free amino acids, short peptides, and free fatty acids, providing precursor substances for characteristic VFCs; meanwhile, its metabolic activity can promote pigment stability and texture improvement. Yeast catalyzes the production of esters, higher alcohols, and sulfur-containing compounds through esterase activity and amino acid metabolism pathways, endowing fermented meat products with floral, fruity, and meaty VFCs [24]. Furthermore, the microbial community forms a complex metabolic interaction network through quorum sensing, cross feeding, and niche construction. For instance, LAB rapidly produce acid, creating selective pressure for acid-resistant microorganisms and inhibiting pathogens, while vitamins, amino acids, and dead cell autolysis produced by yeast metabolism can in turn promote bacterial growth [25]. Building on this understanding of native microbial interactions, bioaugmented fermentation is novel in its ability to actively steer microbial succession and stabilize product quality compared to spontaneous fermentation. Nevertheless, its limitations include uncertain competitive establishment of the inoculated strain, potential disruption of native microbial networks, and lack of long-term ecological stability data. These factors necessitate case-specific validation.

Our research team previously isolated and purified a strain of *Ltb. sakei* YBZY-W5 from traditional pigskin fermentation, and whole-genome sequencing results indicated that *Ltb. sakei* YBZY-W5 possesses strong biofilm-forming ability; can efficiently utilize carbohydrates, amino acids, and nucleotides; and does not carry any pathogenic genes. Therefore, *Ltb. sakei* YBZY-W5 is safe and exhibits favorable fermentation characteristics, making it suitable for use as a starter culture in fermented meat products. In this study, we intend to use *Ltb. sakei* YBZY-W5 for bioaugmented fermentation of pigskin. By analyzing the physicochemical parameters, microbial community structures and volatile flavor compounds of samples, and elucidating the participation mode and metabolic function of microbial communities during pigskin fermentation, we aim to evaluate the feasibility of applying this strain for bioaugmented fermentation of pigskin, provide experimental evidence for improving the quality of fermented pigskin, and promote the sustainable development of the pork industry.

## 2. Results and Discussion

### 2.1. Analysis of Physicochemical Parameters During the Fermentation of Pigskin

**Figure 1:**
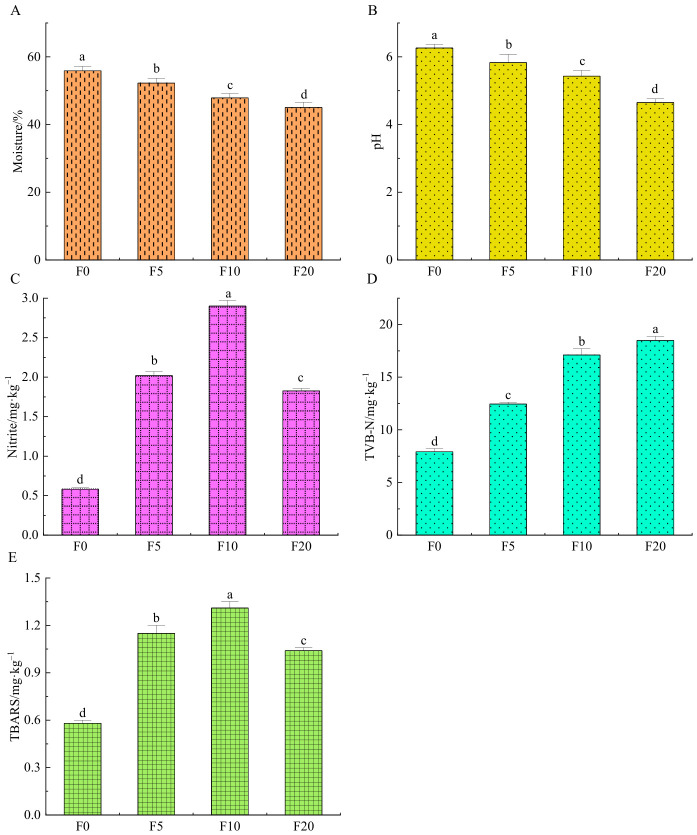
Variation in physicochemical parameters during fermentation of pigskin; different letters indicate significant differences in the same physicochemical parameter (*p* < 0.05). (**A**) The content of moisture of fermented pigskin; (**B**) pH of fermented pigskin; (**C**) the content of nitrite of fermented pigskin; (**D**) The content of TVB-N of fermented pigskin; (**E**) the content of TBARS of fermented pigskin.

The appearance of pigskin has changed after fermentation by microorganisms mainly composed of *Ltb. sakei*. For this purpose, the physicochemical parameters, microbial communities, and VFCs of pigskin were analyzed during the fermentation process. As illustrated in Figure 1, the fermentation process induced significant alterations in the physicochemical parameters of pigskin. With the continuous extension of fermentation time, the content of moisture significantly decreased (*p* < 0.05, Figure 1A), which is consistent with typical fermented food processes, where water loss results from evaporation and osmotic pressure changes induced by salt and metabolites [26,27]. Notably, the pH of pigskin also exhibited a decreasing trend (Figure 1B), which is primarily attributable to the metabolism of lactic acid and other organic acids by acid-producing microorganisms such as *Lactobacilli* during fermentation [28]. This acidification is a critical mechanism for suppressing spoilage bacteria and ensuring fermented product safety. However, the content of nitrite significantly increased in the first ten days of fermentation (*p* < 0.05), but significantly decreased on the 20th day of fermentation (*p* < 0.05, Figure 1C). The initial increase may originate from nitrate reduction by indigenous bacteria or from added soy sauce, while the subsequent decline is likely due to enzymatic degradation and chemical decomposition under acidic conditions, ensuring final nitrite levels within safety limits (GB 5009.33-2016) [29]. TVB-N, which refers to alkaline nitrogen-containing compounds produced by endogenous proteases and microbial degradation of proteins, significantly increased with fermentation time (*p* < 0.05, Figure 1D). This indicates active proteolysis, which is essential for generating free amino acid precursors of volatile flavor compounds. Moderate oxidation of fat is beneficial for the formation of characteristic flavors in meat products, while excessive oxidation can have a negative impact on flavor [30]. As shown in Figure 1E, the change trend of TBARS was the same as nitrite. The parallel trends suggest a possible interplay between lipid oxidation and nitrite-derived antioxidant effects. By comparison, the trends of changes in all the physicochemical parameters during the fermentation of pigskin were basically consistent with that of sausages, and the physicochemical parameters met the safety standards of fermented meat products [25].

### 2.2. Microbiological Analyses During the Fermentation of Pigskin

**Figure 2:**
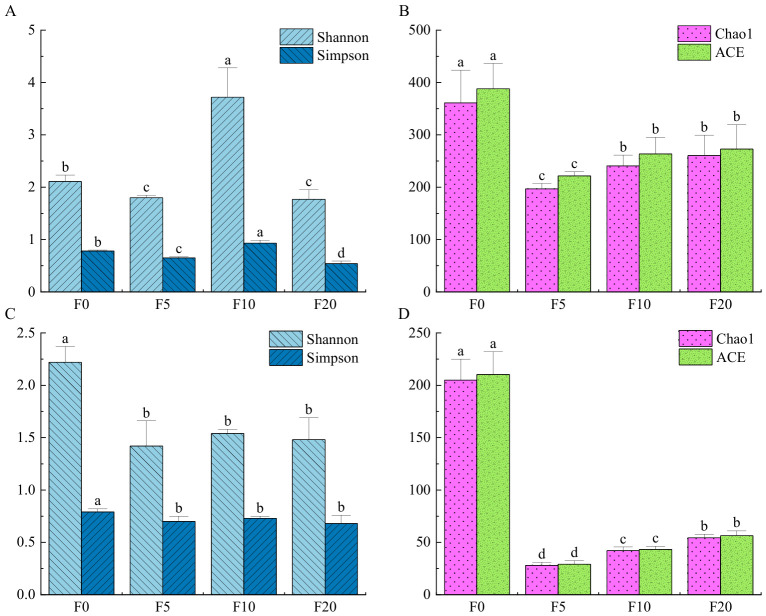
Alpha diversity index of microbial community during fermentation of pigskin. Shannon and Simpson indices in bacteria (**A**); Chao1 and ACE indices in bacteria (**B**); Shannon and Simpson indices in fungi (**C**); Chao1 and ACE indices in fungi (**D**). Different letters indicate significant differences in the same alpha diversity index (*p* < 0.05).

Quality control was performed on the offline data obtained from Illumina NovaSeq sequencing to obtain valid data, and the coverage rate of all samples was 99.99%, indicating sufficient sequencing depth for reliable community analysis. Among them, the effective data of bacteria were 58,627~112,759 sequences, classified as 174~379 bacterial amplicon sequence variants (ASVs); the effective data for fungi were 75,219~121,142 sequences, classified as 39~226 fungal ASVs. These findings offer a detailed, precise depiction of the microbial variety, reflected in the coverage index for fermentation samples. The alpha diversity (Figure 2) was used to assess the microbial biodiversity. Shannon and Simpson index represent the diversity of species, which extend to multispecies metacommunities, while Chao1 and ACE indices represent the richness and are widely accepted measures of ecological diversity for species richness [31,32].

As shown in Figure 2A, the diversity of bacteria (Shannon and Simpson index) decreased significantly first, then increased significantly, and finally decreased significantly as fermentation progresses (*p* < 0.05). Meanwhile, the richness of bacteria (Chao1 and ACE indices) decreased significantly, then increased significantly (*p* < 0.05) with the fermentation process and stabilized after 10 d (Figure 2B). This three-phase pattern (suppression → rebound → stabilization) reflects the initial dominance of the bioaugmented *Lactobacilli*, followed by adaptation of surviving taxa under oxygen depletion, and finally establishment of a stable consortium. Furthermore, the diversity of fungi decreased significantly first (*p* < 0.05) and stabilized after 5 d (Figure 2C), while, the richness of fungi decreased significantly, then increased significantly (*p* < 0.05) with the fermentation process (Figure 2D). The rapid decline in fungal diversity after day 5 is a positive safety outcome, as it indicates suppression of potential mycotoxin-producing molds by the acidifying environment.

**Figure 3:**
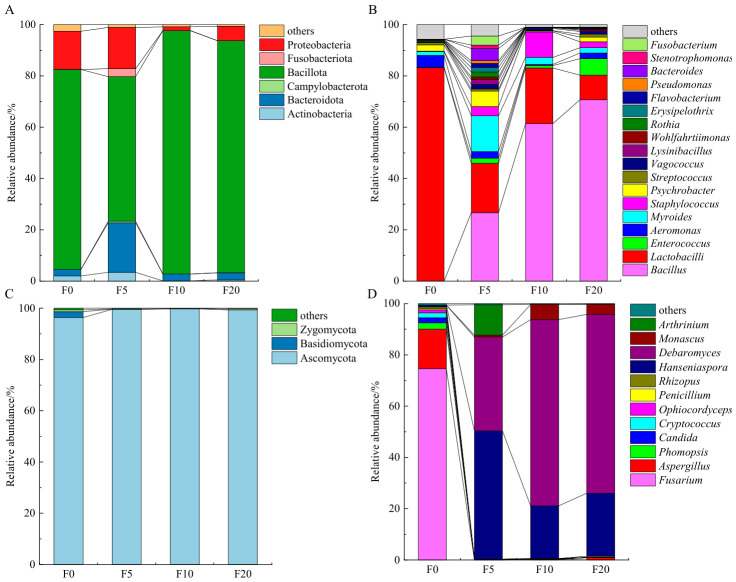
The relative abundance percentage during fermentation of pigskin. The phylum level of bacteria (**A**), the genus level of bacteria (**B**), the phylum level of fungi (**C**), the genus level of fungi (**D**).

Figure 3 illustrates microbial community dynamics at phylum and genus levels. Six major bacterial phyla (relative abundance > 1%), including Actinobacteria, Bacteroidota, Campylobacterota, Bacillota, Fusobacteriota, and Proteobacteria, were identified from fermentation of pigskin (Figure 3A). Among them, Bacillota was the dominant bacterial phylum, with a relative abundance ranging from 77.93% (F0) to 56.36% (F5), reaching the maximum relative abundance at F10 (94.87%), and dropped to 90.47% in the later stage of fermentation (F20). Meanwhile, Bacteroidota and Proteobacteria were also the dominant bacterial phylum, their relative abundance showed an initial increase followed by a decrease as fermentation progressed. Dan et al. found that after incubating pigskin at different temperatures (25 °C and 37 °C) for 7 days, Bacillota (formerly Firmicutes) and Proteobacteria were dominant bacteria [33]. The temporary decrease at day 5 may be due to the initial presence of Bacteroidota and Proteobacteria from raw materials, which were subsequently outcompeted. At the genus level, a total of 18 major genera were identified (Figure 3B), including *Bacillus*, *Lactobacilli*, *Enterococcus*, *Aeromonas*, *Myroides*, *Staphylococcus*, *Psychrobacter*, *Streptococcus*, *Vagococcus*, *Lysinibacillus*, *Wohlfahrtiimonas*, *Rothia*, *Erysipelothrix*, *Flavobacterium*, *Pseudomonas*, *Bacteroides*, *Stenotrophomonas*, and *Fusobacterium*. Due to bioaugmented fermentation with *Ltb. sakei* YBZY-W5, the relative abundance of *Lactobacilli* was highest in the early stages of fermentation (83.10%). As fermentation progresses, the relative abundance of *Lactobacilli* decreased, while the relative abundance of *Bacillus* increased and gradually became the dominant bacteria, reaching its highest relative abundance on the 20th day of fermentation (70.68%). This succession is remarkable because *Bacillus* are known for strong proteolytic and lipolytic activities, which may contribute to flavor development [34,35]. Furthermore, in addition to *Lactobacilli* and *Bacillus*, *Myroides*, *Staphylococcus* and *Enterococcus* were also the dominant bacteria, in F5, F10, and F20, respectively, accounting for 13.95%, 9.96% and 6.37%, respectively. These dominant bacteria in pigskin are similar to previous reports of traditional meat fermentation [33,36].

At the phylum level of fungi, Ascomycota (relative abundance ranging from 96.37% to 99.72%) was the dominant fungi throughout the entire fermentation of pigskin, and only two other fungi, Basidiomycota and Zygomycota, were detected (Figure 3C). As shown in Figure 3D, *Fusarium* (74.64%) and *Aspergillus* (15.43%) were the dominant fungi of F0, followed by *Phomopsis*, *Candida*, and *Cryptococcus* in terms of relative abundance. On the fifth day of fermentation, *Hanseniaspora* (49.98%), *Debaryomyces* (36.63%), and *Arthrinium* (11.90%) became the dominant fungi. However, on the 10th and 20th day of fermentation, the dominant fungi were only *Hanseniaspora* (20.57~24.57%) and *Debaryomyces* (69.77~72.63%). The rapid suppression of *Fusarium* and *Aspergillus* is a key safety advantage of bioaugmentation, as these genera include potential mycotoxin producers, while, the yeasts, e.g., *Hanseniaspora* and *Debaryomyces*, are known to contribute positively to flavor via ester and higher alcohol production, indicating a synergistic relationship with lactic acid bacteria [37,38,39,40,41].

### 2.3. Analysis of VFCs in the Fermentation of Pigskin

**Figure 4:**
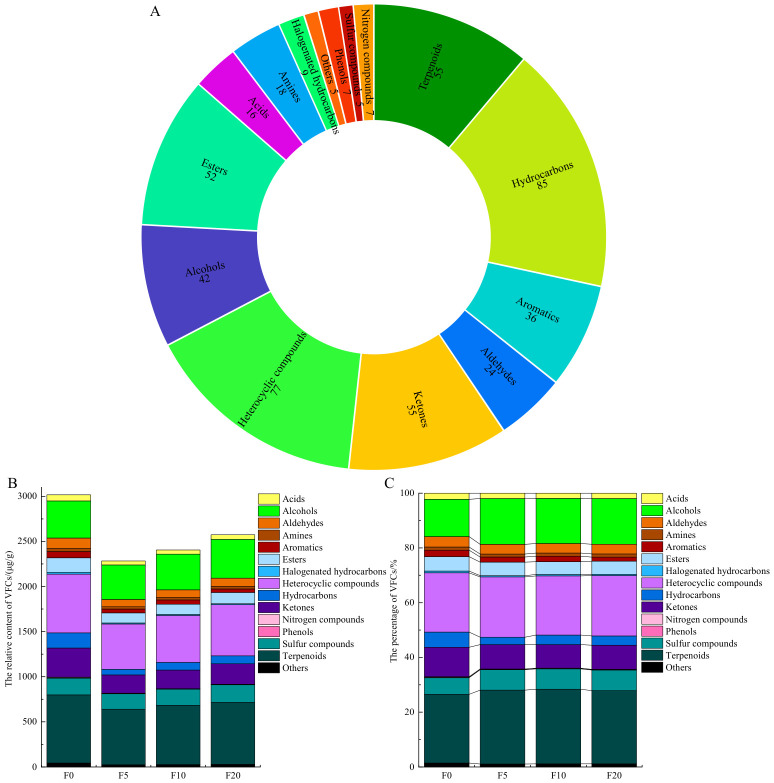
The analysis of volatile flavor compounds (VFCs) during fermentation of pigskin. The classes and numbers (the numbers in the figure) of VFCs (**A**), the relative content of VFCs (**B**), the percentage of VFCs (**C**).

To examine the influence of bioaugmented fermentation with *Ltb. sakei* YBZY-W5 on the flavor characteristics of fermented pigskin, this study used GC-MS to analyze the VFCs in the fermentation of pigskin. The results indicate that a total of 493 VFCs were identified during the fermentation of pigskin, which were classified into 15 classes (Figure 4A and Table S1); meanwhile, the major VFCs included hydrocarbons (85 VFCs), heterocyclic compounds (77 VFCs), terpenoids (55 VFCs) and ketones (55 VFCs), followed by esters (52 VFCs), alcohols (42 VFCs), aromatics (36 VFCs), aldehydes (24 VFCs), amines (18 VFCs), acids (16 VFCs), halogenated hydrocarbons (9 VFCs), phenols (7 VFCs), nitrogen compounds (7 VFCs), sulfur compounds (5 VFCs) and others (5 VFCs). As shown in Figure 4B, terpenoids (615.82~755.27 μg/g), heterocyclic compounds (505.30~652.43 μg/g), and alcohols (382.51~430.48 μg/g) were the classes of VFCs with the highest relative content in fermented pigskin, and indicating that these classes of VFCs constituted the primary chemical basis for aromatic characteristics and biological activity of fermented pigskin. Meanwhile, the relative content of these three classes of VFCs first decreased and then increased with the progress of fermentation, suggesting initial consumption or degradation followed by de novo synthesis. For instance, terpenoids are mainly derived from spices and can be biotransformed by microbial enzymes; heterocyclic compounds often arise from Maillard reactions and microbial metabolism, contributing roasted and nutty notes; alcohols are generated from amino acid catabolism by LAB [42,43]. Furthermore, VFCs with relatively high relative content also included ketones (203.06~324.23 μg/g), sulfur compounds (170.36~193.76 μg/g), esters (111.47~160.03 μg/g), etc. According to the analysis of the percentage of VFCs, six classes of VFCs including terpenoids (25.03~27.25%), heterocyclic compounds (21.62~22.06%), alcohols (13.61~16.75%), ketones (8.56~10.75%), sulfur compounds (6.10~7.53%), and esters (4.63~5.30%) were the main flavor compounds in fermented pigskin (Figure 4C).

**Figure 5:**
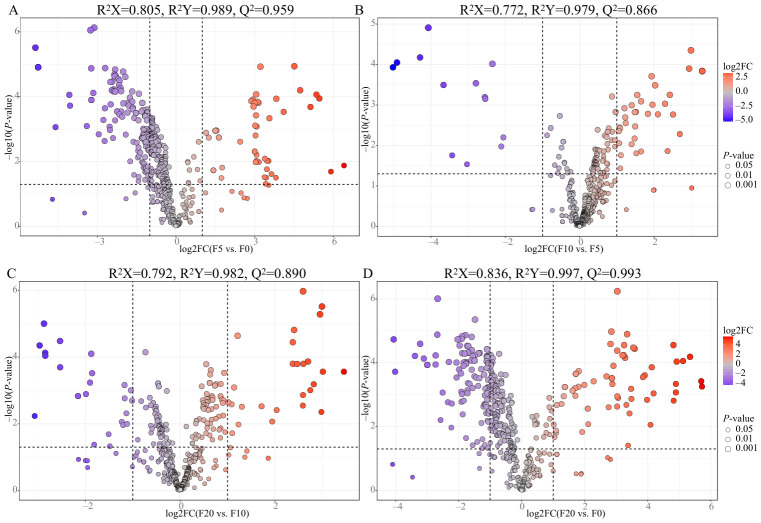
The differentially changed volatile flavor compounds (DCVFCs) between different fermented pigskins. A total of 185 DCVFCs between F5 and F0 (**A**), 48 DCVFCs between F10 and F5 (**B**), 50 DCVFCs between F20 and F10 (**C**), 175 DCVFCs between F20 and F0 (**D**). The horizontal dotted line represents *p* = 0.05, and the vertical dotted line represents FC = 0.5 or 2.

To gain an overview of the differentially changed volatile flavor compounds (DCVFCs), the analysis of VFCs in different fermented pigskins was performed using OPLS-DA (Figure 5). To validate the robustness of the OPLS-DA models, key parameters including R^2^X, R^2^Y and Q^2^ were evaluated. As shown in Figure 5, the obtained values (R^2^X > 0.75, R^2^Y > 0.95, Q^2^ > 0.85) indicated that the models not only fit the input data well but also exhibit strong discriminative ability among different fermentation time points (F0, F5, F10, F20). Notably, the high Q^2^ value (well above the commonly accepted threshold of 0.5) and its proximity to R^2^Y confirmed that the models possessed excellent predictive power and were not overfitted. These results provided a solid statistical basis for the reliable identification of DCVFCs under the applied screening criteria (*p* < 0.05, VIP > 1.0, and FC > 2.0 or <0.5).

The results showed that as fermentation proceeds, the relative content of 134 DCVFCs in F5 decreased significantly (*p* < 0.05, VIP > 1.0, and FC < 0.5) compared to F0 (Figure 5A and Table S2), including heterocyclic compounds (23 DCVFCs), hydrocarbons (22 DCVFCs), ketone (21 DCVFCs), terpenoids (19 DCVFCs), esters (14 DCVFCs), alcohols (6 DCVFCs), amines (6 DCVFCs), aromatics (6 DCVFCs), acids (5 DCVFCs), etc. Meanwhile, the relative content of 51 DCVFCs in F5/F0 increased significantly (*p* < 0.05, VIP > 1.0, and FC > 2.0), including hydrocarbons (11 DCVFCs), esters (9 DCVFCs), heterocyclic compounds (7 DCVFCs), aromatics (5 DCVFCs), alcohols (5 DCVFCs), aldehydes (4 DCVFCs), amines (3 DCVFCs), acids (3 DCVFCs), etc. The large number of early changes reflects the immediate metabolic impact of *Ltb. sakei* inoculation. Many terpenoids with green or woody flavor (β-myrcene, α-ionone) decreased, while esters with fruity flavor (octanoic acid, ethyl ester, hexanoic acid, ethyl ester) increased. This shift is desirable for consumer acceptance.

As shown in Figure 5B and Table S3, the relative content of 13 DCVFCs in F10 decreased significantly (*p* < 0.05, VIP > 1.0, and FC < 0.5) compared to F5, including ketones (3 DCVFCs), esters (2 DCVFCs), alcohols (2 DCVFCs), acids (2 DCVFCs), etc. Moreover, the relative content of 35 DCVFCs increased significantly (*p* < 0.05, VIP > 1.0, and FC > 2.0), including esters (6 DCVFCs), hydrocarbons (6 DCVFCs), amines (5 DCVFCs), ketones (4 DCVFCs), terpenoids (4 DCVFCs), heterocyclic compounds (3 DCVFCs), acids (2 DCVFCs), aromatics (2 DCVFCs), etc.

Additionally, in the F20 vs. F10 comparison, the relative content of 26 DCVFCs increased significantly (*p* < 0.05, VIP > 1.0, and FC > 2.0), including esters (5 DCVFCs), hydrocarbons (5 DCVFCs), ketones (4 DCVFCs), alcohols (3 DCVFCs), heterocyclic compounds (3 DCVFCs), acids (2 DCVFCs), etc. (Figure 5C and Table S4). Meanwhile, the relative content of 24 DCVFCs decreased significantly (*p* < 0.05, VIP > 1.0, and FC < 0.5), including heterocyclic compounds (7 DCVFCs), esters (3 DCVFCs), hydrocarbons (3 DCVFCs), alcohols (2 DCVFCs), etc. (Figure 5C).

Importantly, the relative content of 64 DCVFCs increased significantly (*p* < 0.05, VIP > 1.0, and FC > 2.0) in the F20 vs. F0 comparison, including esters (13 DCVFCs), hydrocarbons (11 DCVFCs), heterocyclic compounds (6 DCVFCs), aromatics (6 DCVFCs), acids (5 DCVFCs), alcohols (4 DCVFCs), ketones (4 DCVFCs), amines (4 DCVFCs), terpenoids (3 DCVFCs), aldehydes (3 DCVFCs), sulfur compounds (2 DCVFCs), etc. (Figure 5D and Table S5). Among them, the OAV of 8 DCVFCs were greater than 1, e.g., dimethyltrisulfide, cyclohexanone,5-methyl-2-(1-methylethyl)-, acetylpyrazine, hexanoic acid, ethyl ester, indole, (2R-cis)-5-methyl-2-(1-methylethyl)-cyclohexanone, octanoic acid, ethyl ester, pyrazine,trimethyl- (Table 1), indicating their key sensory contributions.

Meanwhile, the relative content of 111 DCVFCs in F20/F0 were decreased significantly (*p* < 0.05, VIP > 1.0, and FC < 0.5), including hydrocarbons (22 DCVFCs), ketones (18 DCVFCs), heterocyclic compounds (17 DCVFCs), terpenoids (16 DCVFCs), esters (8 DCVFCs), aromatics (7 DCVFCs), alcohols (6 DCVFCs), amines (4 DCVFCs), halogenated hydrocarbons (3 DCVFCs), nitrogen compounds (2 DCVFCs), phenols (2 DCVFCs), acids (2 DCVFCs), aldehydes (2 DCVFCs), etc. (Figure 5D and Table S5). Among them, the OAV of 13 DCVFCs were greater than 1, e.g., 1,3,8-p-menthatriene, trans-β-ocimene, 3,5-octadien-2-one,(E,E)-, β-myrcene, 5-hepten-2-one,6-methyl-, 2-octanone, α-ionone, β-pinene, phenylethyl alcohol, heptanoic acid,methyl ester, 3-octanol, dl-menthol, 3-phenylpropanol (Table 1), and most of them are related to green and woody odor.

**Figure 6:**
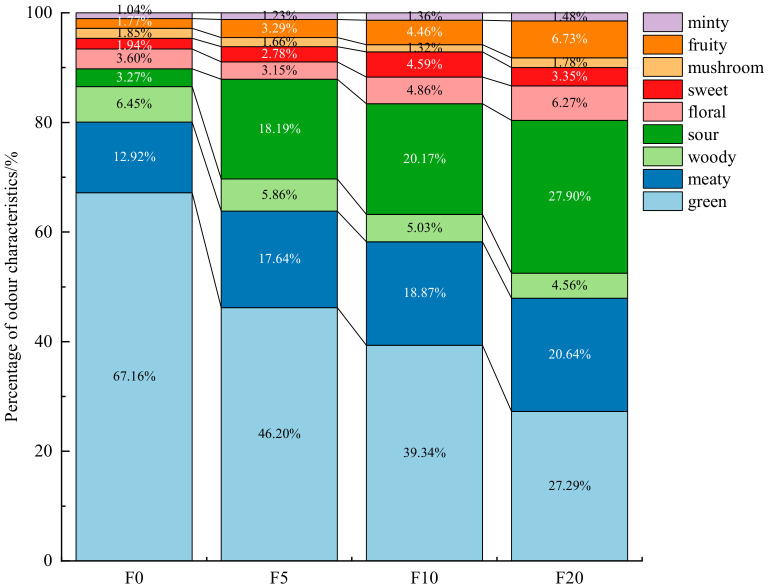
Percentage of odor characteristics during fermentation of pigskin.

To further analyze how the odor characteristics change from VFCs during the fermentation of pigskin, we introduced OAV analysis. The OAV was calculated as the ratio of the relative concentration of each VFCs to its odor threshold (Equation (1)). Only compounds with OAV > 1 are considered odor-active because their concentration exceeds the human olfactory detection limit. For each fermentation sample (F0, F5, F10, F20), we identified all VFCs with OAV > 1, grouped them by odor descriptor (e.g., green, meaty, woody, sour, floral, sweet, mushroom, fruity, minty), and summed the OAVs within each descriptor. By analyzing the VFCs with OAV greater than 1 in each fermentation sample, and summing up the OAV of VFCs with the same characteristics, the percentage of odor characteristics as shown in Figure 6 was obtained (only odor characteristics with a contribution greater than 1% were displayed). Although OAV analysis provides useful sensory references, the odor thresholds employed are primarily determined in water, which may not completely capture perceptual interactions in the complex, lipid-rich environment of fermented pigskin. In all fermented pigskin samples, the odor characteristics were composed of nine characteristics including green, meaty, woody, sour, floral, sweet, mushroom, fruity and minty. In the initial stage of fermentation, the odor characteristics of F0 were mainly green, meaty, woody, followed by sour, floral and sweet; as fermentation progresses, although the most important odor characteristics of pigskin was still green, sour, meaty, woody, floral, sweet, and fruity gradually became the main odor characteristics (Figure 6). This change is attributed to the microbial metabolism of *Ltb. sakei* and succeeding *Bacillus* and yeasts during fermentation of pigskin, which increased the relative content of VFCs (OAV > 1) with sour, meaty, floral, sweet, and fruity characteristics such as dimethyltrisulfide, octanoic acid, ethyl ester, hexanoic acid, ethyl ester, benzeneacetic acid, and geraniol, while decreasing the relative content of VFCs (OAV > 1) with green, woody and mushroom characteristics such as 5-hepten-2-one, 6-methyl-, β-pinene, 3-octanol, and 1,3,8-p-menthatriene.

### 2.4. Correlation Analysis Between Microorganisms and DCVFCs During the Fermentation of Pigskin

**Figure 7:**
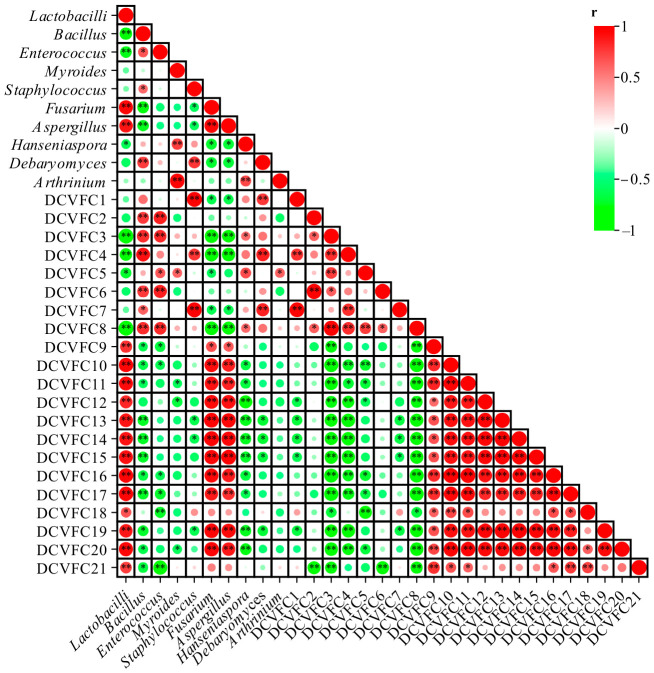
Pearson’s correlation analysis between differentially changed volatile flavor compounds (DCVFCs, odor activity value of DCVFCs > 1) and microorganisms. DCVFC1: dimethyltrisulfide, DCVFC2: cyclohexanone,5-methyl-2-(1-methylethyl)-, DCVFC3: acetylpyrazine, DCVFC4: hexanoic acid, ethyl ester, DCVFC5: indole, DCVFC6: (2R-cis)-5-methyl-2-(1-methylethyl)-cyclohexanone, DCVFC7: octanoic acid, ethyl ester, DCVFC8: pyrazine,trimethyl-, DCVFC9: 1,3,8-p-menthatriene, DCVFC10: trans-β-ocimene, DCVFC11: 3,5-octadien-2-one,(E,E)-, DCVFC12: β-myrcene, DCVFC13: 5-hepten-2-one,6-methyl-, DCVFC14: 2-octanone, DCVFC15: α-ionone, DCVFC16: β-pinene, DCVFC17: phenylethylalcohol, DCVFC18: heptanoic acid,methyl ester, DCVFC19: 3-octanol, DCVFC20: dl-menthol, DCVFC21: 3-phenylpropanol. *: *p* < 0.05; **: *p* < 0.01.

To elucidate the metabolic roles of key microorganisms, Pearson’s correlation analysis was performed between dominant microorganisms and DCVFCs with OAV > 1 (Figure 7 and Table S6). Based on the value of the correlation coefficient (r) and *p*-value, |r| > 0.6 and *p* < 0.05 indicated a strong correlation [44,45]. *Lactobacilli* was strongly positively correlated with *Fusarium*, *Aspergillus*, 1,3,8-p-menthatriene, trans-β-ocimene, 3,5-octadien-2-one,(E,E)-, β-myrcene, 5-hepten-2-one,6-methyl-, 2-octanone, α-ionone, β-pinene, phenylethyl alcohol, heptanoic acid,methyl ester, 3-octanol, dl-menthol, and 3-phenylpropanol (r > 0.6 and *p* < 0.05), while strongly negatively correlated with *Bacillus*, *Enterococcus*, *Hanseniaspora*, acetylpyrazine, hexanoic acid, ethyl ester, indole and pyrazine,trimethyl- (r < −0.6 and *p* < 0.05). This suggests that *Ltb. sakei* YBZY-W5, while dominating early, may stabilize spice-derived green and woody VFCs, but may not contribute to the desirable fruity and floral VFCs that appear later.

*Bacillus* was strongly positively correlated with *Enterococcus*, *Staphylococcus*, *Debaryomyces*, cyclohexanone,5-methyl-2-(1-methylethyl)-, acetylpyrazine, hexanoic acid, ethyl ester, (2R-cis)-5-methyl-2-(1-methylethyl)-cyclohexanone, octanoic acid, ethyl ester and pyrazine,trimethyl- (r > 0.6 and *p* < 0.05), and strongly negatively correlated with *Lactobacilli*, *Fusarium*, *Aspergillus*, 1,3,8-p-menthatriene, trans-β-ocimene, 3,5-octadien-2-one,(E,E)-, 5-hepten-2-one,6-methyl-, 2-octanone, α-ionone, β-pinene, phenylethyl alcohol, 3-octanol, dl-menthol and 3-phenylpropanol (r < −0.6 and *p* < 0.05). This indicates that *Bacillus*, which became dominant after day 10, may be key producers of desirable VFCs, likely via their strong esterase and amino acid metabolism activities. The positive correlation between *Bacillus* and *Debaryomyces* suggests synergistic interactions, possibly through cross-feeding of amino acids or vitamins.

*Myroides* was strongly positively correlated with *Hanseniaspora*, *Arthrinium* and indole (r > 0.6 and *p* < 0.05), while strongly negatively correlated with 3,5-octadien-2-one,(E,E)-, β-myrcene and dl-menthol (r < −0.6 and *p* < 0.05). *Hanseniaspora* was strongly positively correlated with *Myroides*, *Arthrinium*, acetylpyrazine, pyrazine,trimethyl- and indole (r > 0.6 and *p* < 0.05), while strongly negatively correlated with *Lactobacilli*, *Fusarium*, *Aspergillus*, trans-β-ocimene, 3,5-octadien-2-one,(E,E)-, β-myrcene, 5-hepten-2-one,6-methyl-, 2-octanone, α-ionone, β-pinene, phenylethyl alcohol, 3-octanol and dl-menthol (r < −0.6 and *p* < 0.05). Yeasts of *Hanseniaspora* are known for producing fruity esters and higher alcohols, consistent with their positive correlation with ethyl esters and pyrazines [46,47]. *Debaryomyces* was strongly positively correlated with *Bacillus*, *Staphylococcus*, dimethyltrisulfide, hexanoic acid, ethyl ester and octanoic acid (r > 0.6 and *p* < 0.05), and strongly negatively correlated with *Fusarium*, *Aspergillus*, 5-hepten-2-one,6-methyl-, 2-octanone, α-ionone and 3-octanol (r < −0.6 and *p* < 0.05). These results confirm that the fungal community, particularly *Debaryomyces*, plays an active role in flavor development, especially in generating sulfur-containing meaty notes [48,49].

### 2.5. Microbial Community Involvement and Functional Roles During Pigskin Fermentation

The bioaugmented fermentation of pigskin with *Ltb. sakei* YBZY-W5 drove a well-defined succession of both bacterial and fungal communities, each contributing distinct metabolic functions to the fermentation process. In the early stage (days 0–5), *Lactobacilli* (dominated by the inoculated *Ltb. sakei*) rapidly became the most abundant bacterial genus (up to 83.10% relative abundance). Its primary functions included rapid acidification via organic acid production (evidenced by a significant pH decrease, Figure 1B), inhibition of spoilage and potentially harmful microorganisms (e.g., *Fusarium* and *Aspergillus* were suppressed after day 5), and partial proteolysis that generated free amino acids as precursors for further flavor development. Concurrently, the fungal community shifted from *Fusarium* and *Aspergillus* (which may produce mycotoxins) to *Hanseniaspora* and *Debaryomyces*, indicating that the acidifying environment created by *Ltb. sakei* selectively favored salt-tolerant, fermentative yeasts while suppressing undesired molds.

During the mid-to-late fermentation (days 10–20), a notable ecological succession occurred: *Lactobacilli* gradually declined, while *Bacillus* became the dominant bacterial genus (reaching 70.68% on day 20). This transition was accompanied by the stable dominance of *Hanseniaspora* (20.57–24.57%) and *Debaryomyces* (69.77–72.63%) among fungi. Functionally, *Bacillus* are known for their strong proteolytic and lipolytic activities, which contribute to the generation of free fatty acids and peptides, precursors of many volatile compounds; *Hanseniaspora* and *Debaryomyces* are proficient in producing esterases and alcohol acetyltransferases, leading to the synthesis of fruity esters (e.g., ethyl hexanoate, ethyl octanoate) and higher alcohols [24,25]. The correlation analysis (Figure 7) confirmed that *Bacillus* and *Debaryomyces* were strongly positively correlated with key odor-active VFCs such as dimethyltrisulfide (meaty), acetylpyrazine (nutty/roasted), and several ethyl esters (fruity). In contrast, *Lactobacilli* showed strong positive correlations with green/woody terpenoids (β-myrcene, α-ionone, β-pinene) that declined during fermentation, indicating that the inoculated strain helped stabilize spice-derived notes initially but did not directly produce the most desirable fruity and floral aromas. To sum up, the microbial community functioned in a complementary, two-phase manner: *Ltb. sakei* acted as an initial “ecological engineer” by lowering pH and suppressing competitors, thereby creating permissive conditions for subsequent aroma-generating bacteria (*Bacillus*) and yeasts (*Hanseniaspora*, *Debaryomyces*). This cooperative microbial metabolism is the driving force behind the transformation of pigskin from a raw material with green/woody odors to a product rich in fruity, floral, sweet, and meaty notes. This two-phase metabolic cooperation is a hallmark of successful traditional fermentation (e.g., soy sauce, cheese) and is often difficult to achieve with a single starter culture [50,51]. While the Pearson’s correlation analysis provided valuable insights into potential associations between dominant microorganisms and key differentially changed volatile flavor compounds (DCVFCs), it is important to acknowledge the inherent limitations of correlation-based inference. Correlation does not imply causation, and the observed statistical relationships may arise from indirect interactions, shared ecological drivers, or co-occurrence patterns rather than direct metabolic activities. Therefore, although our results suggest hypotheses regarding the roles of *Lactobacilli*, *Bacillus*, *Hanseniaspora*, and *Debaryomyces* in flavor compound formation, these findings require further functional validation. Future studies should employ complementary approaches such as monoculture fermentation experiments, stable isotope tracing, in vitro enzyme activity assays, or genetic manipulation (e.g., gene knockout/complementation) to confirm the direct biosynthetic capabilities of specific microbial taxa. This will be essential to establish definitive mechanistic links between microbial metabolism and flavor development during pigskin fermentation. Additionally, this study only employed *Ltb. sakei* YBZY-W5 as a single strain for bioaugmentation, without setting up treatment groups that received mixed cultures (e.g., a combination of LAB, *Bacillus*, and yeasts). Therefore, it was not possible to evaluate the potential benefits of multi-species synergistic inoculation on flavor complexity, fermentation efficiency, or the stability of the microbial network. Meanwhile, the risks of competitive failure or functional decline of a single strain in a complex ecological niche could not be ruled out. Consequently, the optimal inoculation strategy still needs to be determined through comparative experiments.

Overall, *Ltb. sakei* YBZY-W5 does not directly contribute to the biosynthesis of the major desirable aroma compounds (such as ethyl esters and pyrazines), it plays a critical ecological role as an indirect facilitator. Its primary functions include rapid acidification (evidenced by decreasing pH), suppression of undesirable fungi (*Fusarium*, *Aspergillus*), and modulation of the fermentation environment, which collectively create favorable conditions for the subsequent establishment and metabolic activity of aroma-producing microorganisms, particularly *Bacillus*, *Hanseniaspora* and *Debaryomyces*. Consequently, the bioaugmentation strategy using *Ltb. sakei* YBZY-W5 primarily leverages its niche-constructing capacity rather than its direct flavor-forming metabolism. This refined conceptualization enhances the mechanistic understanding of microbial succession and flavor development in fermented pigskin.

## 3. Materials and Methods

### 3.1. Materials and Reagents

Fresh pigskin, rice, sugar, ginger powder, *Litsea cubeba* essential oil and other spices were all purchased from the local market in Yibin, Sichuan, China in November 2025. *Latilactobacillus sakei* YBZY-W5 was isolated and purified from the fermentation of pigskin, and it was identified by morphological analysis and phylogenetic analysis of 16S rRNA. The mixture of C_8_–C_20_ n-alkanes standard mix solution (40 mg/L each, in hexane) were purchased from Sigma-Aldrich (Shanghai, China), and a headspace solid-phase microextraction coupled with gas chromatography-mass spectrometry (HS-SPME-GC-MS) (8890-7000D) system, equipped with a DB-5MS capillary column (30 m × 0.25 mm × 0.25 µm), was purchased from Agilent Technologies (Santa Clara, CA, USA) for the analysis of volatile flavor compounds.

### 3.2. Sample Preparation

Fresh pigskin was dehaired and cut into 1 cm × 1 cm pieces. Ginger (5% of the pigskin weight) and five times the amount of purified water were added, and the mixture was boiled at 100 °C for 20 min. Then, the pigskin was removed and allowed to cool. Meanwhile, after removing impurities, the rice was dried at 150 °C until its moisture content is below 10%, and then crushed and sieved through a 40-mesh screen. Subsequently, the amounts of ingredients (rice flour 30% of pigskin weight, sucrose 5%, salt 2%, soy sauce 5%, ginger powder 2%, *Litsea cubeba* essential oil 1%) were added to the processed pigskin and thoroughly mixed to achieve a balanced final product with moderate saltiness, acidity, and desirable aroma, while ensuring sufficient carbohydrate supply for microbial growth. Mixtures with a mass of 8.5 kg were then transferred into 10 L ceramic jars that had been pre-rinsed with 75% ethanol, filling the jars to 90% of their capacity. The jar mouth was wiped clean with an alcohol-soaked cotton ball and sealed with two layers of plastic wrap. Fermentation was carried out under static conditions at the natural ambient range of 18~26 °C for 20 days to obtain the final fermented pigskin product. Three independent replicate experiments were performed simultaneously.

Samples were taken on days 0, 5, 10, and 20 of fermentation. At each sampling time point (0, 5, 10, 20 days), 60 g of sample was collected from each jar using a five-point sampling method (i.e., sampling from the center, four symmetrical peripheral points approximately 3 cm from the jar wall, all at a depth of 3 cm below the surface) under aseptic conditions. Any adhering material on the surface of the collected sample was removed and immediately divided into three portions for the determination of physicochemical parameters, microbial diversity, and VFCs, respectively, and stored at −80 °C until analysis. The collected samples were designated as F0, F5, F10, and F20, respectively. After sampling, the remaining pigskin in the jar was gently pressed down to remove air pockets, the mouth of the jar was wiped with 75% ethanol-soaked cotton, and the jar was resealed with two layers of plastic wrap to maintain anaerobic conditions and prevent contamination.

### 3.3. Measurement of Physicochemical Parameters

The content of moisture was determined as described previously [52], and the pH of the samples was determined using the calibrated pH meter (HI9025C, HANNA Instruments, Woonsocket, RI, USA). The content of nitrite was evaluated by the Spectro photometric method following accordance with Chinese national standard method (GB 5009.33-2016) [53]; according to this standard, the sample was deproteinized and defatted, after which nitrite was diazotized with sulfanilic acid under weak acid conditions and then coupled with N-(1-naphthyl)ethylenediamine dihydrochloride to form a purple-red azo dye, the absorbance of which was measured and quantified against an external standard curve. The content of total volatile basic nitrogen (TVB-N) was determined by a KDA-04A Kjeldahl apparatus (Zhejiang Lichen Instrument Technology Co., Ltd., Shaoxing, China) according to Chinese national standard method (GB 5009.228-2016) [54]. The principle of this method is that volatile basic nitrogen compounds, produced by enzymatic and microbial decomposition of proteins were distilled under alkaline conditions, absorbed by boric acid solution, and subsequently titrated with a standard acid solution to calculate the TVB-N content. Subsequently, the content of thiobarbituric acid reactive substance (TBARS) was measured as described previously [55]. This assay is based on the reaction between malondialdehyde, a secondary lipid peroxidation product, and thiobarbituric acid (TBA) under acidic and heating conditions to form a pink chromogen, whose absorbance is measured spectrophotometrically at 532 nm. All absorbance measurements for both nitrite and TBARS assays were performed using a UV-2600i spectrophotometer (Shimadzu, Kyoto, Japan).

### 3.4. Microbial Diversity Assessment

Majorbio Bio-Pharm Technology Co. Ltd. in Shanghai (China) performed the Illumina amplicon sequencing of microorganisms on the surfaces of pigskins. A total of 5 g of fermented pigskin, 15 mL of PBS buffer (4 °C) and 5 glass beads were added into a 50 mL centrifuge tube and shaken well for 5 min. Then centrifuge at 150× *g*, 4 °C for 5 min, and take the supernatant in the centrifuge tube. In the following step, add 5 mL of PBS buffer to the pellet and repeat the washing twice, centrifuge at 150× *g* for 5 min, and collect the supernatant. Centrifuge the collected supernatant at 10,000× *g*, 4 °C for 10 min, and collect the bacterial pellet. Meanwhile a FastPure Stool DNA Isolation Kit (Magnetic Beads) from Majorbio Bio-Pharm Technology Co., Ltd. (Shanghai, China) was employed to extract the total microbial genomic DNA. The 16S rRNA genes were sequenced via PCR amplification using the bacterial 338F primer and 806R reverse primer. The ITS1F and ITS2R primers were employed to amplify the fungal ITS region. The PCR amplification process included a 3 min denaturation at 95 °C, an additional 27 denaturation repetitions for 30 s each at 95 °C, 30 s annealing at 55 °C, a 45 s extension for at 72 °C, a 10 min extension at 72 °C, and termination at 4 °C. The second round was carried out in 13 cycles. Amplicons were purified with VAHTSTM DNA Clean Beads (Vazyme, Nanjing, China) and quantified using the Quant-iT PicoGreen dsDNA Assay Kit (Invitrogen, Carlsbad, CA, USA). After the individual quantification step, amplicons were pooled in equal amounts, and pairend sequencing (2 × 250 bp) was performed using a NextSeq 2000 system (Illumina, San Diego, CA, USA) [56].

The raw sequencing data were mainly processed by QIIME2 (Version 2026.1). Briefly, raw sequences were demultiplexed using the demux plugin (Version 3.0) followed by primers being removed with the cutadapt plugin (Version 2025.2). Sequences were then quality filtered, denoised, and merged, and chimera were removed using the DADA2 plugin (Version 1.34.0) [57]. Filter the reads if they were with the adapter sequence, the ratio of uncertain base was greater than 1%, and the content of low-quality base (Q ≤ 20) was greater than 50%. And filter out the reads whose length was still less than 150 bp after quality control. The amplicon sequence variants (ASVs) that were only found in one sample and singletons were further filtered. Finally, taxonomy was assigned to ASVs using the classify-sklearn naive Bayes taxonomy classifier in the featureclassifier plugin (Version 2025.2) against the Silva 138.2 (for bacteria) database and UNITE 8.0 (for fungi) database. Then, the raw sequencing information was entered into the Sequence Read Archive (SRA) database of National Center for Biotechnology Information (Accession Numbers: PRJNA1175882 and PRJNA1175888).

### 3.5. Determination of Volatile Flavor Compounds

A 0.5 g fermented pigskin was placed into a headspace vial together with 10 μL of an internal standard solution (100 μg/mL 2-octanol). The vial was equilibrated at 60 °C for 15 min under agitation at 250 rpm. Subsequently, 0.5 mL of headspace gas was withdrawn using a 2.5 mL airtight syringe that had been preheated to 80 °C. The volatile flavor compounds (VFCs) were extracted by exposing a DVB/CAR/PDMS (divinylbenzene/carboxen/polydimethylsiloxane) fiber (Supelco, Bellefonte, PA, USA) to the sample at 60 °C for 45 min, followed by desorption in the injection port of Agilent 8890-7000D gas chromatography–mass spectrometry system (Agilent Technologies, Santa Clara, CA, USA) at 250 °C for 3 min [58]. Separation was performed on a DB-5MS capillary column (30 m × 250 μm inner diameter, 0.25 μm film thickness; J&W Scientific, Folsom, CA, USA). The oven temperature program was as follows: initial hold at 50 °C for 1 min, then ramped to 310 °C at 10 °C/min, and finally held at 310 °C for 8 min. The injector, transfer line, and ion source temperatures were set to 280 °C, 270 °C, and 220 °C, respectively. Helium was used as a carrier gas at a constant flow rate of 0.8 mL/min. Electron ionization (EI) was carried out at −70 eV. Mass spectra were acquired in full-scan mode over the range of *m*/*z* 50–500 at a scan rate of 20 spectra per second, with a solvent delay of 6.12 min. Raw data processing, including peak extraction, baseline filtering and correction, alignment, deconvolution, identification, and area integration, was performed using Chroma TOF 4.3X software (LECO Corporation, Version 4.30) together with the LECO-Fiehn Rtx5 database. The identification of VFCs relied on both mass spectral matching and retention index (RI) comparison [59]. Only those VFCs showing a similarity greater than 80% against the NIST 1.6 and Wiley 6.0 databases were retained for further analysis [60]. Meanwhile, to enhance the confidence of qualitative assignments, the RI of each VFCs was calculated using a series of n-alkanes (C_8_–C_20_) [61]. According to the ratio of peak area between internal standard and VFCs, the relative concentration of VFCs were calculated. Additionally, Equation (1) was used to calculate the odor activity values (OAV) of VFCs:

(1) $${\mathrm{OAV}}_{i}\;=\frac{c_{i}}{T_{i}}$$

where *c*_i_ represents the relative concentration of VFCs and *T*_i_ denotes the odor threshold of VFCs in water. All the odor thresholds were obtained from the literature [62,63,64,65,66] and the following authoritative databases: FlavorDB (https://cosylab.iiitd.edu.in/flavordb/search, accessed on 7 April 2026), FooDB (http://www.foodb.ca, accessed on 7 April 2026) and The Good Scents Company Information System (http://www.thegoodscentscompany.com, accessed on 7 April 2026).

### 3.6. Statistical Analysis

Each group was analyzed in triplicate (*n* = 3); the results were expressed as mean values with standard deviations (SD). One-way ANOVA was employed to determine significant differences (*p* < 0.05) between samples. A Student–Newman–Keulsa comparison test was used to assess these differences (IBM SPSS, Version 20.0). Microbiota composition was visualized using ggplot2 (Version 2.2.1) in R project. Differentially changed VFCs (DCVFCs) were identified by orthogonal partial least squares discriminant analysis (OPLS-DA), with significance thresholds set at variable importance in projection (VIP), *p* < 0.05 and fold change (FC) > 2.0 or <0.5.

## 4. Conclusions

In summary, bioaugmented fermentation with *Ltb. sakei* YBZY-W5 significantly modulated the physicochemical parameters, microbial community succession, and volatile flavor profile of pigskin. The process ensured safety by suppressing potentially harmful fungi (*Fusarium*, *Aspergillus*) while promoting a desirable shift from green/woody to fruity/floral/sweet aroma characteristics. Key microbial players (e.g., *Lactobacilli*, *Bacillus*, *Hanseniaspora*, and *Debaryomyces*) exhibited distinct metabolic roles and synergistic interactions, with *Bacillus* and yeasts being major contributors to the formation of fruity esters, pyrazines, and sulfur-containing meaty compounds. This study demonstrates the feasibility of using *Ltb. sakei* YBZY-W5 as a starter culture for pigskin fermentation, providing a scientific basis for the high-value utilization of pigskin byproducts and contributing to the sustainable development of the pork industry. Future work should focus on optimizing fermentation conditions, validating the mechanisms through multi-omics approaches, and scaling up the process for industrial applications.

## Figures and Tables

**Table 1:** Description of odor and odor activity value (>1) of differentially changed volatile flavor compounds (DCVFCs) between the end point and starting point of fermented pigskin.

No.	Compounds	CAS	Description of Odor	Odor Threshold	F0	F5	F10	F20
DCVFC1	Dimethyltrisulfide	3658-80-8	cooked onion, savory, meaty	8.00 × 10^−6^	16,882.69 ± 518.16	122,595.89 ± 2709.02	124,209.39 ± 17,625.32	199,533.06 ± 20,597.28
DCVFC2	Cyclohexanone, 5-methyl-2-(1-methylethyl)-	10458-14-7	minty	4.70 × 10^−3^	56.02 ± 8.59	41.09 ± 5.70	80.31 ± 6.60	145.03 ± 12.93
DCVFC3	Acetylpyrazine	22047-25-2	nutty, corn, chip, bread, crust, chocolate, coffee	1.00 × 10^−2^	7.13 ± 0.22	63.07 ± 6.94	53.05 ± 3.09	85.41 ± 6.79
DCVFC4	Hexanoic acid, ethyl ester	123-66-0	apple, pear, fruity	5.00 × 10^−3^	3.83 ± 0.12	29.01 ± 2.97	46.16 ± 4.70	38.82 ± 2.29
DCVFC5	Indole	120-72-9	floral, moth, mothball, fecal	4.00 × 10^−2^	0.33 ± 0.01	19.99 ± 9.14	2.44 ± 0.34	17.25 ± 2.65
DCVFC6	(2R-cis)-5-methyl-2-(1-methylethyl)-Cyclohexanone	1196-31-2	minty	1.50 × 10^−1^	1.76 ± 0.27	1.29 ± 0.18	2.52 ± 0.21	4.54 ± 0.41
DCVFC7	Octanoic acid, ethyl ester	106-32-1	fruity, banana	4.00 × 10^−2^	0.12 ± 0.12	4.19 ± 0.53	12.21 ± 1.28	3.37 ± 0.24
DCVFC8	Pyrazine,trimethyl-	14667-55-1	nut skin, powdery, cocoa, baked, potato, roasted, musty	2.90 × 10^−1^	0.25 ± 0.01	2.19 ± 0.24	1.86 ± 0.09	2.96 ± 0.23
DCVFC9	1,3,8-p-Menthatriene	18368-95-1	turpentine, camphor, herbal, woody	1.50 × 10^−2^	1609.69 ± 150.78	988.12 ± 460.92	1109.63 ± 367.59	592.04 ± 13.47
DCVFC10	trans-β-Ocimene	3779-61-1	sweet, herbal	3.40 × 10^−2^	474.50 ± 25.61	228.88 ± 29.26	308.21 ± 30.66	227.76 ± 10.44
DCVFC11	3,5-Octadien-2-one,(E,E)-	30086-02-3	fruity, green, grassy	5.00 × 10^−4^	466.17 ± 24.54	195.78 ± 55.16	290.28 ± 31.52	215.27 ± 11.75
DCVFC12	β-Myrcene	123-35-3	musty, balsamic, spice	1.50 × 10^−2^	492.98 ± 22.03	12.04 ± 2.04	96.92 ± 72.46	164.41 ± 5.62
DCVFC13	5-Hepten-2-one, 6-methyl-	110-93-0	herbal, green, citrus, musty, lemon grass	5.00 × 10^−2^	572.72 ± 38.28	106.26 ± 10.70	111.05 ± 11.38	163.29 ± 6.28
DCVFC14	2-Octanone	111-13-7	weedy, natural, woody, herbal	6.00 × 10^−2^	105.59 ± 5.03	21.10 ± 2.97	22.37 ± 1.43	30.63 ± 0.71
DCVFC15	α-Ionone	127-41-3	sweet, woody, floral, violet, orris, tropical, fruity	3.78 × 10^−3^	74.52 ± 13.83	9.65 ± 4.07	13.00 ± 1.30	14.72 ± 1.76
DCVFC16	β-Pinene	127-91-3	woody, resinous, pine, hay, green	1.40 × 10^−1^	27.03 ± 1.13	12.59 ± 1.65	16.87 ± 1.71	12.63 ± 0.51
DCVFC17	Phenylethyl alcohol	60-12-8	fruity, rose, sweet, apple	1.40 × 10^−1^	35.53 ± 4.53	16.06 ± 3.08	20.05 ± 4.84	10.79 ± 0.80
DCVFC18	Heptanoic acid,methyl ester	106-73-0	sweet, fruity, green, waxy, floral, berry	4.00 × 10^−3^	18.84 ± 1.63	5.40 ± 0.66	22.02 ± 2.41	3.80 ± 0.28
DCVFC19	3-Octanol	589-98-0	mushroom, citrus, woody, spicy, minty	7.80 × 10^−2^	6.71 ± 0.75	1.41 ± 0.17	1.54 ± 0.14	2.13 ± 0.03
DCVFC20	dl-Menthol	89-78-1	cool, woody	1.30 × 10^−1^	2.62 ± 0.22	0.73 ± 0.18	1.22 ± 0.19	0.92 ± 0.10
DCVFC21	3-Phenylpropanol	122-97-4	sweet, spicy, cinnamyl, mignonette, hyacinth	4.20 × 10^−1^	2.22 ± 0.21	1.75 ± 0.14	1.94 ± 0.13	0.33 ± 0.03

Note: the description of odor were obtained from authoritative databases: FlavorDB (https://cosylab.iiitd.edu.in/flavordb/search, accessed on 7 April 2026), FooDB (http://www.foodb.ca, accessed on 7 April 2026) and The Good Scents Company Information System (http://www.thegoodscentscompany.com, accessed on 7 April 2026).

## Data Availability

The original contributions presented in this study are included in the article/Supplementary Materials. Further inquiries can be directed to the corresponding authors.

## Supplementary Materials

The following supporting information can be downloaded at: https://www.mdpi.com/article/10.3390/molecules31111889/s1, Table S1: The relative content of volatile flavor compounds (μg/g) during fermentation of pigskin. Table S2: The differentially changed volatile flavor compounds (DCVFCs) between F5 and F0. Table S3: The differentially changed volatile flavor compounds (DCVFCs) between F10 and F5. Table S4: The differentially changed volatile flavor compounds (DCVFCs) between F20 and F10. Table S5: The differentially changed volatile flavor compounds (DCVFCs) between F20 and F0. Table S6: The r-value of Pearson’s correlation analysis between differentially changed volatile flavor compounds and microorganisms.

## Abbreviations

The following abbreviations are used in this manuscript:

TVB-N	Total volatile basic nitrogen
TBARS	Thiobarbituric acid reactive substances
VFCs	Volatile flavor compounds
DCVFCs	Differentially changed volatile flavor compounds
OPLS-DA	Orthogonal partial least squares discriminant analysis
OAV	Odor activity value
