# Supplementary material for: Interaction and Flavor Metabolic Function of Microbiota During Fermentation of Pigskin Through Bioaugmentation with Latilactobacillus sakei
Source: Molecules. 2026 Jun 1;31(11):1889. doi: 10.3390/molecules31111889 (PMC13258469; doi:10.3390/molecules31111889)
Supplement: Supplementary file 1 [file molecules-31-01889-s001.zip › Table S1.pdf]

Table S1. The relative content of volatile flavor compounds (μg/g) during fermentation of pigskin.

No.	Compounds	Class	CAS	F0_1	F0_2	F0_3	F5_1	F5_2	F5_3	F10_1	F10_2	F10_3	F20_1	F20_2	F20_3
1	(+)-Dihydrocarvone	Terpenoids	5524-05-0	2.11E+00	2.27E+00	2.09E+00	1.50E+00	1.45E+00	1.14E+00	1.24E+00	1.54E+00	1.64E+00	1.35E+00	1.32E+00	1.47E+00
2	(1-methylethylidene)-Cyclohexane	Hydrocarbons	5749-72-4	1.53E-01	1.69E-01	1.67E-01	4.79E-02	2.99E-02	3.71E-02	1.41E-01	1.59E-01	1.39E-01	3.78E-02	4.20E-02	3.97E-02
3	(2-chloro-1-methylethyl)-Benzene	Aromatics	824-47-5	5.72E-01	6.53E-01	6.25E-01	4.16E-01	3.47E-01	2.53E-01	3.31E-01	3.87E-01	4.49E-01	3.43E-01	3.24E-01	3.65E-01
4	(2-methyl-1-propenyl)-Cyclohexane	Hydrocarbons	89656-98-4	2.32E-01	2.66E-01	2.48E-01	1.74E-01	1.34E-01	1.27E-01	1.71E-01	1.90E-01	2.04E-01	1.51E-01	1.62E-01	1.77E-01
5	(2E)-6Acetoxy-2-methylhexenal	Aldehydes	1000426-95-4	1.93E-01	1.78E-01	1.81E-01	1.48E-01	1.27E-01	1.05E-01	1.18E-01	1.39E-01	1.27E-01	1.19E-01	1.34E-01	1.55E-01
6	(2R-cis)-5-methyl-2-(1-methylethyl)-Cyclohexanone	Ketones	1196-31-2	3.05E-01	2.61E-01	2.24E-01	2.24E-01	1.83E-01	1.73E-01	3.42E-01	3.98E-01	3.93E-01	6.22E-01	6.80E-01	7.43E-01
7	(3R,6S)-2,2,6-Trimethyl-6-vinyltetrahydro-2H-pyran-3-ol	Heterocyclic compounds	39028-58-5	2.32E+00	2.62E+01	2.65E+01	2.24E+01	1.79E+01	1.67E+01	1.91E+01	2.15E+01	2.02E+01	1.91E+01	2.17E+01	2.47E+01
8	(4-Methylphenyl) methanol, neopentyl ether	Alcohols	1000374-65-9	1.37E-01	1.34E-01	1.16E-01	1.16E-01	6.54E-02	6.31E-02	9.24E-02	9.67E-02	8.75E-02	8.02E-02	9.45E-02	9.56E-02
9	(E)-2,2-dimethyl-3-Decene	Hydrocarbons	55499-02-0	5.39E+01	5.90E+01	5.94E+01	3.80E+01	3.39E+01	2.80E+01	3.36E+01	4.13E+01	4.43E+01	3.87E+01	3.60E+01	4.18E+01
10	(E)-2-Octenal	Aldehydes	2548-87-0	1.82E-01	2.19E-01	2.03E-01	1.89E-01	1.66E-01	1.60E-01	1.51E-01	1.25E-01	1.29E-01	1.78E-01	1.89E-01	2.05E-01
11	(E)-8-methyl-5-Undecene	Hydrocarbons	39546-85-5	3.56E+00	4.04E+00	4.01E+00	2.28E+00	1.40E+00	1.47E+00	1.83E+00	2.28E+00	2.54E+00	1.72E+00	1.54E+00	1.81E+00
12	(E)-Hex-3-enyl (E)-2-methylbut-2-enoate	Esters	120603-00-1	4.07E+01	3.64E+01	3.64E+01	3.56E+01	2.47E+01	2.41E+01	2.62E+01	2.94E+01	2.65E+01	2.72E+01	3.08E+01	3.52E+01
13	(R)-4-methyl-1-(1-methylethyl)-Cyclohexene	Hydrocarbons	619-52-3	1.07E-02	4.50E-01	1.13E-02	1.19E-01	1.07E-01	9.94E-02	1.01E-01	1.02E-01	6.69E-02	1.21E-01	1.25E-01	1.10E-01
14	(R)-5,7-dimethyl-1,6-Octadiene	Terpenoids	85006-04-8	2.66E-01	2.93E-01	2.82E-01	1.69E-01	1.44E-01	1.34E-01	1.98E-01	2.28E-01	1.90E-01	1.42E-01	1.53E-01	1.47E-01
15	(S)-(+)-6-Methyl-1-octanol	Alcohols	110453-78-6	5.97E-01	6.99E-01	6.06E-01	5.49E-01	4.19E-01	3.71E-01	4.06E-01	4.34E-01	4.17E-01	6.65E-01	6.19E-01	7.37E-01

16	(S)-2-Chloro-3-methylbutyric acid	Acids	26782-74-1	1.59E-01	1.80E-01	1.74E-01	1.43E-01	1.23E-01	1.21E-01	1.63E-01	1.90E-01	1.59E-01	1.22E-01	1.30E-01	1.36E-01
17	(Z)-2-Decene	Hydrocarbons	20348-51-0	2.11E+00	2.58E+00	2.41E+00	1.37E+00	1.20E+00	1.12E+00	1.18E+00	1.45E+00	1.29E+00	1.20E+00	1.22E+00	1.24E+00
18	(Z)-2-Octen-1-ol	Alcohols	26001-58-1	5.50E+00	6.62E+00	6.16E+00	5.28E+00	4.52E+00	4.28E+00	4.30E+00	3.99E+00	4.08E+00	4.93E+00	5.14E+00	5.63E+00
19	α-Calacorene	Terpenoids	21391-99-1	3.27E-02	3.14E-02	2.23E-02	2.13E-02	1.22E-02	1.20E-02	2.31E-02	2.38E-02	2.08E-02	1.75E-02	2.36E-02	2.16E-02
20	α-Cubebene	Terpenoids	17699-14-8	1.56E-01	1.38E-01	1.17E-01	2.43E-02	1.80E-02	1.80E-02	3.67E-02	3.40E-02	3.29E-02	3.23E-02	3.83E-02	4.01E-02
21	α-Farnesene	Terpenoids	502-61-4	6.12E-01	5.72E-01	4.34E-01	2.81E-01	1.58E-01	1.53E-01	2.69E-01	2.93E-01	2.44E-01	2.34E-01	2.61E-01	2.69E-01
22	α-Ionone	Terpenoids	127-41-3	3.29E-01	2.90E-01	2.26E-01	5.43E-02	2.75E-02	2.77E-02	4.42E-02	4.93E-02	5.40E-02	4.79E-02	5.98E-02	5.91E-02
23	α-Methylstyrene	Aromatics	98-83-9	8.53E-03	9.16E-03	8.72E-03	4.29E-03	2.73E-03	2.77E-03	4.83E-03	6.08E-03	5.28E-03	3.27E-03	3.32E-03	3.94E-03
24	α-Muurolene	Terpenoids	10208-80-7	9.45E-02	9.08E-02	7.04E-02	4.27E-02	2.65E-02	2.46E-02	4.36E-02	4.64E-02	3.74E-02	3.57E-02	4.04E-02	4.09E-02
25	α-Phellandrene 1	Terpenoids	99-83-2	3.60E+00	3.90E+00	3.85E+00	2.03E+00	1.67E+00	1.59E+00	2.26E+00	2.63E+00	2.19E+00	1.69E+00	1.80E+00	1.82E+00
26	α-Pinene	Terpenoids	80-56-8	8.39E-01	9.40E-01	8.94E-01	7.26E-01	6.24E-01	6.10E-01	8.42E-01	9.65E-01	8.09E-01	6.43E-01	6.74E-01	6.96E-01
27	α-Terpineol	Terpenoids	98-55-5	6.96E+00	7.41E+00	6.54E+00	3.54E+00	2.98E+00	2.69E+00	2.63E+00	3.25E+00	3.35E+00	3.48E+00	3.48E+00	3.63E+00
28	α-Terpinyl acetate	Esters	80-26-2	2.33E-02	2.18E-02	1.71E-02	1.81E-02	9.25E-03	9.58E-03	1.38E-02	9.95E-03	1.08E-02	9.88E-03	1.16E-02	1.08E-02
29	β-Myrcene	Terpenoids	123-35-3	7.23E+00	7.78E+00	7.18E+00	2.11E-01	1.80E-01	1.50E-01	2.17E-01	2.25E+00	1.89E+00	2.38E+00	2.46E+00	2.55E+00
30	β-Ocimene	Terpenoids	13877-91-3	5.42E+00	5.97E+00	5.81E+00	3.10E+00	2.54E+00	2.45E+00	3.46E+00	3.99E+00	3.34E+00	2.58E+00	2.76E+00	2.80E+00
31	β-Pinene	Terpenoids	127-91-3	3.60E+00	3.90E+00	3.85E+00	2.03E+00	1.67E+00	1.59E+00	2.26E+00	2.63E+00	2.19E+00	1.69E+00	1.80E+00	1.82E+00
32	δ-Dodecalactone	Esters	713-95-1	5.89E-03	1.12E-02	1.00E-02	9.53E-03	5.04E-03	8.69E-03	9.09E-03	1.08E-02	5.32E-03	6.11E-03	8.70E-03	6.01E-03
33	δ-Nonalactone	Esters	3301-94-8	1.04E-03	1.04E-03	1.09E-03	1.31E-02	7.73E-03	7.46E-03	9.83E-03	8.59E-03	7.67E-03	9.28E-03	1.22E-02	1.39E-02
34	γ-Muurolene	Terpenoids	30021-74-0	5.56E-02	4.33E-02	3.50E-02	4.53E-02	1.52E-02	1.26E-02	1.53E-01	1.40E-01	8.27E-02	3.79E-02	4.43E-02	4.97E-02
35	1,11-Dodecadiyne	Hydrocarbons	20521-44-2	1.10E+00	1.41E+00	1.13E+00	1.32E+00	1.10E+00	1.13E+00	1.18E+00	1.39E+00	1.29E+00	1.06E+00	1.10E+00	1.18E+00
36	1,2,3-trimethoxy-Propane	Hydrocarbons	20637-49-4	4.37E-02	4.94E-02	4.64E-02	1.85E-02	1.24E-02	1.37E-02	2.13E-02	1.52E-02	1.61E-03	8.08E-03	9.96E-03	1.90E-02
37	1,2,3-trimethyl-Cyclopentene	Hydrocarbons	473-91-6	4.82E-03	4.82E-03	5.08E-03	3.95E-02	4.32E-02	3.47E-02	3.64E-02	3.55E-02	3.56E-02	4.37E-02	3.79E-02	3.87E-02

38	1,2,4,5-Tetrazin-3-Amine	Amines	79329-74-1	3.19E-02	3.28E-02	2.50E-02	2.13E-02	1.46E-02	1.85E-02	2.14E-02	3.83E-02	3.03E-02	2.08E-02	1.82E-02	2.02E-02
39	1,2,5-Trimethylpyrrole	Heterocyclic compounds	930-87-0	2.59E-02	3.07E-02	2.81E-02	2.51E-02	2.19E-02	2.07E-02	2.50E-02	2.30E-02	2.05E-02	2.24E-02	2.41E-02	2.41E-02
40	1,3,5,7-Cyclooctatetraene	Esters	629-20-9	2.62E-02	2.91E-02	2.69E-02	1.30E-02	1.06E-02	9.37E-03	1.24E-02	1.21E-02	9.78E-03	1.19E-02	1.38E-02	1.26E-02
41	1,3,6-Octatriene, 3,7-dimethyl-, (Z)-	Terpenoids	3338-55-4	1.52E+01	1.69E+01	1.64E+01	8.92E+00	7.36E+00	7.07E+00	1.01E+01	1.17E+01	9.71E+00	7.33E+00	7.94E+00	7.96E+00
42	1,3,8-p-Menthatriene	Terpenoids	18368-95-1	2.25E+01	2.67E+01	2.33E+01	2.08E+01	7.27E+00	1.64E+01	1.77E+01	2.15E+01	1.07E+01	8.91E+00	9.07E+00	8.67E+00
43	1,3-Benzodioxole	Heterocyclic compounds	274-09-9	5.35E+00	6.49E+00	6.06E+00	3.05E+00	2.65E+00	2.47E+00	2.80E+00	3.48E+00	3.09E+00	2.70E+00	2.72E+00	2.79E+00
44	1,3-Cyclohexadiene-1-carboxaldehyde, 2,6,6-trimethyl-	Terpenoids	116-26-7	9.49E-01	1.25E+00	9.99E-01	1.07E+00	9.10E-01	8.32E-01	8.47E-01	1.14E+00	1.07E+00	7.67E-01	8.90E-01	8.72E-01
45	1,3-Hexadiene, 3-ethyl-2-methyl-	Hydrocarbons	61142-36-7	7.61E-03	5.82E-03	8.82E-03	7.14E-03	4.24E-03	6.59E-04	7.38E-03	6.61E-04	1.03E-02	5.76E-04	6.68E-04	3.49E-03
46	1,3-Propanediamine, N-(1-methylethyl)-	Amines	3360-16-5	4.84E-01	5.19E-01	4.65E-01	1.75E-01	1.51E-01	1.35E-01	1.90E-01	2.07E-01	1.64E-01	2.85E-01	3.32E-01	3.34E-01
47	1,3-bis(1-methylethyl)-1,3-Cyclopentadiene	Hydrocarbons	123278-27-3	1.46E-01	1.38E-01	1.32E-01	1.02E-01	8.71E-02	7.48E-02	9.02E-02	9.40E-02	8.96E-02	8.49E-02	9.02E-02	9.08E-02
48	1,4-ButanediAmine	Amines	110-60-1	4.09E-02	4.48E-02	4.00E-02	1.47E-02	1.20E-02	1.04E-02	2.86E-02	1.45E-02	1.40E-02	1.45E-02	1.57E-02	1.45E-02
49	1,5,5-Trimethyl-6-methylene-cyclohexene	Hydrocarbons	514-95-4	2.20E-01	2.84E-01	2.38E-01	1.09E-01	1.07E-01	8.83E-02	1.20E-01	1.66E-01	8.83E-02	1.05E-01	1.12E-01	1.11E-01
50	1,5,5-trimethyl-3-methylene-Cyclohexene	Hydrocarbons	16609-28-2	2.20E-01	2.84E-01	2.38E-01	1.09E-01	1.07E-01	8.83E-02	1.20E-01	1.66E-01	8.83E-02	1.05E-01	1.12E-01	1.11E-01
51	1,6-Octadiene, 3,7-dimethyl-	Terpenoids	2436-90-0	4.97E-02	5.33E-02	5.48E-02	4.95E-02	4.35E-02	4.17E-02	5.70E-02	6.50E-02	5.27E-02	4.41E-02	4.48E-02	4.62E-02
52	1,6-dichloro-1,5-Cyclooctadiene	Halogenated hydrocarbons	29480-42-0	9.74E-02	8.96E-02	9.04E-02	7.36E-02	5.91E-02	5.69E-02	6.41E-02	7.02E-02	6.73E-02	6.24E-02	7.19E-02	8.33E-02
53	1,7-Diazabicyclo[2.2.0]heptane	Heterocyclic compounds	279-42-5	1.54E-02	1.75E-02	1.74E-02	1.65E-02	1.36E-02	1.46E-02	1.76E-02	1.92E-02	1.67E-02	1.30E-02	1.38E-02	1.20E-02
54	1-(1-cyclohexen-1-yl)-Ethanone	Ketones	932-66-1	2.98E+00	3.68E+00	3.38E+00	1.58E+00	1.39E+00	1.26E+00	1.53E+00	1.93E+00	1.71E+00	1.29E+00	1.27E+00	1.30E+00

55	1-(1-methylethyl)-2-nonyl-Cyclopropane	Hydrocarbons	41977-39-3	9.74E-03	1.09E-02	8.99E-03	7.50E-03	4.11E-03	4.09E-03	8.39E-03	8.59E-03	7.80E-03	9.46E-03	1.13E-02	1.05E-02
56	1-(2-methyl-2-cyclopenten-1-yl)-Ethanone	Ketones	1767-84-6	1.59E-01	1.75E-01	1.75E-01	4.79E-02	2.99E-02	3.71E-02	1.74E-01	2.20E-01	1.69E-01	5.99E-02	4.20E-02	3.97E-02
57	1-(3-butyloxiranyl)-Ethanone	Ketones	17257-80-6	4.02E-02	5.13E-02	4.45E-02	9.14E-02	7.73E-02	7.91E-02	6.24E-02	8.04E-02	6.84E-02	5.16E-02	5.76E-02	5.67E-02
58	1-(4-methylphenyl)-Ethanone	Ketones	122-00-9	1.48E+00	1.71E+00	1.64E+00	1.02E+00	8.06E-01	7.04E-01	8.35E-01	1.04E+00	1.16E+00	9.03E-01	8.61E-01	9.70E-01
59	1-Cyclohexene-1-carboxAldehyde, 4-(1-methylethenyl)-	Aldehydes	2111-75-3	8.66E-01	1.13E+00	8.90E-01	8.53E-01	7.11E-01	7.30E-01	7.62E-01	9.29E-01	8.46E-01	7.01E-01	7.09E-01	7.64E-01
60	1-Decanol	Alcohols	112-30-1	7.15E+01	6.49E+01	6.51E+01	6.04E+01	4.22E+01	4.15E+01	4.51E+01	5.10E+01	4.61E+01	4.66E+01	5.32E+01	6.08E+01
61	1-Decen-3-one	Ketones	56606-79-2	4.09E+00	4.62E+00	4.58E+00	2.59E+00	2.12E+00	1.86E+00	2.23E+00	2.80E+00	2.85E+00	2.35E+00	2.13E+00	3.06E+00
62	1-Decene, 8-methyl-	Hydrocarbons	61142-79-8	1.38E+00	1.59E+00	1.53E+00	8.92E-01	7.74E-01	7.26E-01	7.65E-01	9.57E-01	8.44E-01	7.94E-01	8.00E-01	7.92E-01
63	1-Dodecanol	Alcohols	112-53-8	2.68E-02	2.70E-02	2.04E-02	1.78E-02	1.19E-02	1.16E-02	1.62E-02	1.64E-02	1.33E-02	1.85E-02	1.63E-02	1.60E-02
64	1-Dodecen-1-ol, acetate	Esters	56438-08-5	4.13E-03	4.80E-03	2.95E-03	3.18E-04	2.63E-04	3.46E-03	1.54E-03	1.98E-03	2.66E-04	1.99E-03	7.27E-03	4.49E-03
65	1-Heptanol	Alcohols	111-70-6	8.38E-03	1.04E-02	1.02E-02	1.59E-03	1.31E-03	1.53E-03	1.13E-02	1.07E-02	6.66E-03	7.16E-03	8.34E-03	8.48E-03
66	1-Hexanol, 2-ethyl-	Alcohols	104-76-7	7.89E-01	9.01E-01	8.64E-01	5.57E-01	4.93E-01	4.42E-01	4.41E-01	5.51E-01	4.71E-01	4.53E-01	4.46E-01	4.34E-01
67	1-Iodoundecane	Hydrocarbons	4282-44-4	2.54E-03	2.65E-03	1.92E-03	1.12E-02	6.63E-03	5.76E-03	1.26E-02	9.33E-03	8.69E-03	1.55E-02	2.14E-02	2.23E-02
68	1-Methyl-2-n-hexylbenzene	Aromatics	1595-10-4	5.98E-01	5.64E-01	4.69E-01	5.26E-01	3.01E-01	2.94E-01	4.08E-01	4.18E-01	3.75E-01	3.37E-01	4.00E-01	3.93E-01
69	1-Methyl-3-formylindole	Heterocyclic compounds	19012-03-4	1.25E-04	1.26E-04	1.32E-04	2.20E-03	7.74E-04	2.40E-03	4.92E-03	5.18E-03	4.02E-03	1.75E-03	1.73E-03	3.02E-03
70	1-Methylimidazole-5-carboxaldehyde	Heterocyclic compounds	39021-62-0	2.21E-01	2.42E-01	2.24E-01	5.68E-02	6.78E-02	5.57E-02	6.16E-02	6.50E-02	5.15E-02	6.24E-02	6.33E-02	5.93E-02
71	1-Octanol	Alcohols	111-87-5	1.18E-02	1.38E-02	1.37E-02	1.63E-02	1.20E-02	1.42E-02	1.08E-02	1.07E-02	1.27E-02	1.64E-02	1.24E-02	1.70E-02
72	1-Octen-3-one	Ketones	4312-99-6	4.39E-02	4.92E-02	5.00E-02	4.51E-02	3.90E-02	3.98E-02	4.95E-02	6.04E-02	4.95E-02	3.73E-02	3.99E-02	4.02E-02
73	1-Pentadecene	Hydrocarbons	13360-61-7	2.82E-03	6.19E-03	3.20E-03	7.87E-03	3.76E-03	4.01E-03	8.31E-03	9.87E-03	6.42E-03	7.40E-03	8.94E-03	8.85E-03

74	1-Phenyl-1-butene	Aromatics	824-90-8	1.04E+01	1.10E+01	9.51E+00	7.00E+00	5.71E+00	6.37E+00	7.58E+00	7.06E+00	7.35E+00	5.24E+00	5.10E+00	5.58E+00
75	1-Piperidineethanol	Heterocyclic compounds	3040-44-6	4.26E-01	4.78E-01	4.55E-01	3.32E-01	3.04E-01	2.69E-01	3.00E-01	3.49E-01	3.78E-01	2.98E-01	2.79E-01	3.08E-01
76	1-Tetradecene	Hydrocarbons	1120-36-1	1.02E-01	9.17E-02	7.59E-02	5.81E-02	3.11E-02	2.52E-02	4.94E-02	4.99E-02	4.83E-02	4.36E-02	5.08E-02	5.54E-02
77	1-Tetrazol-2-ylethanone	Heterocyclic compounds	51410-11-8	1.83E+01	1.94E+01	1.54E+01	4.47E+00	3.69E+00	3.75E+00	4.02E+00	4.78E+00	3.97E+00	5.82E+00	6.00E+00	6.22E+00
78	1-chloro-Dodecane	Halogenated hydrocarbons	112-52-7	5.78E-01	4.82E-01	3.78E-01	9.68E-02	6.32E-02	5.82E-02	1.16E-01	1.24E-01	9.86E-02	1.28E-01	1.11E-01	1.54E-01
79	1-chloro-Heptane	Halogenated hydrocarbons	629-06-1	3.13E+00	3.49E+00	3.32E+00	2.01E+00	1.68E+00	1.62E+00	2.37E+00	2.60E+00	2.21E+00	1.61E+00	1.73E+00	1.71E+00
80	1-chloro-Nonane	Halogenated hydrocarbons	2473-01-0	1.14E+01	1.21E+01	1.24E+01	7.92E+00	7.35E+00	5.66E+00	6.54E+00	7.99E+00	8.73E+00	7.54E+00	7.27E+00	8.17E+00
81	1-ethenyl-3-ethyl-Benzene	Aromatics	7525-62-4	6.22E+00	8.39E+00	7.27E+00	5.37E+00	4.35E+00	4.03E+00	5.83E+00	5.41E+00	5.64E+00	4.05E+00	3.97E+00	4.33E+00
82	1-ethyl-4-methyl-Benzene	Aromatics	622-96-8	8.24E-01	1.59E+00	9.77E-01	6.54E-01	1.76E+00	4.98E-01	5.74E-01	6.45E-01	8.73E-01	5.81E-01	6.09E-01	6.22E-01
83	1-ethyl-Cyclohexene	Hydrocarbons	1453-24-3	7.82E-03	8.28E-03	7.43E-03	1.30E-03	1.07E-03	1.25E-03	1.26E-03	1.25E-03	1.09E-03	1.09E-03	1.27E-03	1.32E-03
84	1-iodo-Decane	Others	2050-77-3	9.30E-03	1.17E-02	6.40E-03	6.09E-02	4.68E-02	4.16E-02	1.61E-01	1.47E-01	1.07E-01	5.23E-02	5.88E-02	6.55E-02
85	1-methyl-1H-Pyrrole-2-carboxaldehyde	Heterocyclic compounds	1192-58-1	7.33E-03	7.82E-03	8.19E-03	8.07E-03	6.71E-03	6.49E-03	8.10E-03	9.75E-03	8.29E-03	7.68E-03	8.13E-03	8.32E-03
86	1-methyl-3-(1-methylethyl)-Cyclohexane	Hydrocarbons	16580-24-8	7.56E+00	8.77E+00	8.40E+00	5.30E+00	4.63E+00	4.27E+00	4.51E+00	5.61E+00	4.95E+00	4.65E+00	4.64E+00	4.57E+00
87	1-methyl-3-(2-methyl-2-propenyl)-Cyclopentane	Hydrocarbons	75873-00-6	2.23E-02	2.49E-02	2.41E-02	1.30E-02	1.02E-02	9.86E-03	1.47E-02	1.68E-02	1.42E-02	1.08E-02	1.16E-02	1.18E-02
88	1-methyl-4-(1-methylethyl)-Cyclohexanol	Alcohols	21129-27-1	2.68E-02	2.92E-02	2.73E-02	1.92E-02	1.27E-02	1.15E-02	1.46E-02	1.89E-02	1.95E-02	2.02E-02	2.27E-02	2.71E-02
89	1-methyl-Piperazine	Heterocyclic compounds	109-01-3	6.17E+00	6.78E+00	6.15E+00	1.46E+00	1.22E+00	1.20E+00	1.29E+00	1.55E+00	1.30E+00	1.92E+00	1.97E+00	2.02E+00
90	1-octenyl-Benzene	Aromatics	29518-72-7	2.66E-04	2.66E-04	2.81E-04	3.90E-03	3.57E-03	1.92E-03	1.43E-02	1.50E-02	1.10E-02	1.03E-02	1.22E-02	1.04E-02

91	13-Oxabicyclo[10.1.0]tridecane	Hydrocarbons	286-99-7	4.69E-02	4.60E-02	3.76E-02	3.71E-02	1.63E-02	1.64E-02	7.76E-02	7.25E-02	5.06E-02	2.57E-02	2.95E-02	3.18E-02
92	1H-Tetrazol-5-amine	Heterocyclic compounds	4418-61-5	1.15E-01	1.36E-01	7.52E-02	1.91E-01	9.22E-02	8.35E-02	1.71E-01	1.49E-01	1.35E-01	1.31E-01	1.70E-01	1.61E-01
93	2'-methyl-Propiophenone	Ketones	2040-14-4	8.63E+01	7.98E+01	8.05E+01	6.80E+01	5.71E+01	4.90E+01	5.51E+01	6.35E+01	5.98E+01	5.70E+01	6.46E+01	7.43E+01
94	2,2'-Ethylidenebis(5-methylfuran)	Heterocyclic compounds	3209-79-8	9.19E-03	8.37E-03	7.18E-03	5.64E-03	2.97E-03	3.15E-03	4.07E-03	4.99E-03	4.16E-03	3.89E-03	4.31E-03	4.43E-03
95	2,2-Dimethyl-6-vinyl-2H-chromene	Heterocyclic compounds	164984-44-5	1.04E-04	1.04E-04	1.09E-04	1.19E-03	6.39E-04	7.65E-04	1.66E-03	2.00E-03	1.74E-03	1.55E-03	1.94E-03	2.26E-03
96	2,2-dihydroxy-1-phenyl-Ethanone	Ketones	1075-06-5	1.54E+00	1.49E+00	1.19E+00	6.30E-01	3.77E-01	3.61E-01	5.46E-01	6.22E-01	5.22E-01	5.31E-01	6.20E-01	6.68E-01
97	2,2-dimethyl-Cyclopentanone	Ketones	4541-32-6	8.60E-03	8.53E-03	1.02E-02	1.74E-03	1.44E-03	1.68E-03	8.44E-03	1.68E-03	1.46E-03	1.47E-03	1.70E-03	1.11E-02
98	2,2-dimethyl-Undecane	Hydrocarbons	17312-64-0	7.90E-02	8.70E-02	8.11E-02	7.38E-02	6.48E-02	1.25E-02	6.71E-02	8.29E-02	8.18E-02	5.70E-02	1.27E-02	6.63E-02
99	2,3,4-Trifluorobenzoic acid, cyclobutyl ester	Esters	1000283-00-1	6.46E+00	6.18E+00	5.36E+00	1.97E+00	1.16E+00	1.22E+00	1.81E+00	1.96E+00	1.68E+00	1.38E+00	1.62E+00	1.73E+00
100	2,3,6,7-tetramethyl-Octane	Hydrocarbons	52670-34-5	2.35E-03	2.35E-03	2.48E-03	1.19E-01	1.01E-01	9.66E-02	3.40E-03	3.39E-03	2.94E-03	1.90E-02	1.99E-02	1.99E-02
101	2,3-dimethyl-Heptane	Hydrocarbons	3074-71-3	3.10E-02	3.81E-02	3.35E-02	2.07E-02	1.60E-02	1.42E-02	2.45E-02	2.10E-02	1.97E-02	1.33E-02	1.58E-02	2.05E-02
102	2,4,6-Pyrimidinetriamine	Heterocyclic compounds	1004-38-2	3.70E-04	7.36E-04	6.54E-04	6.21E-04	3.71E-04	4.51E-04	7.67E-04	9.38E-04	7.33E-05	4.04E-04	8.65E-04	4.46E-04
103	2,4,6-trimethyl-Decane	Hydrocarbons	62108-27-4	5.60E-04	3.55E-03	2.95E-03	7.22E-03	6.14E-03	5.57E-03	4.77E-03	5.67E-03	5.84E-03	4.50E-03	4.33E-03	6.48E-03
104	2,4-Diamino-6-methyl-1,3,5-triazine	Heterocyclic compounds	542-02-9	4.12E-02	3.61E-02	3.07E-02	2.66E-02	1.50E-02	1.42E-02	3.11E-02	3.20E-02	2.95E-02	1.89E-02	2.33E-02	2.41E-02
105	2,4-dimethyl-1-(1-methylethyl)-Benzene	Aromatics	4706-89-2	9.51E-03	2.22E-02	1.59E-02	1.28E-02	1.12E-02	1.03E-02	2.06E-02	1.58E-02	1.61E-02	1.32E-02	1.91E-02	1.29E-02
106	2,4-dimethyl-2,4-Heptadiene	Hydrocarbons	74421-05-9	1.19E-02	1.21E-02	9.62E-03	7.34E-04	6.05E-04	7.06E-04	7.10E-04	7.08E-04	6.14E-04	6.17E-04	7.15E-04	7.47E-04
107	2,5-Diethylphenol	Phenols	876-20-0	3.32E-02	3.61E-02	3.14E-02	3.44E-02	1.92E-02	2.27E-02	2.84E-02	2.77E-02	2.54E-02	1.85E-02	2.07E-02	2.25E-02

108	2,5-Dimethylfuran-3-thiol	Heterocyclic compounds	55764-23-3	5.18E-01	5.52E-01	5.65E-01	5.24E-01	4.25E-01	4.13E-01	5.94E-01	6.93E-01	5.77E-01	2.64E-01	2.81E-01	2.80E-01
109	2,6,6-trimethyl-1-Cyclohexene-1-acetAldehyde	Aldehydes	472-66-2	3.85E-04	3.85E-04	4.06E-04	6.83E-03	2.37E-03	3.36E-03	5.38E-03	3.94E-03	2.80E-03	4.91E-03	5.58E-03	6.43E-03
110	2,6,7-trimethyl-Decane	Hydrocarbons	62108-25-2	5.60E-04	3.55E-03	2.95E-03	7.22E-03	6.14E-03	5.57E-03	4.77E-03	5.67E-03	5.84E-03	4.50E-03	4.33E-03	6.48E-03
111	2,6,8-trimethyl-Decane	Hydrocarbons	62108-26-3	5.60E-04	3.55E-03	2.95E-03	7.22E-03	6.14E-03	5.57E-03	4.77E-03	5.67E-03	5.84E-03	4.50E-03	4.33E-03	6.48E-03
112	2,6-Dimethyl-1,3,5,7-octatetraene, E,E-	Terpenoids	460-01-5	5.51E+00	6.07E+00	5.86E+00	3.13E+00	2.56E+00	2.47E+00	3.48E+00	4.03E+00	3.37E+00	2.60E+00	2.78E+00	2.82E+00
113	2,6-Dimethyl-2-trans-6-octadiene	Terpenoids	2609-23-6	1.83E+01	2.04E+01	1.83E+01	6.18E-01	4.36E-01	4.37E-01	4.54E+00	5.49E+00	4.62E+00	5.70E+00	5.84E+00	5.98E+00
114	2,6-Nonadienal, (E,Z)-	Aldehydes	557-48-2	3.56E+00	4.04E+00	4.01E+00	2.51E+00	2.04E+00	1.65E+00	2.01E+00	2.50E+00	2.78E+00	2.35E+00	1.54E+00	2.28E+00
115	2,6-Octadien-1-ol, 3,7-dimethyl-, (Z)-	Alcohols	106-25-2	2.06E+01	1.61E+02	1.87E+02	1.82E+02	1.48E+02	1.48E+02	1.63E+02	1.77E+02	1.61E+02	1.60E+02	1.82E+02	2.05E+02
116	2,6-Octadienal, 3,7-dimethyl-, (E)-	Terpenoids	141-27-5	9.40E+00	9.77E+00	9.48E+00	8.12E+00	6.13E+00	5.39E+00	5.99E+00	8.51E+00	7.29E+00	7.33E+00	7.25E+00	7.93E+00
117	2,6-Octadiene, 2,6-dimethyl-	Hydrocarbons	2792-39-4	1.83E+01	2.04E+01	1.83E+01	6.18E-01	4.36E-01	4.37E-01	4.54E+00	5.49E+00	4.62E+00	5.70E+00	5.84E+00	5.98E+00
118	2,7,10-trimethyl-Dodecane	Hydrocarbons	74645-98-0	1.36E-03	1.42E-03	1.05E-03	1.35E-03	9.73E-04	1.12E-03	1.30E-03	1.48E-03	1.19E-03	1.46E-03	1.67E-03	1.52E-03
119	2,7,7-trimethyl-Bicyclo[2.2.1]hept-2-ene	Hydrocarbons	514-14-7	8.39E-01	9.40E-01	8.94E-01	7.26E-01	6.24E-01	6.10E-01	8.42E-01	9.65E-01	8.09E-01	6.43E-01	6.74E-01	6.96E-01
120	2,7-Octadien-1-ol	Alcohols	23578-51-0	1.59E-01	1.87E-01	1.69E-01	1.02E-01	8.06E-02	7.75E-02	1.18E-01	1.44E-01	1.45E-01	9.34E-02	1.13E-01	1.13E-01
121	2,7-dimethyl-2,6-Octadiene	Hydrocarbons	16736-42-8	1.83E+01	2.04E+01	1.83E+01	6.18E-01	4.36E-01	4.37E-01	4.54E+00	5.49E+00	4.62E+00	5.70E+00	5.84E+00	5.98E+00
122	2,7-dimethyl-Oxepine	Heterocyclic compounds	1487-99-6	1.16E-01	1.23E-01	1.25E-01	1.01E-01	8.88E-02	8.61E-02	1.11E-01	1.33E-01	1.11E-01	8.61E-02	8.96E-02	9.59E-02
123	2,8-dimethyl-Undecane	Hydrocarbons	17301-25-6	1.42E-01	1.92E-01	1.73E-01	1.51E-01	1.21E-01	1.13E-01	1.84E-01	2.41E-01	2.27E-01	1.39E-01	1.27E-01	1.60E-01
124	2-(2-butoxyethoxy)-Ethanol, acetate	Esters	124-17-4	1.02E-02	1.90E-02	1.49E-02	4.68E-02	2.29E-02	2.65E-02	7.91E-02	6.91E-02	5.89E-02	2.69E-02	3.77E-02	2.96E-02

125	2-(4-Bromobutyl)-furan	Heterocyclic compounds	66356-49-8	1.08E+02	9.96E+01	1.02E+02	8.34E+01	6.77E+01	6.28E+01	7.09E+01	7.97E+01	7.58E+01	7.28E+01	8.23E+01	9.44E+01
126	2-(4-bromobutyl)-5,5-dimethyl-1,3-Cyclopentadiene	Halogenated hydrocarbons	1010163-68-7	2.77E-01	2.61E-01	2.20E-01	1.73E-01	8.58E-02	7.87E-02	1.35E-01	1.42E-01	1.41E-01	1.26E-01	1.45E-01	1.63E-01
127	2-(hydroxymethyl)-2-methyl-1,3-Propanediol	Alcohols	77-85-0	1.91E-03	1.91E-03	2.02E-03	1.82E-02	1.65E-02	1.38E-02	2.01E-02	2.54E-02	3.01E-02	1.74E-02	1.83E-02	2.91E-03
128	2-Acetylcyclopentanone	Ketones	1670-46-8	4.90E-02	5.24E-02	5.22E-02	4.20E-02	5.20E-02	4.83E-02	3.20E-02	3.47E-02	3.48E-02	5.73E-02	6.10E-02	7.13E-02
129	2-Butanone, 4-(2,6,6-trimethyl-1-cyclohexen-1-yl)-	Ketones	17283-81-7	1.51E-01	1.36E-01	1.07E-01	6.77E-02	3.95E-02	4.19E-02	6.86E-02	7.46E-02	6.18E-02	5.52E-02	6.59E-02	6.74E-02
130	2-Buten-1-one, 1-(2,6,6-trimethyl-1,3-cyclohexadien-1-yl)-, (E)-	Terpenoids	23726-93-4	9.30E-02	7.84E-02	6.28E-02	5.38E-02	3.04E-02	2.80E-02	6.16E-02	6.75E-02	5.48E-02	5.22E-02	6.04E-02	6.05E-02
131	2-Butoxyethyl acetate	Esters	112-07-2	2.46E-01	2.94E-01	2.89E-01	1.54E-01	1.22E-01	1.20E-01	1.54E-01	1.76E-01	1.75E-01	1.16E-01	1.25E-01	1.31E-01
132	2-Cyclopenten-1-one, 3-methyl-2-(2-pentenyl)-, (Z)-	Ketones	488-10-8	5.60E-03	9.32E-03	8.42E-03	6.59E-03	3.54E-03	4.50E-03	7.94E-03	5.46E-03	4.47E-03	4.26E-03	9.26E-03	4.79E-03
133	2-Decanol	Alcohols	1120-06-5	3.77E-02	4.25E-02	4.17E-02	6.61E-02	5.60E-02	5.18E-02	5.74E-02	6.55E-02	6.34E-02	5.21E-02	5.25E-02	4.89E-02
134	2-Decenal, (Z)-	Aldehydes	2497-25-8	8.67E+01	7.83E+01	8.14E+01	6.61E+01	5.32E+01	4.91E+01	5.64E+01	6.40E+01	5.92E+01	5.56E+01	6.29E+01	7.24E+01
135	2-Ethylpiperazine	Heterocyclic compounds	13961-37-0	2.67E-01	3.08E-01	2.91E-01	2.43E-01	2.72E-01	2.05E-01	1.52E-01	1.63E-01	1.73E-01	2.97E-01	2.72E-01	3.14E-01
136	2-Ethylpiperidine	Heterocyclic compounds	1484-80-6	2.15E+01	2.57E+01	2.43E+01	1.37E+01	1.21E+01	1.07E+01	1.15E+01	1.49E+01	1.30E+01	1.22E+01	1.21E+01	1.18E+01
137	2-FurancarboxAldehyde, 5-methyl-	Aldehydes	620-02-0	2.40E+00	2.75E+00	2.59E+00	2.71E+00	2.42E+00	2.19E+00	2.33E+00	2.05E+00	1.64E+00	2.06E+00	2.22E+00	2.34E+00
138	2-Hydroxymethyl-2-methyl-pyrrolidine-1-carboxaldehyde	Heterocyclic compounds	1000189-82-1	1.80E+02	1.86E+02	1.90E+02	2.37E+02	1.75E+02	1.76E+02	1.91E+02	2.07E+02	1.87E+02	1.93E+02	2.20E+02	2.49E+02
139	2-Isobutyl-4-methylpyridine	Heterocyclic compounds	85665-88-9	1.21E+00	1.52E+00	1.49E+00	7.98E-01	7.22E-01	5.43E-01	6.59E-01	8.31E-01	9.46E-01	7.83E-01	7.13E-01	8.41E-01

140	2-Methoxythiophene	Heterocyclic compounds	16839-97-7	3.30E-01	3.99E-01	3.67E-01	3.25E-01	2.82E-01	2.64E-01	3.44E-01	3.14E-01	2.79E-01	2.94E-01	3.08E-01	3.21E-01
141	2-Methyl-1-nonene-3-yne	Hydrocarbons	70058-00-3	1.11E+00	1.24E+00	1.13E+00	2.93E-01	2.48E-01	2.42E-01	3.15E-01	3.68E-01	3.18E-01	2.88E-01	2.99E-01	3.01E-01
142	2-Methyl-3-furanthiol	Heterocyclic compounds	28588-74-1	9.77E-04	1.08E-03	8.68E-04	2.46E-04	2.03E-04	2.37E-04	2.38E-04	2.38E-04	2.06E-04	2.07E-04	2.40E-04	2.51E-04
143	2-Methylheptanoic Acid	Acids	1188-02-9	1.08E+00	1.27E+00	1.08E+00	8.41E-01	8.16E-01	6.63E-01	5.81E-01	5.86E-01	5.77E-01	9.99E-01	9.07E-01	1.05E+00
144	2-Methylisoborneol	Terpenoids	2371-42-8	1.35E+01	1.01E+01	1.06E+01	7.68E+00	6.89E+00	5.32E+00	6.22E+00	7.71E+00	8.57E+00	7.46E+00	7.06E+00	6.76E+00
145	2-Methylthiolane, S,S-dioxide	Sulfur compounds	1003-46-9	1.83E+02	1.71E+02	1.95E+02	1.92E+02	1.57E+02	1.55E+02	1.70E+02	1.86E+02	1.67E+02	1.70E+02	1.92E+02	2.14E+02
146	2-Nonanol	Alcohols	628-99-9	8.43E-03	1.23E-02	8.91E-03	3.53E-02	2.99E-02	2.66E-02	5.61E-02	5.11E-02	4.71E-02	4.79E-02	4.60E-02	3.28E-02
147	2-Nonanone	Ketones	821-55-6	4.63E-02	5.35E-02	4.60E-02	4.24E-02	3.86E-02	3.58E-02	2.95E-02	2.69E-02	2.95E-02	4.44E-02	4.17E-02	4.62E-02
148	2-Nonanone, 3-(hydroxymethyl)-	Ketones	67801-33-6	3.19E-01	3.68E-01	3.30E-01	3.13E-01	2.63E-01	2.36E-01	2.42E-01	2.38E-01	2.41E-01	3.47E-01	3.32E-01	3.84E-01
149	2-Nonen-1-ol	Alcohols	22104-79-6	3.17E-01	3.97E-01	3.94E-01	2.23E-01	1.86E-01	1.58E-01	1.90E-01	3.15E-01	3.37E-01	2.71E-01	2.50E-01	2.93E-01
150	2-Nonenal, (Z)-	Aldehydes	60784-31-8	8.60E-04	9.33E-04	9.31E-04	2.49E-03	2.03E-03	1.95E-03	1.68E-03	1.86E-03	1.81E-03	2.16E-04	2.51E-04	2.62E-04
151	2-Octanone	Ketones	111-13-7	6.17E+00	6.68E+00	6.15E+00	1.46E+00	1.22E+00	1.12E+00	1.29E+00	1.44E+00	1.30E+00	1.80E+00	1.84E+00	1.88E+00
152	2-Octen-1-ol	Alcohols	22104-78-5	5.50E+00	6.62E+00	6.16E+00	5.28E+00	4.52E+00	4.28E+00	4.30E+00	3.99E+00	4.08E+00	4.93E+00	5.14E+00	5.63E+00
153	2-Octen-1-ol, (E)-	Alcohols	18409-17-1	5.50E+00	6.62E+00	6.16E+00	5.28E+00	4.52E+00	4.28E+00	4.30E+00	3.99E+00	4.08E+00	4.93E+00	5.14E+00	5.63E+00
154	2-Pentoxy-tetrahydropyran	Heterocyclic compounds	32767-70-7	9.09E-01	1.01E+01	1.06E+01	8.95E+00	7.33E+00	6.54E+00	7.43E+00	8.46E+00	8.03E+00	7.53E+00	8.62E+00	9.83E+00
155	2-Phenylpropenal	Aromatics	4432-63-7	1.58E-01	1.64E-01	1.75E-01	1.03E-01	8.74E-02	7.02E-02	8.24E-02	9.89E-02	1.10E-01	9.76E-02	8.31E-02	1.01E-01
156	2-Piperidinone	Heterocyclic compounds	675-20-7	2.49E-02	2.62E-02	2.81E-02	2.03E-02	2.02E-02	1.70E-02	1.65E-02	2.03E-02	1.98E-02	1.78E-02	1.64E-02	1.79E-02
157	2-Pyrrolidinone	Heterocyclic compounds	616-45-5	1.80E-02	1.90E-02	1.99E-02	3.46E-02	2.92E-02	2.73E-02	2.91E-02	2.61E-02	2.56E-02	2.36E-02	2.50E-02	2.74E-02

158	2-Tridecanol	Alcohols	1653-31-2	5.83E-04	5.84E-04	6.15E-04	1.15E-02	4.96E-03	4.20E-03	5.19E-03	5.72E-03	5.15E-03	1.41E-02	1.82E-02	2.06E-02
159	2-Tridecanone	Ketones	593-08-8	9.13E-03	1.77E-02	9.93E-03	1.77E-02	8.01E-03	8.96E-03	1.91E-02	1.95E-02	1.34E-02	1.82E-02	2.34E-02	1.90E-02
160	2-amino-1-phenyl-Ethanone	Ketones	613-89-8	8.17E+00	8.58E+00	8.39E+00	1.13E+01	7.58E+00	7.24E+00	7.82E+00	8.91E+00	7.96E+00	8.19E+00	9.18E+00	1.05E+01
161	2-butenyl-Benzene	Aromatics	1560-06-1	1.04E+01	1.10E+01	9.51E+00	7.00E+00	5.71E+00	6.37E+00	7.58E+00	7.06E+00	7.35E+00	5.24E+00	5.10E+00	5.58E+00
162	2-chloro-Octane	Halogenated hydrocarbons	628-61-5	1.36E-02	1.52E-02	1.57E-02	1.31E-02	1.18E-02	1.06E-02	1.47E-02	1.80E-02	1.55E-02	9.75E-03	9.42E-03	1.00E-02
163	2-cyano-Imidazole	Heterocyclic compounds	31722-49-3	6.13E+00	7.22E+00	7.28E+00	3.86E+00	3.25E+00	2.81E+00	3.34E+00	4.09E+00	4.38E+00	3.81E+00	3.54E+00	4.25E+00
164	2-deoxy-D-erythro-Pentose	Aldehydes	533-67-5	7.86E-01	8.13E-01	8.00E-01	9.56E-01	6.52E-01	6.40E-01	7.04E-01	7.86E-01	7.16E-01	7.19E-01	8.13E-01	9.41E-01
165	2-ethyl-2-Hexenal	Aldehydes	645-62-5	4.58E+00	5.24E+00	4.59E+00	9.86E-01	8.33E-01	8.27E-01	8.70E-01	1.04E+00	8.59E-01	1.29E+00	1.33E+00	1.41E+00
166	2-ethyl-4-methyl-1-Pentanol	Alcohols	106-67-2	2.35E-02	2.77E-02	2.71E-02	1.67E-02	1.40E-02	1.20E-02	1.99E-02	1.96E-02	1.65E-02	1.52E-02	1.70E-02	2.53E-03
167	2-methoxy-2-Octen-4-one	Ketones	24985-48-6	1.12E-03	1.12E-03	1.18E-03	1.10E-02	9.89E-03	1.09E-02	8.11E-03	9.74E-03	7.50E-03	1.41E-03	9.47E-03	1.71E-03
168	2-methoxy-6-methyl-4H-Pyran-4-one	Heterocyclic compounds	4225-42-7	6.03E-02	7.00E-02	6.18E-02	5.70E-02	5.26E-02	4.80E-02	4.24E-02	4.64E-02	4.41E-02	6.84E-02	6.58E-02	7.20E-02
169	2-methoxy-Phenol	Phenols	90-05-1	1.19E+00	1.38E+00	1.18E+00	1.16E+00	1.00E+00	8.85E-01	8.27E-01	8.99E-01	8.85E-01	1.45E+00	1.35E+00	1.55E+00
170	2-methyl-1H-Indole	Heterocyclic compounds	95-20-5	7.02E-03	6.05E-03	5.33E-03	4.28E-03	1.86E-03	2.03E-03	3.98E-03	3.89E-03	3.94E-03	3.36E-03	4.80E-03	4.99E-03
171	2-methyl-2-Decene	Hydrocarbons	23381-92-2	5.25E+00	6.09E+00	5.36E+00	5.29E+00	4.48E+00	3.93E+00	4.07E+00	4.54E+00	4.21E+00	6.43E+00	5.96E+00	6.84E+00
172	2-methyl-2-Undecene	Hydrocarbons	56888-88-1	4.37E-01	4.98E-01	3.72E-01	5.10E-01	2.73E-01	2.37E-01	3.73E-01	3.79E-01	4.39E-01	4.05E-01	4.39E-01	3.90E-01
173	2-methyl-3-Pentanol	Alcohols	565-67-3	5.97E-01	7.33E-01	6.86E-01	6.55E-01	5.66E-01	4.97E-01	6.34E-01	5.22E-01	3.51E-01	6.04E-01	5.69E-01	5.59E-01
174	2-methyl-5-(methylthio)-Furan	Heterocyclic compounds	13678-59-6	2.90E-02	3.29E-02	2.84E-02	8.11E-04	6.69E-04	3.90E-03	7.85E-04	7.82E-04	6.79E-04	4.00E-03	4.87E-03	4.75E-03
175	2-methyl-Butanoic acid,2-methyl-2-propenyl ester	Esters	83783-90-8	1.57E-01	1.77E-01	1.56E-01	8.16E-02	7.05E-02	6.56E-02	3.90E-02	4.39E-02	4.37E-02	9.45E-02	1.01E-01	9.79E-02
176	2-methyl-Decanoic acid	Acids	24323-23-7	2.48E-02	2.21E-02	1.80E-02	4.03E-03	3.33E-03	3.88E-03	3.12E-02	3.11E-02	2.71E-02	3.39E-03	3.93E-03	4.10E-03

177	2-methyl-Octanoic acid	Acids	3004-93-1	5.79E-04	5.80E-04	6.11E-04	6.22E-03	8.11E-03	4.17E-03	1.19E-02	1.18E-02	1.19E-02	8.02E-03	1.15E-02	8.32E-03
178	2-n-Octylfuran	Heterocyclic compounds	4179-38-8	3.03E-02	2.94E-02	2.45E-02	2.64E-02	1.59E-02	1.57E-02	3.29E-02	1.65E-02	1.82E-02	1.29E-02	1.79E-02	1.83E-02
179	2-phenoxy-1-Propanol	Alcohols	4169-04-4	1.60E+02	1.51E+02	1.51E+02	1.46E+02	1.20E+02	1.12E+02	1.24E+02	1.37E+02	1.31E+02	1.26E+02	1.42E+02	1.62E+02
180	2-propenyl-Benzene	Aromatics	300-57-2	6.45E-02	6.86E-02	6.75E-02	1.97E-02	1.29E-02	1.20E-02	3.86E-02	4.73E-02	4.12E-02	2.34E-02	2.44E-02	2.41E-02
181	2-propenylidene-Cyclobutene	Hydrocarbons	52097-85-5	4.40E-04	4.40E-04	4.64E-04	4.33E-03	3.29E-03	3.29E-03	4.74E-03	4.08E-03	5.15E-03	3.68E-03	6.41E-04	6.70E-04
182	2H-1,2-Oxaborin, 2,3,3-triethyl-3,6-dihydro-	Heterocyclic compounds	32765-44-9	5.77E+01	4.90E+01	4.80E+01	4.15E+01	3.69E+01	3.08E+01	3.37E+01	4.21E+01	4.33E+01	3.56E+01	3.74E+01	3.94E+01
183	2H-Indol-2-one, 1,3-dihydro-	Ketones	59-48-3	1.81E-02	1.94E-02	1.16E-02	2.38E-02	2.03E-02	1.70E-02	4.23E-02	4.76E-02	3.65E-02	5.10E-02	6.04E-02	4.84E-02
184	2H-Pyran-2-one, tetrahydro-6-methyl-	Ketones	823-22-3	3.41E-01	3.70E-01	3.42E-01	2.46E-01	1.95E-01	1.64E-01	2.12E-01	2.09E-01	2.01E-01	3.01E-01	2.95E-01	3.16E-01
185	2H-Pyran-2-one, tetrahydro-6-pentyl-	Esters	705-86-2	2.86E-02	5.55E-02	2.51E-02	5.28E-02	3.07E-02	2.81E-02	4.72E-02	5.09E-02	3.15E-02	5.36E-02	6.43E-02	4.14E-02
186	3,3-dimethyl-2-Hexanone	Ketones	26118-38-7	2.52E-01	3.13E-01	2.58E-01	3.29E-01	3.17E-01	2.36E-01	3.29E-01	3.13E-01	3.06E-01	2.61E-01	2.91E-01	3.02E-01
187	3,4,5-trimethyl-2-Cyclopenten-1-one	Ketones	55683-21-1	1.61E-01	1.74E-01	1.77E-01	1.34E-01	1.24E-01	1.15E-01	1.48E-01	9.20E-02	2.82E-02	1.51E-01	1.16E-01	1.73E-01
188	3,4-bis(1,1-dimethylethyl)-2,2,5,5-tetramethyl-Hexane	Hydrocarbons	62850-21-9	3.05E-02	2.70E-02	2.09E-02	1.17E-03	9.63E-04	1.12E-03	1.13E-03	1.13E-03	9.77E-04	4.97E-03	5.95E-03	5.94E-03
189	3,4-dihydro-6-methyl-2H-Pyran	Heterocyclic compounds	16015-11-5	1.90E+00	1.75E+00	1.75E+00	1.44E+00	1.22E+00	1.05E+00	1.17E+00	1.34E+00	1.27E+00	1.25E+00	1.41E+00	1.61E+00
190	3,4-dihydroxy-3,4-dimethyl-2,5-Hexanedione	Ketones	28123-56-0	1.05E-01	8.91E-02	9.22E-02	8.50E-02	6.09E-02	6.20E-02	6.53E-02	6.87E-02	6.93E-02	6.88E-02	7.72E-02	8.84E-02
191	3,5,9-Undecatrien-2-one, 6,10-dimethyl-	Ketones	141-10-6	8.09E-01	5.65E-01	4.34E-01	2.76E-01	1.66E-01	1.54E-01	2.79E-01	3.13E-01	2.54E-01	2.54E-01	2.94E-01	3.12E-01
192	3,5-Heptanedione, 4-ethyl-2,2,6,6-tetramethyl-	Ketones	167545-33-7	2.26E-02	1.87E-02	1.53E-02	1.91E-02	1.09E-02	1.05E-02	1.39E-02	1.31E-02	1.24E-02	2.21E-02	2.43E-02	1.77E-02
193	3,5-Octadien-2-one	Ketones	38284-27-4	2.20E-01	2.44E-01	2.35E-01	1.25E-01	7.01E-02	9.82E-02	1.37E-01	1.63E-01	1.35E-01	1.01E-01	1.12E-01	1.11E-01

194	3,5-Octadien-2-one, (E,E)-	Ketones	30086-02-3	2.20E-01	2.44E-01	2.35E-01	1.25E-01	7.01E-02	9.82E-02	1.37E-01	1.63E-01	1.35E-01	1.01E-01	1.12E-01	1.11E-01
195	3,6-Dimethyl-2,3,3a,4,5,7a-hexahydrobenzofuran	Heterocyclic compounds	70786-44-6	1.92E-01	2.26E-01	1.98E-01	1.56E-01	1.43E-01	1.30E-01	1.18E-01	1.26E-01	1.28E-01	2.24E-01	2.15E-01	2.64E-01
196	3,6-Nonadien-1-ol, (E,Z)-	Alcohols	56805-23-3	3.24E+01	3.52E+01	3.43E+01	2.98E+01	2.60E+01	2.31E+01	2.46E+01	3.03E+01	3.10E+01	2.73E+01	2.67E+01	2.79E+01
197	3,7,7-trimethyl-Bicyclo[4.1.0]heptane	Hydrocarbons	554-59-6	9.01E-02	1.09E-01	1.00E-01	5.96E-02	4.84E-02	4.64E-02	6.81E-02	7.52E-02	6.43E-02	4.64E-02	5.04E-02	4.99E-02
198	3,8-dimethyl-Decane	Hydrocarbons	17312-55-9	2.30E-03	2.03E-03	2.50E-03	3.76E-03	2.72E-03	2.44E-03	1.35E-03	1.68E-03	2.15E-03	1.04E-03	1.36E-03	1.35E-03
199	3-(2-propenyl)-Cyclohexene	Hydrocarbons	15232-95-8	6.48E-02	7.28E-02	6.82E-02	5.52E-02	5.37E-02	4.63E-02	6.29E-02	7.07E-02	6.00E-02	5.52E-02	5.12E-02	5.19E-02
200	3-(4-tert-Butylphenyl)isobutyraldehyde	Aldehydes	80-54-6	6.93E-02	6.26E-02	4.76E-02	4.20E-02	2.65E-02	2.39E-02	6.24E-02	6.21E-02	5.37E-02	5.34E-02	6.44E-02	6.48E-02
201	3-(methylthio)-1,2-Propanediol	Alcohols	22551-26-4	4.81E-03	4.61E-03	3.67E-03	3.04E-03	4.67E-04	2.75E-03	2.74E-03	3.21E-03	4.29E-03	4.76E-04	2.93E-03	3.11E-03
202	3-Amino-5-pyrazolol	Heterocyclic compounds	6126-22-3	4.45E-02	3.96E-02	3.48E-02	4.36E-02	3.87E-02	3.51E-02	4.87E-02	5.36E-02	5.38E-02	5.38E-02	5.41E-02	5.86E-02
203	3-Carene	Terpenoids	13466-78-9	8.39E-01	9.40E-01	8.94E-01	7.26E-01	6.24E-01	6.10E-01	8.42E-01	9.65E-01	8.09E-01	6.43E-01	6.74E-01	6.96E-01
204	3-Cyclohexene-1-ethanol	Alcohols	18240-10-3	9.81E-01	1.13E+00	9.74E-01	3.16E-01	2.80E-01	2.55E-01	2.67E-01	2.81E-01	2.78E-01	4.04E-01	3.80E-01	4.34E-01
205	3-Cyclopentyl-1-propanol	Alcohols	767-05-5	1.10E-01	1.51E-01	1.18E-01	1.24E-01	1.19E-01	9.96E-02	9.83E-02	9.86E-02	1.06E-01	1.47E-01	1.34E-01	1.69E-01
206	3-Ethyl-2,6,10-trimethylundecane	Hydrocarbons	1000432-25-9	1.35E-02	1.23E-02	1.04E-02	1.25E-02	1.05E-03	6.13E-03	1.10E-02	9.39E-03	7.92E-03	1.04E-02	1.21E-02	1.32E-02
207	3-Ethylcyclopentanone	Ketones	10264-55-8	4.94E-02	5.23E-02	5.11E-02	5.73E-03	4.73E-03	5.52E-03	4.21E-02	4.44E-02	3.63E-02	4.82E-03	3.05E-02	2.92E-02
208	3-FurAldehyde	Aldehydes	498-60-2	7.70E-03	7.71E-03	8.12E-03	1.19E-01	1.17E-01	9.74E-02	1.29E-01	1.15E-01	1.03E-01	9.05E-02	8.58E-02	5.86E-02
209	3-Hexen-1-ol, propanoate, (Z)-	Esters	33467-74-2	4.73E-02	5.36E-02	5.41E-02	8.28E-02	6.01E-02	7.24E-02	6.87E-02	5.99E-02	5.12E-02	1.00E-01	9.95E-02	8.52E-02
210	3-Hydroxy-4-methylbenzAldehyde	Aldehydes	57295-30-4	9.01E-02	9.06E-02	7.59E-02	9.15E-02	5.33E-02	6.02E-02	8.19E-02	7.89E-02	6.99E-02	5.65E-02	6.65E-02	6.79E-02

211	3-Hydroxydecanoic acid	Acids	33044-91-6	1.74E-03	1.17E-03	1.35E-03	1.79E-03	1.44E-03	1.28E-03	4.75E-03	4.19E-03	3.66E-03	3.82E-03	4.54E-03	4.68E-03
212	3-Methyl-1-adamantanecarboxylic acid	Acids	1000504-38-9	5.58E-01	4.87E-01	4.18E-01	2.95E-01	1.63E-01	1.37E-01	2.74E-01	2.91E-01	2.78E-01	2.40E-01	2.79E-01	3.12E-01
213	3-Methyl-2-(2-methyl-2-butenyl)-furan	Heterocyclic compounds	15186-51-3	7.31E-01	7.96E-01	7.02E-01	5.45E-01	4.31E-01	3.87E-01	3.57E-01	4.34E-01	4.31E-01	4.19E-01	4.10E-01	4.66E-01
214	2-hydroxy-Propanoic acid	Acids	50-21-5	1.04E+00	1.25E+00	1.18E+00	1.65E+01	1.57E+01	1.51E+01	1.75E+01	1.91E+01	1.82E+01	2.58E+01	2.57E+01	2.56E+01
215	3-Nonen-1-ol, (Z)-	Alcohols	10340-23-5	3.17E-01	3.97E-01	3.94E-01	2.23E-01	1.86E-01	1.58E-01	1.90E-01	3.15E-01	3.37E-01	2.71E-01	2.50E-01	2.93E-01
216	3-Octanol	Alcohols	589-98-0	4.87E-01	5.91E-01	4.92E-01	1.25E-01	1.04E-01	1.01E-01	1.16E-01	1.32E-01	1.11E-01	1.64E-01	1.66E-01	1.69E-01
217	3-Phenylpropanol	Alcohols	122-97-4	8.53E-01	1.03E+00	9.12E-01	8.03E-01	6.87E-01	7.16E-01	8.10E-01	8.72E-01	7.62E-01	1.25E-01	1.45E-01	1.51E-01
218	3-Pyridinecarbonitrile	Heterocyclic compounds	100-54-9	3.15E-03	3.42E-03	3.23E-03	3.22E-03	2.49E-03	2.58E-03	3.51E-03	3.80E-03	3.39E-03	2.36E-03	2.57E-03	3.01E-03
219	3-butyl-2,5-dimethyl-Pyrazine	Heterocyclic compounds	40790-29-2	1.51E-03	1.04E+00	6.67E-01	3.02E-02	1.72E-02	1.79E-02	1.09E-02	4.47E-02	3.85E-02	3.51E-02	3.50E-02	2.97E-02
220	3-ethenyl-Cyclooctene	Hydrocarbons	2213-60-7	7.31E-02	8.20E-02	8.28E-02	7.15E-02	5.58E-02	5.49E-02	5.33E-02	6.90E-02	6.99E-02	6.64E-02	6.50E-02	7.30E-02
221	3-ethyl-Octane	Hydrocarbons	5881-17-4	3.56E-04	5.58E-04	5.50E-04	1.05E-04	1.93E-03	1.01E-04	5.09E-04	5.47E-04	4.92E-04	4.60E-04	8.23E-04	6.40E-04
222	3-ethyl-Pyridine	Heterocyclic compounds	536-78-7	5.01E-04	5.01E-04	5.28E-04	4.57E-03	4.36E-03	3.80E-03	3.95E-03	4.59E-03	3.13E-03	4.70E-03	4.60E-03	5.25E-03
223	3-hydroxy-Benzoic acid, methyl ester	Esters	19438-10-9	1.14E+01	1.19E+01	1.14E+01	1.59E+01	1.07E+01	1.01E+01	1.06E+01	1.24E+01	1.10E+01	1.12E+01	1.26E+01	1.44E+01
224	3-hydroxy-Butanoic acid	Acids	300-85-6	3.25E-03	3.25E-03	3.43E-03	9.58E-02	8.90E-02	7.91E-02	4.70E-03	4.69E-03	4.07E-03	2.16E-02	2.37E-02	2.47E-02
225	3-methyl-1H-Pyrazole	Heterocyclic compounds	1453-58-3	1.17E-03	1.17E-03	1.23E-03	1.75E-03	9.72E-03	8.42E-03	9.32E-03	9.07E-03	1.46E-03	1.47E-03	1.71E-03	1.78E-03
226	3-methyl-3-phenyl-Azetidine	Nitrogen compounds	5961-33-1	8.79E-03	1.33E-02	1.04E-02	1.58E-02	8.90E-03	1.09E-02	1.08E-02	9.18E-03	1.07E-02	7.32E-03	1.17E-02	8.08E-03
227	3-methyl-6-(1-methylethyl)-Cyclohexene	Hydrocarbons	5256-65-5	1.53E-01	1.96E-01	1.68E-01	1.59E-01	1.38E-01	1.32E-01	1.80E-01	2.12E-01	1.76E-01	1.39E-01	1.44E-01	1.50E-01
228	3-methyl-6-(1-methylethylidene)-Cyclohexene	Terpenoids	586-63-0	7.59E-01	8.05E-01	1.04E+00	6.21E-01	4.98E-01	5.05E-01	6.61E-01	7.61E-01	6.63E-01	4.64E-01	4.81E-01	5.01E-01
229	3-methyl-Butanamide	Amines	541-46-8	4.87E-01	5.36E-01	4.86E-01	1.14E-01	9.28E-02	9.15E-02	1.04E-01	1.20E-01	1.00E-01	1.48E-01	1.53E-01	1.54E-01

230	3-methyl-Tetradecane	Hydrocarbons	18435-22-8	4.84E-03	6.30E-03	3.50E-03	7.50E-03	6.08E-03	5.35E-03	6.42E-03	6.20E-03	4.99E-03	1.05E-02	1.21E-02	8.58E-03
231	3-phenyl-Furan	Heterocyclic compounds	13679-41-9	6.08E-04	1.08E-03	7.29E-05	6.62E-04	4.27E-04	9.96E-05	1.08E-03	1.48E-03	1.17E-03	9.55E-04	1.13E-03	1.27E-03
232	4'-Butoxyacetophenone	Ketones	5736-89-0	7.60E-03	1.35E-02	8.07E-03	1.56E-02	1.60E-02	1.30E-02	6.14E-02	6.35E-02	4.80E-02	4.21E-02	4.82E-02	5.18E-02
233	4(1H)-Pyridinone, 2,3-dihydro-1-methyl-	Heterocyclic compounds	35488-00-7	1.01E+01	1.15E+01	1.01E+01	2.26E+00	1.90E+00	1.93E+00	2.01E+00	2.37E+00	1.98E+00	2.98E+00	3.07E+00	3.26E+00
234	4-(1-methylethyl)-2-Cyclohexen-1-one	Ketones	500-02-7	9.40E-02	1.14E-01	1.01E-01	8.69E-02	6.88E-02	6.64E-02	1.34E-02	1.33E-02	1.15E-02	9.17E-02	9.97E-02	1.17E-01
235	4-(1-methylethyl)-Benzaldehyde	Terpenoids	122-03-2	2.24E+00	2.04E+00	2.02E+00	1.67E+00	1.41E+00	1.13E+00	1.31E+00	1.55E+00	1.47E+00	1.36E+00	1.53E+00	1.79E+00
236	4-(2-oxopropyl)-2-Cyclohexen-1-one	Ketones	56051-94-6	8.37E+01	7.63E+01	7.69E+01	7.17E+01	5.04E+01	4.97E+01	5.42E+01	6.07E+01	5.49E+01	5.62E+01	6.39E+01	7.29E+01
237	4-Allyl-1,6-heptadiene-4-ol	Alcohols	10202-75-2	4.60E-02	4.20E-02	3.73E-02	5.77E-02	4.74E-02	4.63E-02	7.64E-03	7.61E-03	6.61E-03	5.68E-02	5.67E-02	6.07E-02
238	4-Amino-4,5(1H)-dihydro-1,2,4-triazole-5-one	Ketones	1003-23-2	2.19E-02	2.93E-02	2.69E-02	1.35E-02	1.32E-02	1.08E-02	1.15E-02	1.51E-02	1.18E-02	1.31E-02	1.25E-02	1.18E-02
239	4-Cyanocyclohexene	Nitrogen compounds	100-45-8	4.51E+00	5.25E+00	4.72E+00	2.87E+00	2.43E+00	2.22E+00	2.49E+00	2.92E+00	2.70E+00	2.25E+00	2.36E+00	2.30E+00
240	4-Decene	Hydrocarbons	19689-18-0	2.11E+00	2.58E+00	2.41E+00	1.37E+00	1.20E+00	1.12E+00	1.18E+00	1.45E+00	1.29E+00	1.20E+00	1.22E+00	1.24E+00
241	4-Ethylcyclohexanol	Alcohols	4534-74-1	2.07E-02	2.20E-02	2.22E-02	2.81E-02	2.46E-02	2.28E-02	2.25E-02	1.63E-02	1.58E-02	1.95E-02	2.03E-02	1.93E-02
242	4-Hexen-1-ol, acetate	Others	72237-36-6	7.91E+00	9.52E+00	7.86E+00	2.01E+00	1.67E+00	1.67E+00	1.84E+00	2.19E+00	1.83E+00	2.52E+00	2.58E+00	2.66E+00
243	4-Nonanol	Alcohols	5932-79-6	8.43E-03	1.23E-02	8.91E-03	3.53E-02	2.99E-02	2.66E-02	5.61E-02	5.11E-02	4.71E-02	4.79E-02	4.60E-02	3.28E-02
244	4-Nonanone	Ketones	4485-09-0	4.63E-02	5.35E-02	4.60E-02	4.24E-02	3.86E-02	3.58E-02	2.95E-02	2.69E-02	2.95E-02	4.44E-02	4.17E-02	4.62E-02
245	4-Propoxy-2-butanone	Ketones	89975-71-3	1.54E-03	1.54E-03	1.62E-03	2.30E-03	1.90E-03	2.21E-03	2.22E-03	2.22E-03	1.92E-03	1.18E-02	1.84E-02	2.01E-02

246	4-Pyridinamine, N,N-dimethyl-	Heterocyclic compounds	1122-58-3	1.25E+00	1.37E+00	1.37E+00	8.06E-01	6.69E-01	6.54E-01	9.43E-01	1.07E+00	9.00E-01	6.43E-01	6.86E-01	6.91E-01
247	4-Quinolinecarboxaldehyde	Heterocyclic compounds	4363-93-3	9.74E-03	5.90E-03	4.24E-03	4.60E-03	4.65E-03	6.85E-03	5.38E-03	5.70E-03	4.67E-03	2.26E-03	2.99E-03	2.54E-03
248	4-Vinyl-imidazole	Heterocyclic compounds	3718-04-5	4.49E-02	4.95E-02	4.89E-02	2.85E-02	2.42E-02	2.28E-02	3.41E-02	3.77E-02	3.21E-02	2.22E-02	2.25E-02	2.19E-02
249	4-acetyl-3-Carene	Ketones	1000156-14-1	5.72E+00	5.20E+00	4.52E+00	5.22E+00	3.50E+00	3.24E+00	3.53E+00	4.02E+00	3.56E+00	3.60E+00	4.02E+00	4.66E+00
250	4-ethyl-Decane	Hydrocarbons	1636-44-8	1.69E-02	1.89E-02	1.76E-02	1.55E-02	1.49E-02	1.40E-02	2.29E-02	2.95E-02	2.88E-02	5.27E-02	5.42E-02	6.55E-02
251	4-methyl-Nonane	Hydrocarbons	17301-94-9	3.56E-04	5.58E-04	5.50E-04	1.05E-04	1.93E-03	1.01E-04	5.09E-04	5.47E-04	4.92E-04	4.60E-04	8.23E-04	6.40E-04
252	4-methyl-Octane	Hydrocarbons	2216-34-4	1.35E-03	1.80E-03	1.52E-03	1.70E-03	1.27E-03	6.73E-04	1.70E-03	1.13E-03	1.20E-03	1.23E-03	1.98E-03	2.24E-03
253	4-methyl-Pentanamide	Amines	1119-29-5	4.56E-04	4.56E-04	4.80E-04	2.12E-02	1.83E-02	1.74E-02	6.59E-04	6.57E-04	5.70E-04	3.06E-03	3.90E-03	4.43E-03
254	4-methylene-1-(1-methylethyl)-Cyclohexene	Hydrocarbons	99-84-3	1.70E+00	1.18E+01	9.63E+00	2.28E+00	1.95E+00	1.94E+00	2.29E+00	2.67E+00	2.32E+00	2.95E+00	3.05E+00	3.15E+00
255	4-sec-Butylaniline	Amines	30273-11-1	1.05E-02	1.07E-02	9.82E-03	9.56E-03	5.96E-03	1.24E-03	6.35E-03	6.71E-03	6.72E-03	5.40E-03	6.37E-03	6.78E-03
256	4-tert-Butylcyclohexyl acetate	Esters	32210-23-4	2.69E-02	3.79E-02	2.72E-02	2.92E-02	2.46E-02	2.35E-02	2.75E-02	2.45E-02	2.36E-02	3.10E-02	2.86E-02	3.11E-02
257	4H-1,2,4-Triazol-4-amine	Heterocyclic compounds	584-13-4	2.16E-01	2.42E-01	2.22E-01	8.64E-02	6.45E-02	6.28E-02	1.21E-01	9.93E-02	9.17E-02	7.15E-02	7.71E-02	8.29E-02
258	5,5-dimethyl-Undecane	Hydrocarbons	17312-73-1	7.90E-02	8.70E-02	8.11E-02	7.38E-02	6.48E-02	1.25E-02	6.71E-02	8.29E-02	8.18E-02	5.70E-02	1.27E-02	6.63E-02
259	5,9-Undecadien-2-ol, 6,10-dimethyl-	Alcohols	53837-34-6	3.83E-02	2.37E-02	2.63E-02	1.75E-02	7.81E-03	7.60E-03	2.00E-02	2.40E-02	1.55E-02	1.38E-02	1.46E-02	1.60E-02
260	5,9-Undecadien-2-one, 6,10-dimethyl-	Ketones	689-67-8	2.40E-02	1.98E-02	1.55E-02	1.44E-02	8.17E-03	7.06E-03	1.33E-02	1.45E-02	1.29E-02	1.21E-02	1.51E-02	1.52E-02
261	5,9-Undecadien-2-one, 6,10-dimethyl-, (E)-	Ketones	3796-70-1	6.58E-03	5.75E-03	5.06E-03	8.21E-03	4.59E-03	3.55E-03	5.21E-03	4.81E-03	4.68E-03	6.03E-03	7.90E-03	7.55E-03

262	5-(1,1-dimethylethyl)-2,4(1H, 3H)-Pyrimidinedione	Heterocyclic compounds	17432-97-2	1.42E-04	1.42E-04	1.50E-04	2.73E-03	1.27E-03	1.39E-03	1.99E-03	1.98E-03	1.09E-03	1.79E-04	1.04E-03	2.17E-04
263	5-Amino-2-methyl-2H-tetrazole	Heterocyclic compounds	6154-04-7	1.65E-02	1.63E-02	1.51E-02	1.23E-02	9.26E-03	9.10E-03	8.62E-03	1.17E-02	1.46E-02	8.53E-03	8.28E-03	8.54E-03
264	5-Decene	Hydrocarbons	19689-19-1	2.11E+00	2.58E+00	2.41E+00	1.37E+00	1.20E+00	1.12E+00	1.18E+00	1.45E+00	1.29E+00	1.20E+00	1.22E+00	1.24E+00
265	5-Hepten-2-one, 6-methyl-	Ketones	110-93-0	2.75E+01	3.08E+01	2.75E+01	5.93E+00	4.98E+00	5.03E+00	5.24E+00	6.21E+00	5.20E+00	7.87E+00	8.12E+00	8.50E+00
266	5-Hydroxymethylfurfural	Aldehydes	67-47-0	1.52E+00	1.66E+00	1.73E+00	4.57E-01	8.68E-01	6.52E-01	8.19E-01	1.02E+00	1.15E+00	9.89E-01	9.17E-01	9.85E-01
267	5-Methyl-4-hexene-1-yl acetate	Esters	1000426-93-8	7.20E-02	8.45E-02	7.93E-02	8.15E-02	7.23E-02	6.46E-02	5.08E-02	5.61E-02	5.76E-02	7.72E-02	7.39E-02	9.31E-02
268	5-hydroxy-2,4(1H,3H)-Pyrimidinedione	Heterocyclic compounds	20636-41-3	1.93E-03	4.39E-03	2.77E-03	5.32E-03	3.15E-03	3.27E-03	5.24E-03	4.35E-03	4.88E-03	4.18E-03	4.88E-03	3.55E-03
269	5-methyl-1H-Indole	Heterocyclic compounds	614-96-0	2.80E-01	3.07E-01	2.51E-01	4.07E-02	2.43E-02	2.47E-02	3.91E-02	4.09E-02	3.59E-02	3.05E-02	3.56E-02	3.89E-02
270	5-methyl-2-(1-methylethyl)-Cyclohexanol	Alcohols	1490-04-6	4.37E-01	5.16E-01	5.50E-01	3.12E-01	2.95E-01	2.25E-01	2.54E-01	3.13E-01	3.44E-01	2.88E-01	2.75E-01	3.22E-01
271	5-methyl-Hexanenitrile	Nitrogen compounds	19424-34-1	1.33E-02	1.46E-02	1.48E-02	3.00E-03	2.48E-03	2.89E-03	1.49E-02	1.60E-02	2.51E-03	1.26E-02	1.58E-02	1.65E-02
272	5-methyl-Nonane	Hydrocarbons	15869-85-9	2.11E-02	2.32E-02	2.21E-02	5.90E-03	4.86E-03	5.67E-03	3.23E-02	3.12E-02	2.47E-02	2.74E-02	2.93E-02	3.24E-02
273	5H-Tetrazol-5-Amine	Amines	1000273-02-0	1.93E-01	2.24E-01	2.23E-01	1.13E-01	9.23E-02	7.12E-02	1.02E-01	1.28E-01	1.43E-01	1.05E-01	9.38E-02	1.10E-01
274	6,8-Nonadien-2-one, 6-methyl-5-(1-methylethylidene)-	Heterocyclic compounds	60714-16-1	1.25E+00	1.11E+00	9.45E-01	6.97E-01	3.82E-01	3.50E-01	6.44E-01	6.79E-01	6.47E-01	5.55E-01	6.44E-01	7.07E-01
275	6-Methyl-3,5-heptadiene-2-one	Ketones	1604-28-0	3.59E-01	4.32E-01	3.62E-01	2.45E-01	2.39E-01	2.13E-01	1.60E-01	1.62E-01	1.58E-01	3.07E-01	2.82E-01	3.24E-01
276	6-Methyl-6-(5-methylfuran-2-yl)heptan-2-one	Ketones	50464-95-4	5.28E-02	6.56E-02	3.14E-02	4.45E-02	3.89E-02	3.63E-02	2.21E-02	2.55E-02	1.75E-02	5.29E-02	8.41E-02	3.60E-02
277	6-Tridecene	Hydrocarbons	24949-38-0	3.23E-03	3.23E-03	3.41E-03	3.47E-02	1.99E-02	4.65E-03	3.54E-02	4.66E-03	3.02E-02	2.79E-02	4.71E-03	3.68E-02
278	6-ethyl-2-methyl-Octane	Hydrocarbons	62016-19-7	4.38E-01	4.99E-01	4.35E-01	9.97E-02	8.33E-02	8.32E-02	8.96E-02	1.06E-01	8.71E-02	1.30E-01	1.35E-01	1.40E-01

279	6-hydroxy-2-Hexanone	Ketones	21856-89-3	1.28E-01	1.44E-01	1.27E-01	3.32E-02	3.01E-02	2.90E-02	2.77E-02	3.44E-02	2.27E-02	4.40E-02	4.41E-02	4.37E-02
280	6-methyl-2,4-Heptanedione	Ketones	3002-23-1	2.14E+00	2.61E+00	2.43E+00	1.30E+00	1.16E+00	1.01E+00	1.09E+00	1.41E+00	1.24E+00	1.16E+00	1.14E+00	1.12E+00
281	6-methyl-2-Heptanone	Ketones	928-68-7	5.25E-03	5.83E-03	4.63E-03	2.34E-03	2.27E-03	2.09E-03	3.24E-03	4.20E-04	3.64E-04	2.93E-03	3.52E-03	3.92E-03
282	6-methyl-3-Heptanone	Ketones	624-42-0	4.11E-03	4.95E-03	4.55E-03	3.28E-03	2.62E-03	2.30E-03	3.71E-03	3.51E-03	3.03E-03	3.05E-03	4.66E-04	4.87E-04
283	6-methyl-7-Oxa-8-azabicyclo[4.2.1]non-8-ene	Heterocyclic compounds	1000362-10-7	5.63E-01	6.56E-01	5.74E-01	5.12E-01	4.69E-01	4.22E-01	3.93E-01	4.16E-01	3.98E-01	6.28E-01	6.02E-01	6.61E-01
284	6Z-2,5,5,10-Tetramethyl-undeca-2,6,9-trien-8-one	Terpenoids	1000140-20-7	1.58E-04	1.58E-04	8.60E-04	2.77E-03	1.26E-03	1.13E-03	1.91E-03	1.59E-03	1.65E-03	2.19E-03	2.78E-03	2.98E-03
285	7-Octenoic acid	Acids	18719-24-9	4.55E-01	5.30E-01	5.58E-01	3.04E-01	2.91E-01	2.17E-01	2.40E-01	3.03E-01	3.40E-01	2.91E-01	2.73E-01	3.30E-01
286	7-Octylidenebicyclo[4.1.0]heptane	Terpenoids	82253-11-0	1.41E-02	1.33E-02	1.05E-02	1.05E-02	6.87E-03	5.24E-03	1.80E-02	1.78E-02	1.75E-02	1.49E-02	1.86E-02	2.20E-02
287	7-Oxabicyclo[4.1.0]heptane, 1-methyl-4-(1-methylethenyl)-	Terpenoids	1195-92-2	1.24E+01	1.49E+01	1.38E+01	7.41E+00	6.60E+00	5.91E+00	6.92E+00	8.79E+00	7.71E+00	6.53E+00	6.54E+00	6.51E+00
288	7-methyl-1-Undecene	Hydrocarbons	74630-42-5	5.14E-01	6.00E-01	5.88E-01	2.11E-01	1.77E-01	1.57E-01	1.37E-01	1.70E-01	1.92E-01	1.37E-01	1.23E-01	1.46E-01
289	7-methyl-1H-Indole	Heterocyclic compounds	933-67-5	2.80E-01	3.07E-01	2.51E-01	4.07E-02	2.43E-02	2.47E-02	3.91E-02	4.09E-02	3.59E-02	3.05E-02	3.56E-02	3.89E-02
290	7-methyl-5-Octen-4-one	Ketones	32064-78-1	5.61E-01	6.36E-01	5.52E-01	1.30E-01	1.11E-01	1.11E-01	1.22E-01	1.34E-01	1.12E-01	1.64E-01	1.71E-01	1.79E-01
291	Acetic acid, phenylmethyl ester	Esters	140-11-4	3.46E+00	3.80E+00	4.01E+00	2.17E+00	2.10E+00	1.54E+00	1.74E+00	2.20E+00	2.51E+00	2.11E+00	1.94E+00	2.33E+00
292	Acetophenone	Ketones	98-86-2	1.37E-01	1.39E-01	1.12E-01	1.04E-01	8.14E-02	7.61E-02	8.89E-02	1.00E-01	1.09E-01	8.47E-02	8.93E-02	9.35E-02
293	Acetophenone, 4'-hydroxy-	Ketones	99-93-4	2.39E+01	2.24E+01	2.22E+01	1.96E+01	1.67E+01	1.39E+01	1.56E+01	1.84E+01	1.73E+01	1.61E+01	1.83E+01	2.10E+01
294	Acetyl valeryl	Ketones	96-04-8	6.22E-01	6.38E-01	6.03E-01	7.54E-02	6.98E-02	6.87E-02	7.16E-02	8.60E-02	6.71E-02	1.03E-01	9.77E-02	9.29E-02
295	Acetylpyrazine	Heterocyclic compounds	22047-25-2	7.00E-02	7.00E-02	7.38E-02	7.10E-01	6.01E-01	5.81E-01	5.07E-01	5.65E-01	5.20E-01	7.98E-01	8.35E-01	9.29E-01
296	Allylidenecyclohexane	Hydrocarbons	5664-10-8	9.98E-02	1.07E-01	1.10E-01	8.82E-02	7.75E-02	7.54E-02	9.87E-02	1.17E-01	9.83E-02	7.62E-02	8.11E-02	8.46E-02

297	BenzAldehyde, 2,4-dimethyl-	Aldehydes	15764-16-6	6.68E-02	9.50E-02	6.40E-02	7.20E-02	6.33E-02	5.00E-02	4.20E-02	7.08E-02	6.59E-02	4.16E-02	4.33E-02	5.69E-02
298	BenzAldehyde, 4-methyl-	Aldehydes	104-87-0	2.46E-01	2.49E-01	2.31E-01	1.80E-01	1.61E-01	1.49E-01	1.56E-01	1.77E-01	2.13E-01	1.46E-01	1.53E-01	1.61E-01
299	Benzaldehyde, 4-pentyl-	Aldehydes	6853-57-2	3.03E-01	3.42E-01	2.12E-01	1.90E-01	1.84E-01	1.40E-01	2.66E-01	3.04E-01	2.30E-01	4.25E-01	5.06E-01	3.53E-01
300	BenzenAmine, 2,4-dimethyl-	Amines	95-68-1	5.22E-02	6.67E-02	4.61E-02	8.64E-02	7.39E-02	7.03E-02	8.06E-02	8.48E-02	9.05E-02	5.51E-02	7.08E-02	6.76E-02
301	BenzenAmine, N,N-dimethyl-	Amines	121-69-7	4.45E-02	4.93E-02	4.84E-02	2.80E-02	2.51E-02	2.29E-02	3.36E-02	3.74E-02	3.23E-02	2.32E-02	2.47E-02	2.46E-02
302	Benzene, (1-methyl-2-propynyl)-	Aromatics	4544-28-9	1.43E+00	1.63E+00	1.46E+00	1.26E+00	1.13E+00	9.95E-01	1.22E+00	1.29E+00	1.21E+00	1.01E+00	1.03E+00	1.02E+00
303	Benzene, (1-methylethyl)-	Aromatics	98-82-8	2.47E-02	2.72E-02	2.95E-02	1.60E-02	1.34E-02	1.32E-02	1.83E-02	2.06E-02	1.72E-02	2.31E-03	2.68E-03	2.80E-03
304	Benzene, 1,2-diethyl-3,4,5,6-tetramethyl-	Aromatics	33884-69-4	6.39E-05	1.47E-03	7.99E-04	1.22E-03	3.94E-04	8.84E-04	1.01E-03	1.31E-03	7.99E-05	7.77E-04	1.76E-03	2.13E-03
305	Benzene, 1,3-dimethyl-	Aromatics	108-38-3	2.03E-02	2.33E-02	1.86E-02	6.73E-02	5.47E-02	5.40E-02	6.85E-02	6.02E-02	5.27E-02	6.49E-02	7.52E-02	6.46E-02
306	Benzene, 1,4-diethyl-	Aromatics	105-05-5	1.23E-01	1.50E-01	1.29E-01	1.37E-01	1.21E-01	1.04E-01	9.57E-02	1.02E-01	1.03E-01	1.79E-01	1.68E-01	2.06E-01
307	Benzene, 1,4-dimethyl-2,5-bis(1-methylethyl)-	Aromatics	10375-96-9	4.47E-03	3.79E-03	3.11E-03	2.44E-03	1.29E-03	1.60E-03	1.58E-03	1.37E-03	1.68E-03	1.63E-03	1.86E-03	2.20E-03
308	Benzene, 1-(1,5-dimethyl-4-hexenyl)-4-methyl-	Terpenoids	644-30-4	1.17E-01	1.07E-01	7.86E-02	8.23E-02	6.52E-02	4.70E-02	1.79E-01	1.77E-01	1.57E-01	1.53E-01	1.83E-01	1.82E-01
309	Benzene, 1-ethenyl-4-ethyl-	Aromatics	3454-07-7	6.22E+00	8.39E+00	7.27E+00	5.37E+00	4.35E+00	4.03E+00	5.83E+00	5.41E+00	5.64E+00	4.05E+00	3.97E+00	4.33E+00
310	Benzene, 1-ethyl-2-methyl-	Aromatics	611-14-3	8.24E-01	1.59E+00	9.77E-01	6.54E-01	1.76E+00	4.98E-01	5.74E-01	6.45E-01	8.73E-01	5.81E-01	6.09E-01	6.22E-01
311	Benzene, 1-ethyl-3-methyl-	Aromatics	620-14-4	8.24E-01	1.59E+00	9.77E-01	6.54E-01	1.76E+00	4.98E-01	5.74E-01	6.45E-01	8.73E-01	5.81E-01	6.09E-01	6.22E-01
312	Benzene, 1-methyl-4-(1-methylethenyl)-	Aromatics	1195-32-0	1.04E+01	1.10E+01	9.51E+00	7.00E+00	5.71E+00	6.37E+00	7.58E+00	7.06E+00	7.35E+00	5.24E+00	5.10E+00	5.58E+00
313	Benzene, 1-methyl-4-(1-methylpropyl)-	Aromatics	1595-16-0	2.12E-03	3.30E-03	2.77E-03	2.10E-03	2.75E-03	1.69E-03	2.50E-03	2.41E-03	2.49E-03	2.15E-03	1.34E-03	1.96E-03

314	Benzene, 2-ethyl-1,4-dimethyl-	Aromatics	1758-88-9	6.54E+00	7.25E+00	6.51E+00	4.35E+00	3.60E+00	3.23E+00	3.44E+00	4.24E+00	4.38E+00	3.33E+00	3.20E+00	3.71E+00
315	Benzene, n-butyl-	Aromatics	104-51-8	3.61E+00	3.95E+00	3.61E+00	2.92E+00	2.45E+00	2.19E+00	2.60E+00	2.66E+00	2.79E+00	2.83E+00	2.78E+00	3.10E+00
316	Benzeneacetic acid	Acids	103-82-2	3.71E+01	4.19E+01	4.82E+01	4.33E+01	3.56E+01	3.19E+01	3.63E+01	4.09E+01	3.88E+01	5.69E+01	5.23E+01	5.33E+01
317	Benzenemethanol, α-2-cyclohexen-1-yl-	Alcohols	5723-89-7	1.11E-02	9.97E-03	8.02E-03	2.43E-04	1.34E-03	1.17E-03	1.56E-03	1.61E-03	1.12E-03	2.09E-03	3.11E-03	2.53E-03
318	Benzothiazole	Heterocyclic compounds	95-16-9	5.42E-02	6.39E-02	5.31E-02	5.50E-02	4.72E-02	3.85E-02	4.53E-02	4.18E-02	3.75E-02	4.12E-02	4.20E-02	5.09E-02
319	Betazole	Heterocyclic compounds	105-20-4	1.13E-01	1.07E-01	9.09E-02	6.66E-02	3.25E-02	3.09E-02	5.50E-02	5.78E-02	5.92E-02	5.04E-02	5.78E-02	6.70E-02
320	Bicyclo[2.2.1]heptane, 7,7-dimethyl-2-methylene-	Terpenoids	471-84-1	3.14E-01	3.51E-01	3.36E-01	2.70E-01	2.59E-01	2.18E-01	3.43E-01	3.52E-01	2.97E-01	2.62E-01	2.77E-01	2.87E-01
321	Biphenyl	Aromatics	92-52-4	3.73E-03	3.10E-03	3.00E-03	2.70E-03	1.28E-03	1.00E-03	1.93E-03	2.22E-03	2.24E-03	1.34E-03	1.49E-03	1.68E-03
322	Butane, 1-isothiocyanato-	Esters	592-82-5	4.44E-03	4.38E-03	4.31E-03	3.61E-03	3.02E-03	3.33E-03	4.36E-03	5.44E-03	4.15E-03	3.26E-03	3.63E-03	3.82E-03
323	Butanoic acid, 3-methyl-, butyl ester	Esters	109-19-3	4.16E-02	5.23E-02	4.87E-02	5.29E-02	4.16E-02	3.89E-02	3.58E-02	4.42E-02	4.95E-02	8.04E-02	8.46E-02	9.69E-02
324	Camphene	Terpenoids	79-92-5	2.55E-01	2.94E-01	2.73E-01	2.48E-01	2.14E-01	2.03E-01	2.81E-01	3.34E-01	2.76E-01	2.13E-01	2.25E-01	2.34E-01
325	Carbamodithioic acid, diethyl-, methyl ester	Esters	686-07-7	6.66E-02	6.66E-02	7.02E-02	7.59E-01	4.70E-01	4.79E-01	7.30E-01	7.66E-01	6.79E-01	5.65E-01	6.27E-01	6.72E-01
326	Carbonic acid, nonyl prop-1-en-2-yl ester	Esters	1000382-53-9	1.74E-04	1.74E-04	1.83E-04	2.80E-03	1.81E-03	1.25E-03	5.89E-03	5.78E-03	4.81E-03	4.71E-03	5.78E-03	5.55E-03
327	Carveol	Terpenoids	99-48-9	5.07E+00	5.74E+00	4.78E+00	3.98E+00	3.34E+00	3.06E+00	3.24E+00	4.15E+00	4.07E+00	2.94E+00	3.03E+00	3.20E+00
328	Carvone	Terpenoids	99-49-0	2.80E+01	2.13E+01	2.29E+01	1.79E+01	1.63E+01	1.32E+01	1.45E+01	1.83E+01	1.95E+01	1.57E+01	1.50E+01	1.69E+01
329	Caryophyllene	Terpenoids	87-44-5	9.90E-01	1.31E+00	1.02E+00	2.63E-01	1.61E-01	1.56E-01	2.81E-01	3.08E-01	2.47E-01	2.84E-01	2.97E-01	3.46E-01
330	Citral	Terpenoids	5392-40-5	3.01E+02	2.84E+02	2.97E+02	3.08E+02	2.40E+02	2.53E+02	2.71E+02	2.83E+02	2.57E+02	2.68E+02	3.06E+02	3.42E+02
331	Citronellol	Terpenoids	106-22-9	1.59E+01	1.63E+01	1.58E+01	1.06E+01	9.67E+00	8.16E+00	8.70E+00	1.10E+01	1.15E+01	9.54E+00	9.10E+00	1.18E+01
332	Cyclobutanecarboxylic acid, 3-methylbutyl ester	Esters	1000282-21-7	6.77E+00	8.28E+00	8.29E+00	3.98E+00	3.37E+00	2.98E+00	3.49E+00	4.45E+00	4.55E+00	5.10E+00	4.58E+00	5.49E+00
333	Cyclohexa-2,4-dienylmethanol	Alcohols	154916-94-6	3.25E-02	3.25E-02	3.43E-02	2.90E-01	2.45E-01	2.34E-01	3.13E-01	3.65E-01	3.16E-01	4.09E-02	4.74E-02	4.95E-02

334	Cyclohexanol, 1-methyl-4-(1-methylethylidene)-	Terpenoids	586-81-2	2.08E+01	2.05E+01	1.95E+01	1.27E+01	1.22E+01	9.36E+00	1.04E+01	1.31E+01	1.40E+01	1.07E+01	1.03E+01	1.44E+01
335	Cyclohexanone, 2-isobutyl-	Ketones	4668-64-8	1.97E+01	1.56E+01	1.52E+01	1.19E+01	1.34E+01	1.02E+01	9.02E+00	1.18E+01	1.31E+01	1.16E+01	1.10E+01	1.16E+01
336	Cyclohexanone, 2-methyl-5-(1-methylethenyl)-, trans-	Terpenoids	5948-04-9	3.26E+01	3.56E+01	3.46E+01	3.01E+01	2.60E+01	2.34E+01	2.46E+01	3.03E+01	3.13E+01	2.76E+01	2.70E+01	2.79E+01
337	Cyclohexanone, 5-methyl-2-(1-methylethyl)-	Terpenoids	10458-14-7	3.05E-01	2.61E-01	2.24E-01	2.24E-01	1.83E-01	1.73E-01	3.42E-01	3.98E-01	3.93E-01	6.22E-01	6.80E-01	7.43E-01
338	Cyclohexene, 2-ethenyl-1,3,3-trimethyl-	Hydrocarbons	5293-90-3	1.36E+00	1.54E+00	1.43E+00	6.92E-01	5.58E-01	5.61E-01	9.43E-01	1.11E+00	1.25E+00	7.81E-01	7.82E-01	8.84E-01
339	Cyclohexene, 4-methyl-1-(1-methylethenyl)-	Hydrocarbons	586-67-4	4.28E-01	7.01E-01	3.66E-01	2.14E-01	1.84E-01	1.56E-01	1.90E-01	1.90E-01	1.91E-01	2.00E-01	2.11E-01	2.13E-01
340	Cyclohexylmethyl formate	Esters	2888-49-5	2.56E+00	3.19E+00	3.18E+00	1.46E+00	1.24E+00	1.08E+00	1.26E+00	1.63E+00	1.66E+00	1.43E+00	1.89E+00	2.16E+00
341	Cyclopentane, nonyl-	Hydrocarbons	2882-98-6	3.80E-03	3.80E-03	4.00E-03	3.88E-02	2.87E-02	2.73E-02	5.26E-02	5.15E-02	4.36E-02	6.51E-02	7.62E-02	6.14E-02
342	Cyclopentanepropanoic acid, 3-oxo-, methyl ester	Esters	34399-78-5	1.30E-02	1.11E-02	9.25E-03	9.21E-03	5.37E-03	5.51E-03	9.09E-03	9.18E-03	8.70E-03	5.67E-03	6.90E-03	7.22E-03
343	D-Carvone	Terpenoids	2244-16-8	2.80E+01	2.13E+01	2.29E+01	1.79E+01	1.63E+01	1.32E+01	1.45E+01	1.83E+01	1.95E+01	1.57E+01	1.50E+01	1.69E+01
344	Decanal	Aldehydes	112-31-2	3.91E-02	3.69E-02	3.77E-02	2.64E-02	2.16E-02	2.15E-02	2.15E-02	2.56E-02	2.64E-02	2.27E-02	1.68E-02	2.44E-02
345	Decane, 3-chloro-	Halogenated hydrocarbons	1002-11-5	1.67E+00	2.22E+00	1.67E+00	1.48E+00	1.24E+00	1.11E+00	8.59E-01	1.56E+00	1.54E+00	1.08E+00	9.09E-01	1.15E+00
346	Decane, 3-ethyl-3-methyl-	Hydrocarbons	17312-66-2	7.90E-02	8.70E-02	8.11E-02	7.38E-02	6.48E-02	1.25E-02	6.71E-02	8.29E-02	8.18E-02	5.70E-02	1.27E-02	6.63E-02
347	Decanoic acid, methyl ester	Esters	110-42-9	6.82E-01	7.48E-01	7.43E-01	9.60E-01	6.56E-01	6.49E-01	7.19E-01	8.01E-01	7.32E-01	7.45E-01	8.48E-01	9.66E-01
348	DiSulfur compounds, methyl 2-propenyl	Sulfur compounds	2179-58-0	1.72E-03	1.77E-03	1.96E-03	1.23E-03	1.70E-04	9.95E-04	2.00E-04	1.28E-03	1.11E-03	1.74E-04	2.01E-04	2.10E-04
349	Diallyl Sulfur compounds	Sulfur compounds	592-88-1	9.13E-01	1.09E+00	1.00E+00	8.87E-01	7.99E-01	7.42E-01	8.96E-01	8.22E-01	7.46E-01	7.97E-01	8.39E-01	8.75E-01

350	Diethyltoluamide	Amines	134-62-3	7.06E-02	7.06E-02	7.44E-02	1.04E+00	8.40E-01	5.08E-01	2.99E+00	3.12E+00	2.40E+00	2.22E+00	2.52E+00	2.74E+00
351	Dimethyltrisulfide	Sulfur compounds	3658-80-8	1.33E-01	1.33E-01	1.40E-01	9.65E-01	1.01E+00	9.72E-01	8.34E-01	1.05E+00	1.10E+00	1.83E+00	1.61E+00	1.35E+00
352	Dodecanal	Aldehydes	112-54-9	8.29E-03	1.03E-02	8.42E-03	5.49E-03	3.31E-03	3.14E-03	3.97E-03	4.16E-03	3.24E-03	4.77E-03	6.11E-03	4.76E-03
353	Dodecane	Hydrocarbons	112-40-3	1.16E-01	1.56E-01	1.09E-01	9.97E-02	9.40E-02	8.65E-02	9.48E-02	1.10E-01	1.02E-01	8.75E-02	8.41E-02	1.02E-01
354	Dodecane, 2,6,10-trimethyl-	Hydrocarbons	3891-98-3	7.07E-04	1.19E-03	8.63E-04	7.78E-04	6.83E-04	6.75E-04	7.52E-04	5.32E-04	7.20E-04	1.17E-03	1.41E-03	1.04E-03
355	Dodecane, 2,6,11-trimethyl-	Hydrocarbons	31295-56-4	7.07E-04	1.19E-03	8.63E-04	7.78E-04	6.83E-04	6.75E-04	7.52E-04	5.32E-04	7.20E-04	1.17E-03	1.41E-03	1.04E-03
356	Dodecanenitrile	Nitrogen compounds	2437-25-4	1.07E-02	1.46E-02	7.94E-03	9.14E-03	7.12E-03	6.92E-03	5.24E-03	5.89E-03	4.60E-03	1.13E-02	1.44E-02	6.30E-03
357	E-2-Methyl-5-(furan-3-yl)-pent-1-en-3-one	Heterocyclic compounds	1000143-28-6	1.41E+02	1.31E+02	1.34E+02	1.14E+02	9.38E+01	8.55E+01	9.59E+01	1.07E+02	1.02E+02	9.88E+01	1.12E+02	1.27E+02
358	Estragole	Aromatics	140-67-0	3.08E+00	3.47E+00	2.91E+00	4.10E-01	3.33E-01	3.20E-01	4.67E-01	5.57E-01	5.29E-01	3.55E-01	3.55E-01	3.90E-01
359	Ethanol, 2-methoxyphenyl-	Alcohols	72403-22-6	1.11E+00	1.38E+00	1.16E+00	1.09E+00	9.13E-01	7.72E-01	8.95E-01	1.07E+00	1.08E+00	7.21E-01	7.31E-01	8.45E-01
360	Ethanol, 2-phenoxy-	Alcohols	122-99-6	5.53E-01	6.41E-01	5.63E-01	4.81E-01	3.44E-01	3.03E-01	3.97E-01	4.33E-01	4.69E-01	3.68E-01	3.71E-01	4.16E-01
361	Ethanone, 1-(1H-pyrazol-4-yl)-	Ketones	25016-16-4	2.49E+01	3.03E+01	3.04E+01	1.53E+01	1.30E+01	1.03E+01	1.21E+01	1.51E+01	1.53E+01	1.32E+01	1.23E+01	1.46E+01
362	Ethanone, 1-(2-methyl-1-cyclopenten-1-yl)-	Ketones	3168-90-9	3.07E+00	3.84E+00	3.05E+00	7.13E-01	6.00E-01	6.79E-01	1.37E-01	1.36E-01	1.18E-01	9.71E-01	1.00E+00	1.06E+00
363	Ethanone, 1-(2-methylphenyl)-	Ketones	577-16-2	1.48E+00	1.71E+00	1.64E+00	1.02E+00	8.06E-01	7.04E-01	8.35E-01	1.04E+00	1.16E+00	9.03E-01	8.61E-01	9.70E-01
364	Ethanone, 1-(3-hydroxyphenyl)-	Ketones	121-71-1	2.39E+01	2.24E+01	2.22E+01	1.96E+01	1.67E+01	1.39E+01	1.56E+01	1.84E+01	1.73E+01	1.61E+01	1.83E+01	2.10E+01
365	Ethyl 4-oxo-2-phenylpentanoate	Esters	1000427-11-2	2.68E-03	2.58E-03	2.53E-03	1.07E-03	6.18E-04	7.86E-04	8.83E-04	8.41E-04	1.11E-03	1.95E-03	2.26E-03	2.03E-03
366	Ethyl {[(ethylthio)carbonyl]thio}acetate	Esters	1000461-04-5	1.66E-02	1.50E-02	1.14E-02	2.29E-02	1.71E-02	1.20E-02	5.83E-02	5.53E-02	4.95E-02	4.93E-02	5.98E-02	6.15E-02
367	Ethylene glycol diglycidyl ether	Alcohols	2224-15-9	1.60E-03	2.81E-03	2.85E-03	4.24E-03	2.89E-03	2.47E-03	3.27E-03	4.45E-03	4.18E-03	5.00E-03	3.88E-03	4.48E-03

368	Eucalyptol	Terpenoids	470-82-6	2.50E+01	2.92E+01	2.81E+01	1.65E+01	1.46E+01	1.30E+01	1.39E+01	1.78E+01	1.56E+01	1.47E+01	1.45E+01	1.43E+01
369	Formamide, N-phenyl-	Aromatics	103-70-8	4.30E+00	4.75E+00	4.40E+00	2.53E+00	2.89E+00	2.10E+00	2.27E+00	3.00E+00	2.84E+00	2.45E+00	2.32E+00	2.65E+00
370	Formic acid, heptyl ester	Esters	112-23-2	9.20E-03	1.08E-02	9.90E-03	1.27E-02	1.38E-02	1.07E-02	9.37E-03	1.01E-02	1.38E-02	1.26E-02	1.16E-02	1.97E-03
371	Fumaric acid, ethyl 2-methylallyl ester	Esters	1000330-54-1	6.80E-03	6.79E-03	4.67E-03	5.86E-03	6.07E-03	4.48E-03	6.87E-03	6.28E-03	5.89E-03	6.74E-03	7.30E-03	7.84E-03
372	Furan, 2-(1-pentenyl)-, (E)-	Heterocyclic compounds	20992-69-2	2.77E-01	3.45E-01	3.24E-01	1.61E-01	1.35E-01	1.33E-01	1.71E-01	1.95E-01	2.07E-01	1.34E-01	1.40E-01	1.48E-01
373	Furan, 2-butyltetrahydro-	Heterocyclic compounds	1004-29-1	2.67E-03	3.13E-03	3.15E-03	7.98E-04	6.58E-04	7.68E-04	7.72E-04	7.69E-04	6.67E-04	6.71E-04	7.78E-04	8.12E-04
374	Furan, 2-hexyl-	Heterocyclic compounds	3777-70-6	2.33E+01	2.76E+01	2.77E+01	1.50E+01	1.26E+01	1.10E+01	1.28E+01	1.61E+01	1.67E+01	1.43E+01	1.33E+01	1.55E+01
375	Geranic acid	Terpenoids	459-80-3	1.41E-01	1.16E-01	9.91E-02	8.85E-02	4.75E-02	4.50E-02	6.80E-02	6.50E-02	6.05E-02	5.89E-02	6.91E-02	7.66E-02
376	Geraniol	Terpenoids	106-24-1	2.06E+01	1.61E+02	1.87E+02	1.82E+02	1.48E+02	1.48E+02	1.63E+02	1.77E+02	1.61E+02	1.60E+02	1.82E+02	2.05E+02
377	Geranyl acetate	Esters	105-87-3	3.69E+00	3.07E+00	2.56E+00	2.03E+00	1.18E+00	1.06E+00	1.90E+00	1.86E+00	1.72E+00	1.42E+00	1.71E+00	1.82E+00
378	Glycerin	Alcohols	56-81-5	6.84E-03	6.85E-03	7.21E-03	1.36E-01	1.20E-01	1.03E-01	9.90E-03	9.87E-03	8.56E-03	4.62E-02	5.49E-02	5.59E-02
379	Heptane, 3,3,4-trimethyl-	Hydrocarbons	20278-87-9	3.45E-04	3.45E-04	3.64E-04	5.16E-04	4.25E-04	4.96E-04	4.99E-04	4.97E-04	4.31E-04	2.17E-03	3.02E-03	3.41E-03
380	Heptanedioic acid	Acids	111-16-0	3.00E-03	3.06E-03	2.57E-03	1.74E-03	1.30E-03	1.10E-03	1.84E-03	2.15E-03	1.63E-03	1.57E-03	1.67E-03	2.13E-03
381	Heptanoic Acid	Acids	111-14-8	7.21E-03	7.63E-03	8.99E-03	7.46E-03	6.67E-03	5.56E-03	4.76E-03	6.08E-03	5.54E-03	7.81E-03	6.06E-03	8.07E-03
382	Heptanoic acid, ethyl ester	Esters	106-30-9	2.89E-02	3.16E-02	1.98E-02	5.47E-02	4.41E-02	4.26E-02	8.37E-02	8.72E-02	8.72E-02	5.06E-02	4.96E-02	5.50E-02
383	Heptanoic acid, methyl ester	Esters	106-73-0	6.86E-02	8.16E-02	7.58E-02	2.35E-02	2.27E-02	1.86E-02	7.83E-02	9.76E-02	8.82E-02	1.65E-02	1.44E-02	1.47E-02
384	Hexadecane	Hydrocarbons	544-76-3	4.08E-03	7.77E-03	7.66E-03	7.18E-03	4.09E-03	5.87E-03	7.19E-03	8.31E-03	3.94E-03	5.98E-03	8.85E-03	4.86E-03
385	Hexanoic Acid	Acids	142-62-1	2.85E-02	3.72E-02	2.83E-02	7.68E-03	9.02E-03	8.23E-03	7.55E-03	8.55E-03	6.24E-03	8.17E-03	2.23E-02	1.52E-03
386	Hexanoic acid, butyl ester	Esters	626-82-4	3.49E+00	3.39E+00	3.50E+00	2.88E+00	2.56E+00	2.26E+00	2.46E+00	3.00E+00	3.01E+00	2.36E+00	2.35E+00	2.58E+00
387	Hexanoic acid, ethyl ester	Esters	123-66-0	1.88E-02	1.88E-02	1.98E-02	1.62E-01	1.38E-01	1.35E-01	2.27E-01	2.56E-01	2.10E-01	1.82E-01	1.96E-01	2.05E-01
388	Hexanoic acid, propyl ester	Esters	626-77-7	7.28E-03	1.37E-03	1.44E-03	1.11E-02	1.17E-02	1.13E-02	9.88E-03	1.26E-02	1.05E-02	1.63E-02	1.22E-02	1.51E-02
389	Hexathiane	Sulfur compounds	13798-23-7	2.88E-04	2.88E-04	3.03E-04	3.45E-03	1.91E-03	4.14E-04	3.35E-03	4.15E-04	2.25E-03	2.08E-03	4.86E-03	2.19E-03
390	Humulene	Terpenoids	6753-98-6	1.21E-03	1.21E-03	1.28E-03	9.08E-03	1.50E-03	1.75E-03	1.76E-03	1.75E-03	1.52E-03	1.59E-02	1.87E-02	2.07E-02
391	Hydrazinecarboxylic acid, ethyl ester	Esters	4114-31-2	3.18E-03	2.23E-04	2.35E-04	2.08E-03	1.49E-03	1.61E-03	2.19E-03	2.81E-03	1.93E-03	1.78E-03	1.69E-03	3.40E-04

392	Indole	Heterocyclic compounds	120-72-9	1.31E-02	1.31E-02	1.38E-02	1.22E+00	5.83E-01	5.94E-01	1.06E-01	1.05E-01	8.18E-02	5.99E-01	6.65E-01	8.06E-01
393	Indole, 3-methyl-	Heterocyclic compounds	83-34-1	7.02E-03	6.05E-03	5.33E-03	4.28E-03	1.86E-03	2.03E-03	3.98E-03	3.89E-03	3.94E-03	3.36E-03	4.80E-03	4.99E-03
394	Iridomyrmecin	Terpenoids	485-43-8	7.57E-02	8.32E-02	5.96E-02	3.64E-02	2.51E-02	2.35E-02	3.32E-02	3.62E-02	3.08E-02	3.99E-02	4.08E-02	3.24E-02
395	Isobornyl acetate	Esters	125-12-2	7.52E+01	6.91E+01	6.91E+01	6.76E+01	4.69E+01	4.54E+01	4.92E+01	5.54E+01	4.99E+01	5.12E+01	5.79E+01	6.64E+01
396	Isophorone	Ketones	78-59-1	2.61E-02	3.92E-02	3.05E-02	2.76E-02	2.23E-02	2.10E-02	4.23E-03	4.21E-03	3.66E-03	2.56E-02	2.39E-02	2.91E-02
397	IsophthalAldehyde	Aldehydes	626-19-7	1.44E+01	1.51E+01	1.44E+01	2.01E+01	1.35E+01	1.27E+01	1.34E+01	1.56E+01	1.39E+01	1.41E+01	1.59E+01	1.83E+01
398	Isovaleric acid, 3-methylbutyl-2 ester	Esters	1000360-64-9	6.28E+00	6.95E+00	6.30E+00	1.60E+00	1.34E+00	1.32E+00	1.45E+00	1.72E+00	1.43E+00	2.12E+00	2.18E+00	2.21E+00
399	L-α-Terpineol	Terpenoids	10482-56-1	6.96E+00	7.41E+00	6.54E+00	3.54E+00	2.98E+00	2.69E+00	2.63E+00	3.25E+00	3.35E+00	3.48E+00	3.48E+00	3.63E+00
400	Levomenthol	Terpenoids	2216-51-5	4.37E-01	5.16E-01	5.50E-01	3.12E-01	2.95E-01	2.25E-01	2.54E-01	3.13E-01	3.44E-01	2.88E-01	2.75E-01	3.22E-01
401	Linalool	Terpenoids	78-70-6	1.21E+01	1.41E+01	1.25E+01	1.20E+01	1.02E+01	8.96E+00	9.21E+00	1.00E+01	9.51E+00	1.43E+01	1.32E+01	1.59E+01
402	Maleic hydrazide	Nitrogen compounds	123-33-1	1.77E+00	2.18E+00	2.00E+00	1.04E+00	9.31E-01	8.44E-01	9.02E-01	1.14E+00	1.01E+00	9.32E-01	9.27E-01	9.41E-01
403	Mequinol	Alcohols	150-76-5	1.19E+00	1.38E+00	1.18E+00	1.16E+00	1.00E+00	8.85E-01	8.27E-01	8.99E-01	8.85E-01	1.45E+00	1.35E+00	1.55E+00
404	Methyl 5-hydroxymethyl-4-imidazolecarboxylate	Esters	82032-43-7	5.08E-03	3.74E-03	3.69E-03	3.53E-03	1.57E-03	1.85E-03	2.74E-03	3.15E-03	2.34E-03	2.35E-03	2.43E-03	2.98E-03
405	Methyl trans-2-(3-cyclopropyl-7-norcaranyl)acetate	Esters	1000223-15-6	1.87E+00	1.82E+00	1.47E+00	8.27E-01	5.06E-01	4.90E-01	7.19E-01	8.09E-01	6.78E-01	6.79E-01	7.87E-01	8.36E-01
406	N,N'-Diacetylethylenediamine	Amines	871-78-3	5.35E-03	5.75E-03	4.45E-03	6.62E-03	3.64E-03	4.28E-03	1.28E-02	1.07E-02	7.52E-03	5.88E-03	5.47E-03	7.23E-03
407	N,N-Dimethyl-3-benzyloxypropylamine	Amines	71126-69-7	1.57E-02	1.52E-02	1.20E-02	1.35E-02	8.58E-03	7.87E-03	1.19E-02	1.34E-02	1.16E-02	1.08E-02	1.30E-02	1.33E-02
408	N-(2-Pyridinylmethyl)-1-butanamine, N-acetyl-	Heterocyclic compounds	1000475-32-2	9.47E-03	9.64E-03	6.77E-03	1.25E-03	1.58E-03	2.06E-03	2.56E-03	3.90E-03	1.85E-03	2.74E-03	4.16E-03	3.78E-03
409	N-(n-Butoxymethyl)acrylamide	Amines	1852-16-0	3.50E+01	3.15E+01	3.15E+01	2.85E+01	1.99E+01	1.97E+01	2.14E+01	2.41E+01	2.20E+01	2.23E+01	2.53E+01	2.89E+01

410	N-ethyl-2-Furancarboxamide	Heterocyclic compounds	1000407-23-8	4.98E-01	4.77E-01	4.53E-01	4.07E-01	3.70E-01	2.98E-01	3.08E-01	3.59E-01	3.30E-01	3.70E-01	4.22E-01	4.69E-01
411	N-ethyl-Cyclopentanamine	Amines	45592-46-9	2.56E-01	2.88E-01	2.75E-01	3.12E-02	2.58E-02	3.01E-02	1.93E-01	1.71E-01	1.31E-01	1.92E-01	2.08E-01	2.16E-01
412	N-propyl-Benzamide	Amines	10546-70-0	2.74E-01	3.01E-01	1.92E-01	2.73E-01	2.31E-01	1.97E-01	4.93E-01	5.60E-01	4.31E-01	5.78E-01	6.88E-01	5.75E-01
413	N1-(4-fluorobenzyl)-N2,N2-dimethyl-1,2-ethanediamine	Amines	2714-80-9	8.67E-01	8.54E-01	6.84E-01	4.13E-01	2.45E-01	2.33E-01	3.38E-01	3.95E-01	3.24E-01	3.22E-01	3.80E-01	4.02E-01
414	Naphthalene	Aromatics	91-20-3	1.59E-01	1.75E-01	1.54E-01	9.68E-02	9.15E-02	8.06E-02	1.20E-01	1.32E-01	1.23E-01	9.55E-02	1.02E-01	1.08E-01
415	Nonanal	Aldehydes	124-19-6	3.20E-01	3.73E-01	3.34E-01	3.45E-01	3.05E-01	7.48E-02	2.47E-01	2.77E-01	2.43E-01	3.80E-01	3.61E-01	4.12E-01
416	Nonane, 2,2,4,4,6,8,8-heptamethyl-	Hydrocarbons	4390-04-9	7.65E-02	8.99E-02	6.67E-02	1.65E-01	1.01E-01	1.00E-01	1.29E-01	1.15E-01	1.03E-01	1.24E-01	1.57E-01	1.53E-01
417	Nonane, 2-methyl-	Hydrocarbons	871-83-0	3.56E-04	5.58E-04	5.50E-04	1.05E-04	1.93E-03	1.01E-04	5.09E-04	5.47E-04	4.92E-04	4.60E-04	8.23E-04	6.40E-04
418	Nonaneperoxoic acid, 1,1-dimethylethyl ester	Esters	22913-02-6	8.32E-03	1.02E-02	6.06E-03	1.33E-02	9.19E-03	9.00E-03	9.77E-03	9.15E-03	7.49E-03	1.72E-02	1.83E-02	1.41E-02
419	Nonanoic acid, methyl ester	Esters	1731-84-6	1.62E-02	1.75E-02	1.52E-02	2.22E-02	1.69E-02	1.37E-02	2.18E-02	2.64E-02	2.55E-02	1.71E-02	1.77E-02	1.90E-02
420	Octane, 5-ethyl-2-methyl-	Hydrocarbons	62016-18-6	4.38E-01	4.99E-01	4.35E-01	9.97E-02	8.33E-02	8.32E-02	8.96E-02	1.06E-01	8.71E-02	1.30E-01	1.35E-01	1.40E-01
421	Octanoic Acid	Acids	124-07-2	1.08E-01	1.28E-01	9.97E-02	7.36E-02	1.20E-01	7.80E-02	1.35E-02	2.14E-02	2.80E-02	9.29E-02	9.93E-02	6.65E-02
422	Octanoic acid, ethyl ester	Esters	106-32-1	1.98E-03	1.98E-03	1.04E-02	1.91E-01	1.61E-01	1.50E-01	4.36E-01	5.39E-01	4.90E-01	1.32E-01	1.27E-01	1.45E-01
423	Oxalic acid, isobutyl neopentyl ester	Acids	1000309-72-6	5.88E-02	6.06E-02	4.66E-02	1.15E-01	7.37E-02	1.25E-01	1.66E-01	1.54E-01	1.41E-01	1.75E-01	2.05E-01	2.01E-01
424	Pentadecane	Hydrocarbons	629-62-9	9.87E-02	2.80E-01	1.44E-01	2.86E-01	1.35E-01	1.51E-01	3.32E-01	3.44E-01	2.23E-01	2.88E-01	3.78E-01	3.01E-01
425	Phenol	Phenols	108-95-2	2.10E+00	2.30E+00	2.23E+00	9.26E-01	8.40E-01	7.17E-01	1.30E+00	1.53E+00	1.27E+00	7.68E-01	9.32E-01	1.02E+00
426	Phenol, 2-butyl-	Aldehydes	3180-09-4	1.72E-03	1.73E-03	1.82E-03	1.84E-02	1.14E-02	1.24E-02	1.75E-02	1.40E-02	1.33E-02	1.09E-02	1.35E-02	1.39E-02
427	Phenol, 2-ethyl-	Phenols	90-00-6	2.24E-01	2.53E-01	2.13E-01	1.78E-01	1.41E-01	1.25E-01	1.47E-01	1.77E-01	1.95E-01	1.26E-01	1.38E-01	1.42E-01
428	Phenol, 3,5-dimethyl-	Phenols	108-68-9	4.00E-01	4.34E-01	3.68E-01	5.74E-01	4.64E-01	4.03E-01	2.58E-01	3.14E-01	3.31E-01	1.29E-01	1.30E-01	1.42E-01
429	Phenol, 3-methyl-	Aromatics	108-39-4	5.93E-02	5.70E-02	5.46E-02	2.56E-02	1.71E-02	1.66E-02	1.88E-02	2.65E-02	2.35E-02	1.72E-02	2.35E-02	2.00E-02
430	Phenol, 4-butyl-	Phenols	1638-22-8	1.72E-03	1.73E-03	1.82E-03	1.84E-02	1.14E-02	1.24E-02	1.75E-02	1.40E-02	1.33E-02	1.09E-02	1.35E-02	1.39E-02
431	Phenol, 4-ethyl-	Aromatics	123-07-9	2.24E-01	2.53E-01	2.13E-01	1.78E-01	1.41E-01	1.25E-01	1.47E-01	1.77E-01	1.95E-01	1.26E-01	1.38E-01	1.42E-01

432	Phenylethyl Alcohol	Alcohols	60-12-8	4.32E+00	5.58E+00	5.02E+00	2.73E+00	2.10E+00	1.91E+00	2.03E+00	3.19E+00	3.21E+00	1.49E+00	1.41E+00	1.63E+00
433	Phenylglyoxal	Aldehydes	1074-12-0	7.70E-01	9.92E-01	7.75E-01	9.09E-01	7.68E-01	7.00E-01	7.13E-01	9.58E-01	8.98E-01	6.64E-01	7.57E-01	7.51E-01
434	Piperidine	Heterocyclic compounds	110-89-4	1.57E-01	1.85E-01	1.71E-01	1.60E-01	1.39E-01	1.33E-01	1.84E-01	1.63E-01	1.48E-01	1.48E-01	1.56E-01	1.58E-01
435	Propanoic acid, 2-methyl-, 3-phenylpropyl ester	Esters	103-58-2	2.32E-02	2.90E-02	1.57E-02	3.73E-02	3.16E-02	2.70E-02	6.76E-02	7.55E-02	5.85E-02	7.35E-02	8.71E-02	7.65E-02
436	Propanoic acid, 3-ethoxy-, ethyl ester	Esters	763-69-9	1.57E-02	1.57E-02	1.66E-02	3.87E-01	3.66E-01	3.42E-01	2.27E-02	2.27E-02	1.97E-02	1.08E-01	1.17E-01	1.20E-01
437	Pulegone	Terpenoids	89-82-7	5.71E-01	6.77E+00	6.71E+00	5.95E+00	5.06E+00	4.11E+00	4.67E+00	5.54E+00	5.19E+00	4.74E+00	5.39E+00	6.16E+00
438	Pyrazine, 2,5-dimethyl-3-(3-methylbutyl)-	Heterocyclic compounds	18433-98-2	2.19E-02	1.98E-02	1.66E-02	2.18E-02	9.56E-03	1.06E-02	1.40E-02	1.14E-02	9.43E-03	9.14E-03	9.76E-03	9.77E-03
439	Pyrazine, 2-methyl-5-(1-methylethyl)-	Heterocyclic compounds	13925-05-8	7.38E+00	8.94E+00	8.31E+00	4.63E+00	4.04E+00	3.78E+00	4.49E+00	5.51E+00	4.84E+00	4.11E+00	4.19E+00	4.32E+00
440	Pyrazine, trimethyl-	Heterocyclic compounds	14667-55-1	7.24E-02	7.25E-02	7.64E-02	7.14E-01	6.07E-01	5.86E-01	5.24E-01	5.69E-01	5.26E-01	8.02E-01	8.43E-01	9.32E-01
441	Pyridine, 2-hexyl-	Heterocyclic compounds	1129-69-7	2.54E+01	2.34E+01	2.32E+01	2.22E+01	1.54E+01	1.49E+01	1.62E+01	1.82E+01	1.64E+01	1.69E+01	1.89E+01	2.19E+01
442	Pyrimidine, 4-butyl-3,4-dihydro-5-methyl-	Heterocyclic compounds	1000115-50-2	1.33E-02	1.25E-02	1.16E-02	5.33E-03	3.28E-03	3.80E-03	4.23E-03	7.10E-03	7.51E-03	6.65E-04	7.70E-04	8.05E-04
443	Quinazoline, 4-methyl-	Heterocyclic compounds	700-46-9	8.51E-05	1.39E-03	9.67E-04	2.25E-03	5.42E-04	1.67E-03	3.24E-03	3.04E-03	2.31E-03	5.67E-04	7.13E-04	6.48E-04
444	Quinoxaline, 2-methyl-	Heterocyclic compounds	7251-61-8	8.51E-05	1.39E-03	9.67E-04	2.25E-03	5.42E-04	1.67E-03	3.24E-03	3.04E-03	2.31E-03	5.67E-04	7.13E-04	6.48E-04
445	Sorbic acid vinyl ester	Esters	42739-26-4	2.90E-01	4.50E-01	2.99E-01	1.20E-01	9.87E-02	9.01E-02	6.95E-02	8.25E-02	5.34E-02	7.57E-02	7.57E-02	2.33E-02
446	Sulfurous acid, butyl pentyl ester	Esters	1000309-17-1	1.07E-02	1.64E-02	7.45E-03	9.69E-03	1.15E-02	1.02E-02	1.06E-02	1.08E-02	7.25E-03	1.97E-02	1.86E-02	9.75E-03
447	Sulfurous acid, isohexyl 2-pentyl ester	Acids	1000309-15-5	3.62E-02	5.82E-02	5.43E-02	5.31E-02	2.92E-02	4.21E-02	4.72E-02	6.04E-02	2.82E-02	4.35E-02	6.53E-02	4.27E-02
448	Terpinen-4-ol	Terpenoids	562-74-3	5.77E+00	6.44E+00	5.99E+00	3.48E+00	3.37E+00	2.66E+00	2.78E+00	3.46E+00	3.70E+00	3.31E+00	3.25E+00	3.66E+00

449	Tetracyclo[6.2.1.0(2,4).0(4,7)]undecane	Hydrocarbons	1000223-17-7	3.11E-01	3.40E-01	3.42E-01	2.03E-01	1.66E-01	1.63E-01	2.34E-01	2.60E-01	2.17E-01	1.50E-01	1.57E-01	1.55E-01
450	Tetradecane	Hydrocarbons	629-59-4	2.22E-01	2.67E-01	1.67E-01	3.21E-01	1.72E-01	1.56E-01	3.16E-01	2.88E-01	2.60E-01	2.27E-01	3.18E-01	2.84E-01
451	Tetradecane, 4-methyl-	Hydrocarbons	25117-24-2	4.20E-02	4.78E-02	2.98E-02	5.63E-02	3.59E-02	3.35E-02	4.17E-02	4.21E-02	3.24E-02	6.47E-02	7.74E-02	6.28E-02
452	Thiophene, 2-pentyl-	Heterocyclic compounds	4861-58-9	5.51E+00	5.58E+00	5.37E+00	3.90E+00	3.74E+00	2.91E+00	3.16E+00	4.07E+00	4.30E+00	3.31E+00	3.19E+00	3.53E+00
453	Triacetin	Esters	102-76-1	2.67E-04	2.67E-04	2.82E-04	3.37E-02	1.77E-02	1.76E-02	2.36E-03	1.93E-03	2.10E-03	1.16E-02	1.50E-02	1.63E-02
454	Tris-(hydroxymethyl)-phosphine oxide	Others	1067-12-5	3.08E-03	2.82E-03	2.27E-03	1.85E-03	1.10E-03	1.28E-03	1.71E-03	2.01E-03	1.77E-03	1.36E-03	1.72E-03	2.04E-03
455	Undecanal	Aldehydes	112-44-7	1.74E-03	3.22E-02	3.04E-02	2.28E-02	1.14E-02	1.25E-02	1.55E-02	1.48E-02	1.33E-02	1.31E-02	1.71E-02	1.62E-02
456	Undecane, 2-methyl-	Hydrocarbons	7045-71-8	2.72E-04	2.73E-04	2.87E-04	2.04E-03	1.81E-03	2.23E-03	3.30E-03	3.88E-03	2.99E-03	2.54E-03	3.54E-03	3.15E-03
457	Undecane, 4,4-dimethyl-	Hydrocarbons	17312-68-4	4.62E-01	5.94E-01	5.36E-01	5.30E-01	4.36E-01	3.77E-01	4.75E-01	5.79E-01	5.39E-01	3.88E-01	4.07E-01	4.59E-01
458	Undecane, 4-methyl-	Hydrocarbons	2980-69-0	4.97E-03	6.31E-03	7.90E-03	7.29E-03	7.61E-03	6.52E-03	6.39E-03	7.06E-03	6.83E-03	6.18E-03	5.80E-03	6.17E-03
459	Undecane, 6,6-dimethyl-	Hydrocarbons	17312-76-4	7.90E-02	8.70E-02	8.11E-02	7.38E-02	6.48E-02	1.25E-02	6.71E-02	8.29E-02	8.18E-02	5.70E-02	1.27E-02	6.63E-02
460	Vanillic acid	Phenols	121-34-6	9.61E-04	1.56E-03	7.64E-04	2.08E-03	1.15E-03	1.57E-03	1.26E-03	1.89E-03	1.44E-03	1.43E-03	2.05E-03	1.60E-03
461	Vinyl 10-undecenoate	Esters	5299-57-0	3.46E-03	4.46E-03	3.05E-03	2.13E-03	1.33E-03	1.41E-03	1.98E-03	2.30E-03	2.12E-03	1.76E-03	2.85E-04	2.08E-03
462	Ylangene	Terpenoids	14912-44-8	2.36E-01	2.97E-01	2.98E-01	2.56E-01	2.26E-01	1.80E-01	2.02E-01	2.01E-01	1.86E-01	1.76E-01	1.47E-01	2.06E-01
463	Z-2-Dodecenol	Alcohols	69064-36-4	4.17E-02	4.98E-02	3.00E-02	5.63E-02	3.60E-02	3.25E-02	4.30E-02	4.26E-02	3.18E-02	6.25E-02	7.32E-02	6.16E-02
464	cis-1,3-dichloro-Cyclopentane	Halogenated hydrocarbons	26688-51-7	5.53E-01	6.18E-01	5.93E-01	2.73E-01	2.30E-01	2.20E-01	3.31E-01	3.65E-01	3.13E-01	2.17E-01	2.33E-01	2.30E-01
465	cis-1-methyl-4-(1-methylethenyl)-Cyclohexane	Hydrocarbons	1879-07-8	2.39E-02	2.27E-02	2.24E-02	7.72E-02	6.38E-02	6.00E-02	6.32E-02	6.29E-02	6.83E-02	7.07E-02	7.21E-02	1.18E-01
466	cis-2,6-Dimethyl-2,6-octadiene	Hydrocarbons	2492-22-0	1.83E+01	2.04E+01	1.83E+01	6.18E-01	4.36E-01	4.37E-01	4.54E+00	5.49E+00	4.62E+00	5.70E+00	5.84E+00	5.98E+00

467	cis-2-(1,1-dimethylethyl)-Cyclohexanol, acetate	Esters	20298-69-5	1.99E-02	6.77E-02	1.89E-02	7.42E-02	4.32E-02	4.40E-02	5.90E-02	5.20E-02	4.43E-02	4.46E-02	5.33E-02	6.13E-02
468	cis-2-(2-Pentenyl)furan	Heterocyclic compounds	70424-13-4	2.77E-01	3.45E-01	3.24E-01	1.61E-01	1.35E-01	1.33E-01	1.71E-01	1.95E-01	2.07E-01	1.34E-01	1.40E-01	1.48E-01
469	cis-3-Decene	Hydrocarbons	19398-86-8	2.11E+00	2.58E+00	2.41E+00	1.37E+00	1.20E+00	1.12E+00	1.18E+00	1.45E+00	1.29E+00	1.20E+00	1.22E+00	1.24E+00
470	dl-Menthol	Terpenoids	89-78-1	3.22E-01	3.26E-01	3.73E-01	1.21E-01	8.28E-02	7.98E-02	1.31E-01	1.71E-01	1.74E-01	1.20E-01	1.07E-01	1.34E-01
471	heptyl-Benzene	Aromatics	1078-71-3	4.31E-02	4.57E-02	3.75E-02	9.19E-02	5.29E-02	4.86E-02	9.09E-02	9.61E-02	9.40E-02	7.89E-02	9.30E-02	1.01E-01
472	isocyanato-Cyclohexane	Esters	3173-53-3	1.64E+00	2.01E+00	1.58E+00	1.28E+00	1.07E+00	9.88E-01	1.05E+00	1.37E+00	1.28E+00	9.29E-01	1.01E+00	1.06E+00
473	isothiocyanato-Cyclohexane	Others	1122-82-3	3.75E+01	3.40E+01	3.46E+01	2.73E+01	2.20E+01	2.01E+01	2.30E+01	2.63E+01	2.47E+01	2.32E+01	2.63E+01	3.02E+01
474	isothiocyanato-Cyclopropane	Others	56601-42-4	2.33E-04	1.04E-02	2.45E-04	3.48E-04	2.87E-04	3.35E-04	1.86E-03	1.68E-03	2.91E-04	2.93E-04	3.39E-04	3.54E-04
475	m-Chloroaniline	Amines	108-42-9	1.22E-02	1.22E-02	1.29E-02	1.23E-01	1.03E-01	8.78E-02	2.15E-01	2.58E-01	2.49E-01	1.15E-01	1.08E-01	1.28E-01
476	methoxy-phenyl-_Oxime-	Nitrogen compounds	1000222-86-6	4.90E-02	4.90E-02	5.16E-02	5.77E-01	3.29E-01	3.52E-01	6.47E-01	6.04E-01	5.15E-01	4.38E-01	4.89E-01	5.26E-01
477	methyl-Carbamic acid,3-methylphenyl ester	Esters	1129-41-5	2.51E-01	2.38E-01	1.98E-01	1.58E-01	7.89E-02	7.24E-02	1.20E-01	1.28E-01	1.27E-01	1.13E-01	1.28E-01	1.42E-01
478	o-Toluidine	Amines	95-53-4	1.70E-01	2.07E-01	1.79E-01	1.78E-01	1.48E-01	1.36E-01	1.37E-01	1.39E-01	1.45E-01	2.18E-01	2.01E-01	2.46E-01
479	o-Xylene	Aromatics	95-47-6	2.03E-02	2.33E-02	1.86E-02	6.73E-02	5.47E-02	5.40E-02	6.85E-02	6.02E-02	5.27E-02	6.49E-02	7.52E-02	6.46E-02
480	octahydro-3-methyl-1H-Indole	Heterocyclic compounds	37865-94-4	3.86E+00	4.29E+00	4.00E+00	2.18E+00	2.51E+00	1.83E+00	2.00E+00	2.17E+00	2.45E+00	2.14E+00	1.99E+00	2.30E+00
481	octyl-Cyclohexane	Hydrocarbons	1795-15-9	8.27E-04	8.27E-04	8.71E-04	1.35E-02	6.74E-03	5.95E-03	1.01E-02	1.08E-02	9.46E-03	2.05E-02	2.63E-02	2.81E-02
482	p-Cresol	Aromatics	106-44-5	5.93E-02	5.70E-02	5.46E-02	2.56E-02	1.71E-02	1.66E-02	1.88E-02	2.65E-02	2.35E-02	1.72E-02	2.35E-02	2.00E-02
483	p-Xylene	Aromatics	106-42-3	2.03E-02	2.33E-02	1.86E-02	6.73E-02	5.47E-02	5.40E-02	6.85E-02	6.02E-02	5.27E-02	6.49E-02	7.52E-02	6.46E-02
484	para-Anisaldehyde diethyl acetal	Aromatics	1000430-95-0	3.81E-04	3.82E-04	4.02E-04	6.50E-03	3.32E-03	2.74E-03	4.47E-03	5.05E-03	4.52E-03	8.35E-03	1.16E-02	1.30E-02
485	propyl-Benzene	Aromatics	103-65-1	2.08E-01	2.34E-01	2.18E-01	1.29E-01	1.09E-01	1.10E-01	1.45E-01	1.69E-01	1.44E-01	1.09E-01	1.16E-01	1.21E-01
486	propyl-Cyclohexane	Hydrocarbons	1678-92-8	5.28E+00	5.90E+00	5.24E+00	1.09E+00	9.07E-01	8.99E-01	9.59E-01	1.15E+00	9.62E-01	1.44E+00	1.47E+00	1.54E+00

487	sec-Butyl propyl carbonate	Esters	1000372-80-7	3.23E-02	3.86E-02	3.33E-02	8.64E-02	7.34E-02	7.07E-02	1.14E-01	1.18E-01	9.80E-02	8.28E-02	9.96E-02	8.05E-02
488	tetraethyl-Urea	Nitrogen compounds	1187-03-7	2.45E-01	2.93E-01	2.05E-01	1.37E-01	2.03E-01	1.33E-01	3.47E-02	3.71E-02	4.21E-02	1.35E-01	1.89E-01	1.14E-01
489	tetrahydro-2,2-dimethyl-5-(1-methyl-1-propenyl)-Furan	Heterocyclic compounds	7416-35-5	1.27E-02	1.45E-02	1.33E-02	7.99E-03	6.64E-03	6.34E-03	5.60E-03	6.60E-03	5.46E-03	1.03E-02	1.15E-02	1.60E-02
490	trans-β-Ocimene	Terpenoids	3779-61-1	1.52E+01	1.69E+01	1.64E+01	8.92E+00	7.36E+00	7.07E+00	1.01E+01	1.17E+01	9.71E+00	7.33E+00	7.94E+00	7.96E+00
491	trans-1,3-dichloro-Cyclopentane	Halogenated hydrocarbons	26688-50-6	5.53E-01	6.18E-01	5.93E-01	2.73E-01	2.30E-01	2.20E-01	3.31E-01	3.65E-01	3.13E-01	2.17E-01	2.33E-01	2.30E-01
492	trans-3-methyl-6-(1-methylethyl)-Cyclohexene	Hydrocarbons	1124-26-1	6.17E-02	7.09E-02	6.51E-02	5.22E-02	4.45E-02	4.17E-02	6.21E-02	6.99E-02	5.91E-02	4.67E-02	5.05E-02	5.03E-02
493	β-Ionone	Terpenoids	14901-07-6	1.67E-01	2.09E-01	1.07E-01	1.23E-01	1.12E-01	1.08E-01	1.12E-01	1.10E-01	7.64E-02	2.17E-01	2.68E-01	1.38E-01
