# Supplementary material for: Interaction and Flavor Metabolic Function of Microbiota During Fermentation of Pigskin Through Bioaugmentation with Latilactobacillus sakei
Source: Molecules. 2026 Jun 1;31(11):1889. doi: 10.3390/molecules31111889 (PMC13258469; doi:10.3390/molecules31111889)
Supplement: Supplementary file 1 [file molecules-31-01889-s001.zip › Tables S2¿CS6.pdf]

Table S2. The differentially changed volatile flavor compounds (DCVFCs) between F5 and F0.

DCVFCs	Class	Fold change	*P*-value
6-methyl-2-Heptanone	Ketones	0.43	1.06E-03
7-Oxabicyclo[4.1.0]heptane, 1-methyl-4-(1-methylethenyl)-	Terpenoids	0.48	1.11E-03
2,6-Dimethyl-1,3,5,7-octatetraene, E,E-	Terpenoids	0.47	3.01E-04
2,2-dimethyl-Cyclopentanone	Ketones	0.18	1.72E-04
1-(2-methyl-2-cyclopenten-1-yl)-Ethanone	Ketones	0.23	6.14E-05
α-Ionone	Terpenoids	0.13	1.44E-03
α-Cubebene	Terpenoids	0.15	5.21E-04
Caryophyllene	Terpenoids	0.17	1.06E-03
Iridomyrmecin	Terpenoids	0.39	5.25E-03
β-Pinene	Terpenoids	0.47	2.50E-04
β-Ocimene	Terpenoids	0.47	3.11E-04
trans-β-Ocimene	Terpenoids	0.48	3.93E-04
Phenol	Phenols	0.37	8.11E-05
4-Hexen-1-ol, acetate	Others	0.21	2.82E-04
6-hydroxy-2-Hexanone	Ketones	0.23	5.48E-05
3,4-bis(1,1-dimethylethyl)-2,2,5,5-tetramethyl-Hexane	Hydrocarbons	0.04	8.70E-04
5-methyl-Hexanenitrile	Nitrogen compounds	0.20	2.09E-05
3-Ethylcyclopentanone	Ketones	0.10	8.86E-07
2-Octanone	Ketones	0.20	1.46E-05
Ethanone, 1-(2-methyl-1-cyclopenten-1-yl)-	Ketones	0.20	5.35E-04
Ethanone, 1-(1H-pyrazol-4-yl)-	Ketones	0.45	2.51E-03
4-Amino-4,5(1H)-dihydro-1,2,4-triazole-5-one	Ketones	0.48	4.45E-03
2,4-dimethyl-2,4-Heptadiene	Hydrocarbons	0.06	1.89E-04
β-Myrcene	Terpenoids	0.02	3.06E-06
α-Phellandrene 1	Terpenoids	0.47	2.50E-04
2-Buten-1-one, 1-(2,6,6-trimethyl-1,3-cyclohexadien-1-yl)-, (E)-	Terpenoids	0.48	2.75E-02
Acetyl valeryl	Ketones	0.11	7.45E-07
7-methyl-5-Octen-4-one	Ketones	0.20	7.00E-05
3,5-Octadien-2-one, (E,E)-	Ketones	0.42	1.45E-03
5,9-Undecadien-2-one, 6,10-dimethyl-	Ketones	0.50	4.20E-02
cis-2,6-Dimethyl-2,6-octadiene	Hydrocarbons	0.03	1.24E-05
1-ethyl-Cyclohexene	Hydrocarbons	0.15	1.30E-05
dl-Menthol	Terpenoids	0.28	3.09E-04
α-Farnesene	Terpenoids	0.37	7.44E-03
α-Muurolene	Terpenoids	0.37	4.63E-03
2-Butanone, 4-(2,6,6-trimethyl-1-cyclohexen-1-yl)-	Ketones	0.38	6.60E-03
1-(1-cyclohexen-1-yl)-Ethanone	Ketones	0.42	9.68E-04
5-Hepten-2-one, 6-methyl-	Ketones	0.19	3.42E-05
3,5-Octadien-2-one	Ketones	0.42	1.45E-03
2,6-Octadiene, 2,6-dimethyl-	Hydrocarbons	0.03	1.24E-05
2,6-Dimethyl-2-trans-6-octadiene	Terpenoids	0.03	1.24E-05
DiSulfur compounds, methyl 2-propenyl	Sulfur compounds	0.44	3.66E-02
3,5,9-Undecatrien-2-one, 6,10-dimethyl-	Ketones	0.33	2.57E-02
6-methyl-2,4-Heptanedione	Ketones	0.48	1.53E-03
L-α-Terpineol	Terpenoids	0.44	3.86E-04
α-Terpineol	Terpenoids	0.44	3.86E-04
1,3,6-Octatriene, 3,7-dimethyl-, (Z)-	Terpenoids	0.48	3.93E-04

Maleic hydrazide	Nitrogen compounds	0.47	1.36E-03
1-Decen-3-one	Ketones	0.49	1.21E-03
2,2-dihydroxy-1-phenyl-Ethanone	Ketones	0.32	2.44E-03
2,7-dimethyl-2,6-Octadiene	Hydrocarbons	0.03	1.24E-05
propyl-Cyclohexane	Hydrocarbons	0.18	3.51E-05
Octane, 5-ethyl-2-methyl-	Hydrocarbons	0.19	6.87E-05
6-ethyl-2-methyl-Octane	Hydrocarbons	0.19	6.87E-05
2-Methyl-1-nonene-3-yne	Hydrocarbons	0.23	3.24E-05
(1-methylethylidene)-Cyclohexane	Hydrocarbons	0.23	6.74E-05
5-methyl-Nonane	Hydrocarbons	0.25	1.68E-05
1,2,3-trimethoxy-Propane	Hydrocarbons	0.32	2.18E-04
7-methyl-1-Undecene	Hydrocarbons	0.32	2.45E-04
Cyclohexene, 4-methyl-1-(1-methylethenyl)-	Hydrocarbons	0.37	3.96E-02
1,5,5-trimethyl-3-methylene-Cyclohexene	Hydrocarbons	0.41	1.94E-03
1,5,5-Trimethyl-6-methylene-cyclohexene	Hydrocarbons	0.41	1.94E-03
Cyclohexene, 2-ethenyl-1,3,3-trimethyl-	Hydrocarbons	0.42	2.55E-04
1-Tetradecene	Hydrocarbons	0.42	1.50E-02
(E)-8-methyl-5-Undecene	Hydrocarbons	0.44	2.61E-03
1-methyl-3-(2-methyl-2-propenyl)-Cyclopentane	Hydrocarbons	0.46	5.33E-04
2,3-dimethyl-Heptane	Hydrocarbons	0.50	3.74E-03
2-methyl-5-(methylthio)-Furan	Heterocyclic compounds	0.06	8.76E-05
5-methyl-1H-Indole	Heterocyclic compounds	0.11	1.27E-04
7-methyl-1H-Indole	Heterocyclic compounds	0.11	1.27E-04
N-(2-Pyridinylmethyl)-1-butanamine, N-acetyl-	Heterocyclic compounds	0.19	1.88E-03
4(1H)-Pyridinone, 2,3-dihydro-1-methyl-	Heterocyclic compounds	0.19	5.91E-05
1-methyl-Piperazine	Heterocyclic compounds	0.20	2.21E-05
1-Tetrazol-2-ylethanone	Heterocyclic compounds	0.22	3.54E-04
2-Methyl-3-furanthiol	Heterocyclic compounds	0.23	2.83E-04
Furan, 2-butyltetrahydro-	Heterocyclic compounds	0.25	1.60E-04
1-Methylimidazole-5-carboxaldehyde	Heterocyclic compounds	0.26	2.44E-05
4H-1,2,4-Triazol-4-amine	Heterocyclic compounds	0.31	1.42E-04
Pyrimidine, 4-butyl-3,4-dihydro-5-methyl-	Heterocyclic compounds	0.33	4.51E-04
Betazole	Heterocyclic compounds	0.42	1.08E-02
6,8-Nonadien-2-one, 6-methyl-5-(1-methylethylidene)-	Heterocyclic compounds	0.43	1.15E-02
Indole, 3-methyl-	Heterocyclic compounds	0.44	2.08E-02
2-methyl-1H-Indole	Heterocyclic compounds	0.44	2.08E-02
Furan, 2-(1-pentenyl)-, (E)-	Heterocyclic compounds	0.45	1.44E-03
cis-2-(2-Pentenyl)furan	Heterocyclic compounds	0.45	1.44E-03
1,3-Benzodioxole	Heterocyclic compounds	0.46	9.73E-04
2,2'-Ethylidenebis(5-methylfuran)	Heterocyclic compounds	0.48	1.42E-02
2-cyano-Imidazole	Heterocyclic compounds	0.48	1.77E-03
2-Isobutyl-4-methylpyridine	Heterocyclic compounds	0.49	4.44E-03
Furan, 2-hexyl-	Heterocyclic compounds	0.49	2.00E-03
1-chloro-Dodecane	Halogenated hydrocarbons	0.15	2.33E-03
trans-1,3-dichloro-Cyclopentane	Halogenated hydrocarbons	0.41	1.55E-04
cis-1,3-dichloro-Cyclopentane	Halogenated hydrocarbons	0.41	1.55E-04
2-(4-bromobutyl)-5,5-dimethyl-1,3-Cyclopentadiene	Halogenated hydrocarbons	0.45	1.57E-02
Isovaleric acid, 3-methylbutyl-2 ester	Esters	0.22	2.82E-05
2,3,4-Trifluorobenzoic acid, cyclobutyl ester	Esters	0.24	4.14E-04

Heptanoic acid, methyl ester	Esters	0.29	1.87E-04
Sorbic acid vinyl ester	Esters	0.30	9.86E-03
Ethyl 4-oxo-2-phenylpentanoate	Esters	0.32	2.19E-04
Methyl trans-2-(3-cyclopropyl-7-norcaranyl)acetate	Esters	0.35	2.64E-03
1,3,5,7-Cyclooctatetraene	Esters	0.40	2.85E-04
Cyclohexylmethyl formate	Esters	0.42	1.89E-03
Cyclobutanecarboxylic acid, 3-methylbutyl ester	Esters	0.44	1.74E-03
Vinyl 10-undecenoate	Esters	0.44	1.43E-02
2-methyl-Butanoic acid,2-methyl-2-propenyl ester	Esters	0.44	4.00E-04
methyl-Carbamic acid,3-methylphenyl ester	Esters	0.45	1.67E-02
Geranyl acetate	Esters	0.46	1.97E-02
2-Butoxyethyl acetate	Esters	0.48	1.55E-03
Estragole	Aromatics	0.11	7.63E-05
2-propenyl-Benzene	Aromatics	0.22	4.42E-05
p-Cresol	Aromatics	0.35	3.21E-04
Phenol, 3-methyl-	Aromatics	0.35	3.21E-04
α-Methylstyrene	Aromatics	0.37	5.32E-04
Benzene, 1,4-dimethyl-2,5-bis(1-methylethyl)-	Aromatics	0.47	1.82E-02
N-ethyl-Cyclopentanamine	Amines	0.11	1.33E-05
3-methyl-Butanamide	Amines	0.20	2.37E-05
1,4-ButanediAmine	Amines	0.30	1.07E-04
1,3-Propanediamine, N-(1-methylethyl)-	Amines	0.31	6.86E-05
N1-(4-fluorobenzyl)-N2,N2-dimethyl-1,2-ethanediamine	Amines	0.37	3.66E-03
5H-Tetrazol-5-Amine	Amines	0.43	1.55E-03
2-ethyl-2-Hexenal	Aldehydes	0.18	6.30E-05
5-Hydroxymethylfurfural	Aldehydes	0.40	1.87E-03
Dodecanal	Aldehydes	0.44	7.29E-03
Benzenemethanol, α-2-cyclohexen-1-yl-	Alcohols	0.09	8.00E-04
1-Heptanol	Alcohols	0.15	2.27E-04
3-Octanol	Alcohols	0.21	2.84E-04
3-Cyclohexene-1-ethanol	Alcohols	0.28	1.59E-04
5,9-Undecadien-2-ol, 6,10-dimethyl-	Alcohols	0.37	2.93E-02
Phenylethyl Alcohol	Alcohols	0.45	3.47E-03
2-methyl-Decanoic acid	Acids	0.17	8.45E-04
Hexanoic Acid	Acids	0.27	1.47E-03
3-Methyl-1-adamantanecarboxylic acid	Acids	0.41	1.03E-02
Heptanedioic acid	Acids	0.48	3.58E-03
3-Methylbutan-2-yl (E)-2-methylbut-2-enoate	Acids	0.50	1.40E-03
Dimethyl triSulfur compounds	Sulfur compounds	11.80	4.60E-04
Phenol, 4-butyl-	Phenols	8.01	4.89E-03
1-iodo-Decane	Others	5.45	2.43E-03
methoxy-phenyl-_Oxime-	Nitrogen compounds	8.41	9.52E-03
2-methoxy-2-Octen-4-one	Ketones	9.30	1.18E-05
2,6,7-trimethyl-Decane	Hydrocarbons	2.68	1.86E-02
2,6,8-trimethyl-Decane	Hydrocarbons	2.68	1.86E-02
2,4,6-trimethyl-Decane	Hydrocarbons	2.68	1.86E-02
cis-1-methyl-4-(1-methylethenyl)-Cyclohexane	Hydrocarbons	2.91	1.10E-03
1-Iodoundecane	Hydrocarbons	3.32	3.21E-02
Undecane, 2-methyl-	Hydrocarbons	7.31	1.35E-04

1,2,3-trimethyl-Cyclopentene	Hydrocarbons	7.98	1.55E-04
2-propenylidene-Cyclobutene	Hydrocarbons	8.12	7.77E-04
Cyclopentane, nonyl-	Hydrocarbons	8.17	1.57E-03
octyl-Cyclohexane	Hydrocarbons	10.37	3.01E-02
2,3,6,7-tetramethyl-Octane	Hydrocarbons	44.10	1.14E-04
2,2-Dimethyl-6-vinyl-2H-chromene	Heterocyclic compounds	8.18	1.04E-02
3-ethyl-Pyridine	Heterocyclic compounds	8.32	8.43E-05
Pyrazine, trimethyl-	Heterocyclic compounds	8.62	1.44E-04
Acetylpyrazine	Heterocyclic compounds	8.85	1.53E-04
5-(1,1-dimethylethyl)-2,4(1H,3H)-Pyrimidinedione	Heterocyclic compounds	12.42	2.42E-02
1-Methyl-3-formylindole	Heterocyclic compounds	14.03	3.14E-02
Indole	Heterocyclic compounds	59.93	2.03E-02
sec-Butyl propyl carbonate	Esters	2.21	1.29E-03
Hexanoic acid, propyl ester	Esters	3.38	1.52E-02
Hexanoic acid, ethyl ester	Esters	7.58	1.24E-04
Carbamodithioic acid, diethyl-, methyl ester	Esters	8.40	6.14E-03
δ-Nonalactone	Esters	8.92	1.03E-02
Carbonic acid, nonyl prop-1-en-2-yl ester	Esters	11.04	1.72E-02
Propanoic acid, 3-ethoxy-, ethyl ester	Esters	22.81	1.15E-05
Octanoic acid, ethyl ester	Esters	34.96	2.06E-04
Triacetin	Esters	84.56	1.32E-02
p-Xylene	Aromatics	2.83	1.12E-03
Benzene, 1,3-dimethyl-	Aromatics	2.83	1.12E-03
o-Xylene	Aromatics	2.83	1.12E-03
para-Anisaldehyde diethyl acetal	Aromatics	10.78	3.14E-02
1-octenyl-Benzene	Aromatics	11.55	9.53E-03
m-Chloroaniline	Amines	8.41	8.30E-04
Diethyltoluamide	Amines	11.08	9.54E-03
4-methyl-Pentanamide	Amines	40.88	8.62E-05
2-Nonenal, (Z)-	Aldehydes	2.38	1.83E-03
Phenol, 2-butyl-	Aldehydes	8.01	4.89E-03
2,6,6-trimethyl-1-Cyclohexene-1-acetAldehyde	Aldehydes	10.68	4.85E-02
3-FurAldehyde	Aldehydes	14.17	1.16E-04
4-Nonanol	Alcohols	3.10	1.81E-03
2-Nonanol	Alcohols	3.10	1.81E-03
Cyclohexa-2,4-dienylmethanol	Alcohols	7.74	2.01E-04
2-(hydroxymethyl)-2-methyl-1,3-Propanediol	Alcohols	8.30	3.75E-04
Glycerin	Alcohols	17.18	2.93E-04
2-methyl-Octanoic acid	Acids	10.45	8.04E-03
3-hydroxy-Butanoic acid	Acids	26.58	6.32E-05

Table S3. The differentially changed volatile flavor compounds (DCVFCs) between F10 and F5.

DCVFCs	Class	Fold change	*P*-value
2,6-Dimethyl-2-trans-6-octadiene	Terpenoids	9.83	1.45E-04
γ-Muurolene	Terpenoids	5.14	1.37E-02
Benzene, 1-(1,5-dimethyl-4-hexenyl)-4-methyl-	Terpenoids	2.64	1.01E-03
7-Octylidenebicyclo[4.1.0]heptane	Terpenoids	2.36	2.81E-03
Dimethyl triSulfur compounds	Sulfur compounds	2.33	2.02E-03
1-iodo-Decane	Others	2.78	6.71E-03
3-Ethylcyclopentanone	Ketones	7.68	1.26E-04
1-(2-methyl-2-cyclopenten-1-yl)-Ethanone	Ketones	4.90	9.37E-04
4'-Butoxyacetophenone	Ketones	3.88	9.83E-04
2H-Indol-2-one, 1,3-dihydro-	Ketones	2.07	4.42E-03
2,7-dimethyl-2,6-Octadiene	Hydrocarbons	9.83	1.45E-04
2,6-Octadiene, 2,6-dimethyl-	Hydrocarbons	9.83	1.45E-04
cis-2,6-Dimethyl-2,6-octadiene	Hydrocarbons	9.83	1.45E-04
5-methyl-Nonane	Hydrocarbons	5.37	5.62E-04
(1-methylethylidene)-Cyclohexane	Hydrocarbons	3.82	1.95E-04
13-Oxabicyclo[10.1.0]tridecane	Hydrocarbons	2.88	1.56E-02
3-phenyl-Furan	Heterocyclic compounds	3.14	1.40E-02
1-Methyl-3-formylindole	Heterocyclic compounds	2.63	9.34E-03
2,2-Dimethyl-6-vinyl-2H-chromene	Heterocyclic compounds	2.08	8.79E-03
Heptanoic acid, methyl ester	Esters	4.08	3.26E-04
Ethyl {[(ethylthio)carbonyl]thio}acetate	Esters	3.14	8.11E-04
Octanoic acid, ethyl ester	Esters	2.92	5.67E-04
Carbonic acid, nonyl prop-1-en-2-yl ester	Esters	2.81	3.39E-03
2-(2-butoxyethoxy)-Ethanol,acetate	Esters	2.15	1.74E-02
Propanoic acid, 2-methyl-, 3-phenylpropyl ester	Esters	2.10	3.58E-03
1-octenyl-Benzene	Aromatics	4.29	1.71E-03
2-propenyl-Benzene	Aromatics	2.85	1.49E-03
N-ethyl-Cyclopentanamine	Amines	5.68	1.72E-03
Diethyltoluamide	Amines	3.56	1.65E-03
m-Chloroaniline	Amines	2.30	1.21E-03
N,N'-Diacetylethylenediamine	Amines	2.13	3.68E-02
N-propyl-Benzamide	Amines	2.12	3.80E-03
1-Heptanol	Alcohols	6.47	5.21E-03
2-methyl-Decanoic acid	Acids	7.95	4.46E-05
3-Hydroxydecanoic acid	Acids	2.79	1.51E-03
tetraethyl-Urea	Nitrogen compounds	0.24	6.30E-03
Octanoic Acid	Acids	0.23	1.06E-02
Ethanone, 1-(2-methyl-1-cyclopenten-1-yl)-	Ketones	0.20	9.66E-05
4-(1-methylethyl)-2-Cyclohexen-1-one	Ketones	0.17	7.05E-04
Isophorone	Ketones	0.17	6.40E-04
4-Allyl-1,6-heptadiene-4-ol	Alcohols	0.14	2.91E-04
Indole	Heterocyclic compounds	0.12	2.91E-02
Triacetin	Esters	0.09	1.75E-02
Glycerin	Alcohols	0.08	3.20E-04
Propanoic acid, 3-ethoxy-, ethyl ester	Esters	0.06	1.24E-05
3-hydroxy-Butanoic acid	Acids	0.05	6.70E-05
4-methyl-Pentanamide	Amines	0.03	8.94E-05

2,3,6,7-tetramethyl-Octane	Hydrocarbons	0.03	1.17E-04

Table S4. The differentially changed volatile flavor compounds (DCVFCs) between F20 and F10.

DCVFCs	Class	Fold change	*P*-value
octyl-Cyclohexane	Hydrocarbons	2.47	3.09E-03
tetrahydro-2,2-dimethyl-5-(1-methyl-1-propenyl)-Furan	Heterocyclic compounds	2.14	1.93E-02
Ethanone, 1-(2-methyl-1-cyclopenten-1-yl)-	Ketones	7.75	5.22E-06
Heptane, 3,3,4-trimethyl-	Hydrocarbons	6.03	2.86E-03
Triacetin	Esters	6.71	9.82E-04
2,3,6,7-tetramethyl-Octane	Hydrocarbons	6.04	1.07E-06
3,4-bis(1,1-dimethylethyl)-2,2,5,5-tetramethyl-Hexane	Hydrocarbons	5.21	1.60E-04
2-methyl-5-(methylthio)-Furan	Heterocyclic compounds	6.06	1.59E-04
Humulene	Terpenoids	10.99	2.75E-04
4-(1-methylethyl)-2-Cyclohexen-1-one	Ketones	8.07	2.74E-04
Isophorone	Ketones	6.50	1.36E-04
4-ethyl-Decane	Hydrocarbons	2.12	2.61E-03
Propanoic acid, 3-ethoxy-, ethyl ester	Esters	5.30	1.54E-05
2-methyl-Butanoic acid,2-methyl-2-propenyl ester	Esters	2.32	2.30E-05
2-Tridecanol	Alcohols	3.29	2.99E-03
4-Propoxy-2-butanone	Ketones	7.91	4.45E-03
Indole	Heterocyclic compounds	7.07	6.52E-04
Ethyl 4-oxo-2-phenylpentanoate	Esters	2.20	8.14E-04
para-Anisaldehyde diethyl acetal	Aromatics	2.35	1.05E-02
tetraethyl-Urea	Nitrogen compounds	3.85	8.56E-03
4-Allyl-1,6-heptadiene-4-ol	Alcohols	7.97	3.07E-06
Butanoic acid, 3-methyl-, butyl ester	Esters	2.02	2.26E-03
4-methyl-Pentanamide	Amines	6.04	1.37E-03
Glycerin	Alcohols	5.54	1.61E-04
3-hydroxy-Butanoic acid	Acids	5.20	3.61E-05
Octanoic Acid	Acids	4.11	3.88E-03
γ-Muurolene	Terpenoids	0.35	2.05E-02
Dimethyl triSulfur compounds	Sulfur compounds	0.27	5.84E-04
Phenol, 3,5-dimethyl-	Phenols	0.44	1.73E-03
1-iodo-Decane	Others	0.43	8.77E-03
1-(2-methyl-2-cyclopenten-1-yl)-Ethanone	Ketones	0.25	1.29E-03
13-Oxabicyclo[10.1.0]tridecane	Hydrocarbons	0.43	1.10E-02
2-propenylidene-Cyclobutene	Hydrocarbons	0.36	4.70E-02
(1-methylethylidene)-Cyclohexane	Hydrocarbons	0.27	8.00E-05
4-Quinolinecarboxaldehyde	Heterocyclic compounds	0.49	2.03E-03
1-Methyl-3-formylindole	Heterocyclic compounds	0.46	1.01E-02
2,5-Dimethylfuran-3-thiol	Heterocyclic compounds	0.44	6.95E-04
5-(1,1-dimethylethyl)-2,4(1H,3H)-Pyrimidinedione	Heterocyclic compounds	0.28	4.21E-02
Quinazoline, 4-methyl-	Heterocyclic compounds	0.22	1.48E-03
Quinoxaline, 2-methyl-	Heterocyclic compounds	0.22	1.48E-03
Pyrimidine, 4-butyl-3,4-dihydro-5-methyl-	Heterocyclic compounds	0.12	5.85E-03
2-(2-butoxyethoxy)-Ethanol,acetate	Esters	0.45	4.87E-03
Octanoic acid, ethyl ester	Esters	0.28	3.05E-04
Heptanoic acid, methyl ester	Esters	0.17	2.03E-04
Benzene, (1-methylethyl)-	Aromatics	0.14	9.13E-05
m-Chloroaniline	Amines	0.49	9.95E-04
2-Nonenal, (Z)-	Aldehydes	0.14	9.96E-06

3-Phenylpropanol	Alcohols	0.17	3.30E-05
Cyclohexa-2,4-dienylmethanol	Alcohols	0.14	7.47E-05
2-methyl-Decanoic acid	Acids	0.13	4.51E-05

Table S5. The differentially changed volatile flavor compounds (DCVFCs) between F20 and F0.

DCVFCs	Class	Fold change	*P*-value
Triacetin	Esters	52.57	5.60E-04
Indole	Heterocyclic compounds	51.75	3.77E-04
1-octenyl-Benzene	Aromatics	40.47	6.51E-05
Diethyltoluamide	Amines	34.69	8.77E-05
Carbonic acid, nonyl prop-1-en-2-yl ester	Esters	30.21	9.15E-05
2-Tridecanol	Alcohols	29.69	8.51E-04
octyl-Cyclohexane	Hydrocarbons	29.66	4.62E-04
para-Anisaldehyde diethyl acetal	Aromatics	28.28	1.54E-03
Octanoic acid, ethyl ester	Esters	28.13	2.79E-05
2,2-Dimethyl-6-vinyl-2H-chromene	Heterocyclic compounds	18.14	9.11E-04
Cyclopentane, nonyl-	Hydrocarbons	17.47	1.38E-04
1-Methyl-3-formylindole	Heterocyclic compounds	16.97	8.79E-03
2-methyl-Octanoic acid	Acids	15.73	1.45E-03
Humulene	Terpenoids	14.95	2.47E-04
2,6,6-trimethyl-1-Cyclohexene-1-acetAldehyde	Aldehydes	14.39	2.83E-04
Acetylpyrazine	Heterocyclic compounds	11.98	3.64E-05
Pyrazine, trimethyl-	Heterocyclic compounds	11.65	3.38E-05
δ-Nonalactone	Esters	11.16	1.35E-03
Undecane, 2-methyl-	Hydrocarbons	11.09	6.53E-04
4-Propoxy-2-butanone	Ketones	10.70	3.87E-03
Hexathiane	Sulfur compounds	10.39	3.89E-02
Hexanoic acid, ethyl ester	Esters	10.16	1.27E-05
3-FurAldehyde	Aldehydes	9.98	2.09E-03
methoxy-phenyl-_Oxime-	Nitrogen compounds	9.71	6.99E-05
3-ethyl-Pyridine	Heterocyclic compounds	9.51	2.79E-05
m-Chloroaniline	Amines	9.41	5.81E-05
Carbamodithioic acid, diethyl-, methyl ester	Esters	9.16	5.81E-05
1-Iodoundecane	Hydrocarbons	8.33	1.27E-03
2,3,6,7-tetramethyl-Octane	Hydrocarbons	8.19	5.77E-07
4-methyl-Pentanamide	Amines	8.18	1.12E-03
1,2,3-trimethyl-Cyclopentene	Hydrocarbons	8.17	4.19E-05
Heptane, 3,3,4-trimethyl-	Hydrocarbons	8.16	2.35E-03
Glycerin	Alcohols	7.51	1.24E-04
Dimethyl triSulfur compounds	Sulfur compounds	7.35	4.61E-04
Phenol, 4-butyl-	Phenols	7.27	3.05E-04
Phenol, 2-butyl-	Aldehydes	7.27	3.05E-04
Propanoic acid, 3-ethoxy-, ethyl ester	Esters	7.19	1.06E-05
3-hydroxy-Butanoic acid	Acids	7.05	2.59E-05
6Z-2,5,5,10-Tetramethyl-undeca-2,6,9-trien-8-one	Terpenoids	6.76	2.47E-03
1-iodo-Decane	Others	6.45	2.67E-04
4'-Butoxyacetophenone	Ketones	4.87	3.81E-04
Hexanoic acid, propyl ester	Esters	4.32	8.37E-03
4-Nonanol	Alcohols	4.27	2.73E-03
2-Nonanol	Alcohols	4.27	2.73E-03
Ethyl {[(ethylthio)carbonyl]thio}acetate	Esters	3.97	4.94E-04
cis-1-methyl-4-(1-methylethenyl)-Cyclohexane	Hydrocarbons	3.78	1.47E-02
Oxalic acid, isobutyl neopentyl ester	Acids	3.50	1.83E-04

Propanoic acid, 2-methyl-, 3-phenylpropyl ester	Esters	3.49	5.63E-04
p-Xylene	Aromatics	3.29	2.23E-04
Benzene, 1,3-dimethyl-	Aromatics	3.29	2.23E-04
o-Xylene	Aromatics	3.29	2.23E-04
2H-Indol-2-one, 1,3-dihydro-	Ketones	3.25	1.08E-03
4-ethyl-Decane	Hydrocarbons	3.23	6.28E-04
3-Hydroxydecanoic acid	Acids	3.06	7.47E-04
Cyclohexanone, 5-methyl-2-(1-methylethyl)-	Terpenoids	2.59	5.74E-04
(2R-cis)-5-methyl-2-(1-methylethyl)-Cyclohexanone	Ketones	2.59	5.74E-04
sec-Butyl propyl carbonate	Esters	2.52	1.12E-03
N-propyl-Benzamide	Amines	2.40	1.95E-03
heptyl-Benzene	Aromatics	2.16	2.10E-03
2-(2-butoxyethoxy)-Ethanol,acetate	Esters	2.14	1.55E-02
3-methyl-Tetradecane	Hydrocarbons	2.13	1.32E-02
1-Pentadecene	Hydrocarbons	2.06	2.13E-02
Nonaneperoxoic acid, 1,1-dimethylethyl ester	Esters	2.02	8.62E-03
3-Methylbutan-2-yl (E)-2-methylbut-2-enoate	Acids	0.49	6.93E-04
Pyrazine, 2,5-dimethyl-3-(3-methylbutyl)-	Heterocyclic compounds	0.49	3.15E-03
3,7,7-trimethyl-Bicyclo[4.1.0]heptane	Hydrocarbons	0.49	8.19E-04
2,3-dimethyl-Heptane	Hydrocarbons	0.48	3.97E-03
5H-Tetrazol-5-Amine	Amines	0.48	6.03E-04
1-methyl-3-(2-methyl-2-propenyl)-Cyclopentane	Hydrocarbons	0.48	1.17E-04
1,3,6-Octatriene, 3,7-dimethyl-, (Z)-	Terpenoids	0.48	1.02E-04
trans-β-Ocimene	Terpenoids	0.48	1.02E-04
4-Amino-4,5(1H)-dihydro-1,2,4-triazole-5-one	Ketones	0.48	3.58E-03
2-Butanone, 4-(2,6,6-trimethyl-1-cyclohexen-1-yl)-	Ketones	0.48	7.06E-03
4-Cyanocyclohexene	Nitrogen compounds	0.48	3.44E-04
7-Oxabicyclo[4.1.0]heptane, 1-methyl-4-(1-methylethenyl)-	Terpenoids	0.48	5.81E-04
6-methyl-2,4-Heptanedione	Ketones	0.48	8.01E-04
3,5,9-Undecatrien-2-one, 6,10-dimethyl-	Ketones	0.48	4.68E-02
β-Ocimene	Terpenoids	0.47	6.89E-05
α-Farnesene	Terpenoids	0.47	6.60E-03
Maleic hydrazide	Nitrogen compounds	0.47	9.02E-04
2,6-Dimethyl-1,3,5,7-octatetraene, E,E-	Terpenoids	0.47	6.37E-05
Ethanone, 1-(1H-pyrazol-4-yl)-	Ketones	0.47	1.43E-03
β-Pinene	Terpenoids	0.47	3.77E-05
α-Phellandrene 1	Terpenoids	0.47	3.77E-05
1,3,5,7-Cyclooctatetraene	Esters	0.47	1.45E-04
Tetracyclo[6.2.1.0(2,4).0(4,7)]undecane	Hydrocarbons	0.47	6.55E-05
4-Vinyl-imidazole	Heterocyclic compounds	0.46	6.15E-05
3,5-Octadien-2-one, (E,E)-	Ketones	0.46	9.01E-05
3,5-Octadien-2-one	Ketones	0.46	9.01E-05
N1-(4-fluorobenzyl)-N2,N2-dimethyl-1,2-ethanediamine	Amines	0.46	2.42E-03
Biphenyl	Aromatics	0.46	2.05E-03
1,3-Benzodioxole	Heterocyclic compounds	0.46	6.36E-04
α-Muurolene	Terpenoids	0.46	3.82E-03
2-Butoxyethyl acetate	Esters	0.45	6.55E-04
Methyl trans-2-(3-cyclopropyl-7-norcaranyl)acetate	Esters	0.45	2.07E-03
Furan, 2-(1-pentenyl)-, (E)-	Heterocyclic compounds	0.45	1.04E-03

cis-2-(2-Pentenyl)furan	Heterocyclic compounds	0.45	1.04E-03
2-ethyl-4-methyl-1-Pentanol	Alcohols	0.44	3.75E-02
1,5,5-trimethyl-3-methylene-Cyclohexene	Hydrocarbons	0.44	1.98E-03
1,5,5-Trimethyl-6-methylene-cyclohexene	Hydrocarbons	0.44	1.98E-03
(E)-8-methyl-5-Undecene	Hydrocarbons	0.44	2.35E-04
2,2-dihydroxy-1-phenyl-Ethanone	Ketones	0.43	2.35E-03
3-Ethylcyclopentanone	Ketones	0.42	2.48E-02
Cyclohexene, 4-methyl-1-(1-methylethenyl)-	Hydrocarbons	0.42	4.79E-02
N-(2-Pyridinylmethyl)-1-butanamine, N-acetyl-	Heterocyclic compounds	0.41	7.72E-03
Phenol	Phenols	0.41	1.59E-04
α-Methylstyrene	Aromatics	0.40	4.95E-05
3-Cyclohexene-1-ethanol	Alcohols	0.39	3.06E-04
trans-1,3-dichloro-Cyclopentane	Halogenated hydrocarbons	0.39	5.05E-05
cis-1,3-dichloro-Cyclopentane	Halogenated hydrocarbons	0.39	5.05E-05
1-(1-cyclohexen-1-yl)-Ethanone	Ketones	0.38	5.30E-04
Vinyl 10-undecenoate	Esters	0.38	3.02E-02
1,3,8-p-Menthatriene	Terpenoids	0.37	2.93E-04
2-propenyl-Benzene	Aromatics	0.36	4.45E-06
1,4-ButanediAmine	Amines	0.36	6.00E-05
p-Cresol	Aromatics	0.36	8.56E-05
Phenol, 3-methyl-	Aromatics	0.36	8.56E-05
dl-Menthol	Terpenoids	0.35	2.65E-04
4H-1,2,4-Triazol-4-amine	Heterocyclic compounds	0.34	6.20E-05
Hexanoic Acid	Acids	0.34	3.83E-02
1-Tetrazol-2-ylethanone	Heterocyclic compounds	0.34	6.20E-04
Phenol, 3,5-dimethyl-	Phenols	0.33	1.65E-04
Isovaleric acid, 3-methylbutyl-2 ester	Esters	0.33	4.01E-05
β-Myrcene	Terpenoids	0.33	1.55E-05
6-hydroxy-2-Hexanone	Ketones	0.33	8.56E-05
3-Octanol	Alcohols	0.32	4.60E-04
1-methyl-Piperazine	Heterocyclic compounds	0.31	3.00E-05
2,6-Dimethyl-2-trans-6-octadiene	Terpenoids	0.31	4.84E-05
2,7-dimethyl-2,6-Octadiene	Hydrocarbons	0.31	4.84E-05
cis-2,6-Dimethyl-2,6-octadiene	Hydrocarbons	0.31	4.84E-05
2,6-Octadiene, 2,6-dimethyl-	Hydrocarbons	0.31	4.84E-05
4-Hexen-1-ol, acetate	Others	0.31	4.34E-04
Ethanone, 1-(2-methyl-1-cyclopenten-1-yl)-	Ketones	0.30	9.05E-04
Phenylethyl Alcohol	Alcohols	0.30	7.26E-04
3-methyl-Butanamide	Amines	0.30	2.95E-05
Octane, 5-ethyl-2-methyl-	Hydrocarbons	0.30	1.06E-04
6-ethyl-2-methyl-Octane	Hydrocarbons	0.30	1.06E-04
6-methyl-3-Heptanone	Ketones	0.29	2.29E-02
7-methyl-5-Octen-4-one	Ketones	0.29	1.08E-04
4(1H)-Pyridinone, 2,3-dihydro-1-methyl-	Heterocyclic compounds	0.29	9.50E-05
2-Octanone	Ketones	0.29	1.37E-05
5-Hepten-2-one, 6-methyl-	Ketones	0.29	5.22E-05
2-ethyl-2-Hexenal	Aldehydes	0.28	9.75E-05
Caryophyllene	Terpenoids	0.28	1.54E-03
1-(2-methyl-2-cyclopenten-1-yl)-Ethanone	Ketones	0.28	1.24E-04

1-chloro-Dodecane	Halogenated hydrocarbons	0.27	4.14E-03
propyl-Cyclohexane	Hydrocarbons	0.27	5.03E-05
α-Cubebene	Terpenoids	0.27	9.64E-04
1-Methylimidazole-5-carboxaldehyde	Heterocyclic compounds	0.27	1.50E-05
2-Nonenal, (Z)-	Aldehydes	0.27	1.79E-05
Benzenemethanol, α-2-cyclohexen-1-yl-	Alcohols	0.27	1.67E-03
1,2,3-trimethoxy-Propane	Hydrocarbons	0.27	8.07E-04
2,3,4-Trifluorobenzoic acid, cyclobutyl ester	Esters	0.26	2.15E-04
2-Methyl-1-nonene-3-yne	Hydrocarbons	0.26	2.89E-05
Furan, 2-butyltetrahydro-	Heterocyclic compounds	0.25	1.63E-04
(1-methylethylidene)-Cyclohexane	Hydrocarbons	0.24	1.85E-05
2-Methyl-3-furanthiol	Heterocyclic compounds	0.24	2.90E-04
7-methyl-1-Undecene	Hydrocarbons	0.24	9.89E-05
3,4-bis(1,1-dimethylethyl)-2,2,5,5-tetramethyl-Hexane	Hydrocarbons	0.22	1.91E-03
1,3-Hexadiene, 3-ethyl-2-methyl-	Hydrocarbons	0.21	1.07E-02
Heptanoic acid, methyl ester	Esters	0.20	9.48E-05
α-Ionone	Terpenoids	0.20	1.72E-03
2-methyl-Decanoic acid	Acids	0.18	8.56E-04
Sorbic acid vinyl ester	Esters	0.17	6.25E-03
Acetyl valeryl	Ketones	0.16	9.82E-07
1-ethyl-Cyclohexene	Hydrocarbons	0.16	1.32E-05
2-methyl-5-(methylthio)-Furan	Heterocyclic compounds	0.15	5.87E-05
3-Phenylpropanol	Alcohols	0.15	1.14E-04
5-methyl-1H-Indole	Heterocyclic compounds	0.13	1.17E-04
7-methyl-1H-Indole	Heterocyclic compounds	0.13	1.17E-04
Estragole	Aromatics	0.12	7.41E-05
DiSulfur compounds, methyl 2-propenyl	Sulfur compounds	0.11	2.55E-05
Benzene, (1-methylethyl)-	Aromatics	0.10	6.12E-05
2,4-dimethyl-2,4-Heptadiene	Hydrocarbons	0.06	1.90E-04
Pyrimidine, 4-butyl-3,4-dihydro-5-methyl-	Heterocyclic compounds	0.06	1.85E-05

Table S6. The r-value of Pearson's correlation analysis between differentially changed volatile flavor compounds and microorganisms.

r-Value	*Lactobacilli*	*Bacillus*	*Enterococcus*	*Myroides*	*Staphylococcus*	*Fusarium*	*Aspergillus*	*Hanseniaspora*	*Debaryomyces*	*Arthrinium*	DCVFC1	DCVFC2	DCVFC3	DCVFC4	DCVFC5	DCVFC6	DCVFC7	DCVFC8	DCVFC9	DCVFC10	DCVFC11	DCVFC12	DCVFC13	DCVFC14	DCVFC15	DCVFC16	DCVFC17	DCVFC18	DCVFC19	DCVFC20	DCVFC21
Lactobacilli	1.00	−0.80	−0.74	−0.37	−0.33	0.94	0.90	−0.61	−0.57	−0.26	−0.38	−0.55	−0.98	−0.83	−0.70	−0.55	−0.37	−0.98	0.79	0.95	0.92	0.83	0.89	0.90	0.91	0.96	0.95	0.66	0.88	0.94	0.75
Bacillus	−0.80	1.00	0.66	−0.18	0.59	−0.77	−0.73	0.31	0.76	−0.28	0.57	0.76	0.81	0.91	0.32	0.76	0.62	0.81	−0.61	−0.64	−0.58	−0.57	−0.72	−0.72	−0.71	−0.65	−0.72	−0.20	−0.70	−0.63	−0.67
Enterococcus	−0.74	0.66	1.00	−0.07	−0.12	−0.51	−0.46	0.19	0.27	−0.11	−0.13	0.86	0.82	0.48	0.66	0.86	−0.10	0.82	−0.67	−0.62	−0.55	−0.31	−0.41	−0.42	−0.45	−0.61	−0.70	−0.72	−0.40	−0.52	−0.93
Myroides	−0.37	−0.18	−0.07	1.00	0.02	−0.45	−0.45	0.74	−0.07	0.97	0.11	−0.51	0.31	0.12	0.61	−0.51	0.03	0.31	−0.18	−0.55	−0.59	−0.67	−0.51	−0.51	−0.50	−0.55	−0.38	−0.51	−0.52	−0.60	0.10
Staphylococcus	−0.33	0.59	−0.12	0.02	1.00	−0.58	−0.59	0.40	0.77	−0.08	0.99	0.03	0.29	0.78	−0.14	0.03	0.99	0.30	−0.09	−0.27	−0.27	−0.57	−0.63	−0.62	−0.56	−0.30	−0.27	0.44	−0.62	−0.39	0.17
Fusarium	0.94	−0.77	−0.51	−0.45	−0.58	1.00	0.94	−0.70	−0.70	−0.34	−0.63	−0.36	−0.90	−0.91	−0.58	−0.36	−0.62	−0.90	0.69	0.92	0.90	0.93	0.97	0.98	0.95	0.93	0.87	0.45	0.96	0.96	0.50
Aspergillus	0.90	−0.73	−0.46	−0.45	−0.59	0.94	1.00	−0.69	−0.68	−0.34	−0.63	−0.28	−0.85	−0.88	−0.54	−0.28	−0.62	−0.85	0.64	0.85	0.85	0.91	0.95	0.95	0.99	0.87	0.80	0.39	0.93	0.89	0.44
Hanseniaspora	−0.61	0.31	0.19	0.74	0.40	−0.70	−0.69	1.00	0.19	0.71	0.48	−0.16	0.58	0.54	0.70	−0.16	0.35	0.59	−0.23	−0.66	−0.67	−0.86	−0.74	−0.73	−0.71	−0.67	−0.61	−0.43	−0.74	−0.76	−0.08
Debaryomyces	−0.57	0.76	0.27	−0.07	0.77	−0.70	−0.68	0.19	1.00	−0.20	0.77	0.43	0.55	0.86	−0.02	0.42	0.85	0.55	−0.48	−0.48	−0.47	−0.53	−0.70	−0.70	−0.67	−0.51	−0.48	0.15	−0.69	−0.53	−0.27
Arthrinium	−0.26	−0.28	−0.11	0.97	−0.08	−0.34	−0.34	0.71	−0.20	1.00	0.01	−0.56	0.22	−0.01	0.62	−0.56	−0.09	0.22	−0.12	−0.45	−0.51	−0.57	−0.39	−0.39	−0.38	−0.44	−0.27	−0.50	−0.40	−0.49	0.15
DCVFC1	−0.38	0.57	−0.13	0.11	0.99	−0.63	−0.63	0.48	0.77	0.01	1.00	−0.01	0.32	0.80	−0.09	−0.01	0.98	0.33	−0.13	−0.33	−0.34	−0.64	−0.68	−0.67	−0.62	−0.36	−0.33	0.38	−0.67	−0.46	0.16
DCVFC2	−0.55	0.76	0.86	−0.51	0.03	−0.36	−0.28	−0.16	0.43	−0.56	−0.01	1.00	0.62	0.49	0.24	1.00	0.07	0.62	−0.57	−0.36	−0.29	−0.06	−0.25	−0.25	−0.26	−0.36	−0.51	−0.33	−0.23	−0.27	−0.86
DCVFC3	−0.98	0.81	0.82	0.31	0.29	−0.90	−0.85	0.58	0.55	0.22	0.32	0.62	1.00	0.81	0.76	0.63	0.33	1.00	−0.78	−0.91	−0.88	−0.77	−0.85	−0.85	−0.86	−0.92	−0.92	−0.68	−0.84	−0.88	−0.79
DCVFC4	−0.83	0.91	0.48	0.12	0.78	−0.91	−0.88	0.54	0.86	−0.01	0.80	0.49	0.81	1.00	0.34	0.49	0.81	0.81	−0.56	−0.72	−0.70	−0.79	−0.89	−0.90	−0.87	−0.75	−0.75	−0.15	−0.88	−0.78	−0.45
DCVFC5	−0.70	0.32	0.66	0.61	−0.14	−0.58	−0.54	0.70	−0.02	0.62	−0.09	0.24	0.76	0.34	1.00	0.24	−0.14	0.75	−0.45	−0.71	−0.69	−0.59	−0.55	−0.55	−0.56	−0.70	−0.65	−0.86	−0.55	−0.68	−0.55
DCVFC6	−0.55	0.76	0.86	−0.51	0.03	−0.36	−0.28	−0.16	0.42	−0.56	−0.01	1.00	0.63	0.49	0.24	1.00	0.07	0.62	−0.57	−0.36	−0.29	−0.06	−0.25	−0.25	−0.26	−0.36	−0.51	−0.33	−0.23	−0.27	−0.86
DCVFC7	−0.37	0.62	−0.10	0.03	0.99	−0.62	−0.62	0.35	0.85	−0.09	0.98	0.07	0.33	0.81	−0.14	0.07	1.00	0.33	−0.17	−0.32	−0.31	−0.57	−0.66	−0.65	−0.60	−0.35	−0.30	0.41	−0.65	−0.42	0.11
DCVFC8	−0.98	0.81	0.82	0.31	0.30	−0.90	−0.85	0.59	0.55	0.22	0.33	0.62	1.00	0.81	0.75	0.62	0.33	1.00	−0.78	−0.92	−0.88	−0.77	−0.85	−0.86	−0.86	−0.92	−0.92	−0.68	−0.84	−0.89	−0.79
DCVFC9	0.79	−0.61	−0.67	−0.18	−0.09	0.69	0.64	−0.23	−0.48	−0.12	−0.13	−0.57	−0.78	−0.56	−0.45	−0.57	−0.17	−0.78	1.00	0.83	0.84	0.58	0.67	0.68	0.68	0.82	0.81	0.62	0.68	0.72	0.80
DCVFC10	0.95	−0.64	−0.62	−0.55	−0.27	0.92	0.85	−0.66	−0.48	−0.45	−0.33	−0.36	−0.91	−0.72	−0.71	−0.36	−0.32	−0.92	0.83	1.00	0.99	0.89	0.91	0.92	0.90	1.00	0.96	0.72	0.91	0.97	0.67
DCVFC11	0.92	−0.58	−0.55	−0.59	−0.27	0.90	0.85	−0.67	−0.47	−0.51	−0.34	−0.29	−0.88	−0.70	−0.69	−0.29	−0.31	−0.88	0.84	0.99	1.00	0.90	0.91	0.91	0.90	0.99	0.93	0.70	0.91	0.97	0.62
DCVFC12	0.83	−0.57	−0.31	−0.67	−0.57	0.93	0.91	−0.86	−0.53	−0.57	−0.64	−0.06	−0.77	−0.79	−0.59	−0.06	−0.57	−0.77	0.58	0.89	0.90	1.00	0.97	0.97	0.94	0.90	0.83	0.44	0.97	0.94	0.31
DCVFC13	0.89	−0.72	−0.41	−0.51	−0.63	0.97	0.95	−0.74	−0.70	−0.39	−0.68	−0.25	−0.85	−0.89	−0.55	−0.25	−0.66	−0.85	0.67	0.91	0.91	0.97	1.00	1.00	0.97	0.92	0.86	0.40	1.00	0.94	0.43

DCVFC 14	0.90	–0.72	–0.42	–0.51	–0.62	0.98	0.95	–0.73	–0.70	–0.39	–0.67	–0.25	–0.85	–0.90	–0.55	–0.25	–0.65	–0.86	0.68	0.92	0.91	0.97	1.00	1.00	0.98	0.93	0.87	0.41	1.00	0.95	0.44
DCVFC 15	0.91	–0.71	–0.45	–0.50	–0.56	0.95	0.99	–0.71	–0.67	–0.38	–0.62	–0.26	–0.86	–0.87	–0.56	–0.26	–0.60	–0.86	0.68	0.90	0.90	0.94	0.97	0.98	1.00	0.91	0.85	0.44	0.97	0.92	0.46
DCVFC 16	0.96	–0.65	–0.61	–0.55	–0.30	0.93	0.87	–0.67	–0.51	–0.44	–0.36	–0.36	–0.92	–0.75	–0.70	–0.36	–0.35	–0.92	0.82	1.00	0.99	0.90	0.92	0.93	0.91	1.00	0.95	0.70	0.92	0.98	0.65
DCVFC 17	0.95	–0.72	–0.70	–0.38	–0.27	0.87	0.80	–0.61	–0.48	–0.27	–0.33	–0.51	–0.92	–0.75	–0.65	–0.51	–0.30	–0.92	0.81	0.96	0.93	0.83	0.86	0.87	0.85	0.95	1.00	0.70	0.87	0.93	0.76
DCVFC 18	0.66	–0.20	–0.72	–0.51	0.44	0.45	0.39	–0.43	0.15	–0.50	0.38	–0.33	–0.68	–0.15	–0.86	–0.33	0.41	–0.68	0.62	0.72	0.70	0.44	0.40	0.41	0.44	0.70	0.70	1.00	0.41	0.63	0.73
DCVFC 19	0.88	–0.70	–0.40	–0.52	–0.62	0.96	0.93	–0.74	–0.69	–0.40	–0.67	–0.23	–0.84	–0.88	–0.55	–0.23	–0.65	–0.84	0.68	0.91	0.91	0.97	1.00	1.00	0.97	0.92	0.87	0.41	1.00	0.93	0.43
DCVFC 20	0.94	–0.63	–0.52	–0.60	–0.39	0.96	0.89	–0.76	–0.53	–0.49	–0.46	–0.27	–0.88	–0.78	–0.68	–0.27	–0.42	–0.89	0.72	0.97	0.97	0.94	0.94	0.95	0.92	0.98	0.93	0.63	0.93	1.00	0.54
DCVFC 21	0.75	–0.67	–0.93	0.10	0.17	0.50	0.44	–0.08	–0.27	0.15	0.16	–0.86	–0.79	–0.45	–0.55	–0.86	0.11	–0.79	0.80	0.67	0.62	0.31	0.43	0.44	0.46	0.65	0.76	0.73	0.43	0.54	1.00

Note: DCVFC1: dimethyltrisulfide, DCVFC2: cyclohexanone,5-methyl-2-(1-methylethyl)-, DCVFC3: acetylpyrazine, DCVFC4: hexanoic acid,ethyl ester, DCVFC5: indole, DCVFC6: (2R-cis)-5-methyl-2-(1-methylethyl)-cyclohexanone, DCVFC7: octanoic acid,ethyl ester, DCVFC8: pyrazine,trimethyl-, DCVFC9: 1,3,8-p-menthatriene, DCVFC10: trans-β-ocimene, DCVFC11: 3,5-octadien-2-one,(E,E)-, DCVFC12: β-myrcene, DCVFC13: 5-hepten-2-one,6-methyl-, DCVFC14: 2-octanone, DCVFC15: α-ionone, DCVFC16: β-pinene, DCVFC17: phenylethylalcohol, DCVFC18: heptanoic acid,methyl ester, DCVFC19: 3-octanol, DCVFC20: dl-menthol, DCVFC21: 3-phenylpropanol
